# SpatioCAD: Context-aware graph diffusion model for pinpointing spatially variable genes in heterogeneous tissues

**DOI:** 10.1371/journal.pcbi.1014923

**Published:** 2026-11-02

**Authors:** Hao Wen, Qunlun Shen, Shuqin Zhang

**Affiliations:** 1 School of Mathematical Sciences, Fudan University, Shanghai, China; 2 Department of Cardiology, First Affiliated Hospital of Ningbo University (Ningbo First Hospital), School of Medicine, Ningbo University, Ningbo, China; 3 Center for Applied Mathematics, Center for Integrative Spatial-Omics Research, and Shanghai Key Laboratory for Contemporary Applied Mathematics, Fudan University, Shanghai, China; Duke University School of Medicine, UNITED STATES OF AMERICA

## Abstract

Spatial transcriptomics enables comprehensive characterization of tissue architecture, and the identification of spatially variable genes (SVGs) is a critical step for defining region-specific molecular markers and uncovering spatially regulated mechanisms across diverse biological contexts. However, most existing methods for SVG detection overlook cell density variations, a major confounding factor in complex tissues such as tumors, where heterogeneous cellularity frequently introduces false-positive calls. Here we present SpatioCAD, a computational framework that explicitly decouples genuine spatial expression patterns from confounding effects driven by cellularity. SpatioCAD introduces a graph diffusion model that inherently incorporates cell-density-aware conditions to simulate expression propagation, supporting the identification of SVGs across a broad range of expression levels. Systematic evaluations on simulated datasets demonstrate its superior statistical power and specificity. Applied to breast cancer, lung cancer, and glioma datasets, SpatioCAD identifies functionally diverse SVGs, including low‑abundance transcripts with established roles in tumor progression, while also recapitulates biologically meaningful tissue architecture features. Importantly, SpatioCAD is fast and scalable to large tissues.

## Author summary

Spatial transcriptomics maps where genes are active in tissues, providing a way to explore how tissue organization relates to biological function and disease. Interpreting these maps is challenging when different regions contain very different numbers of cells, as is common in tumors. Such differences can influence observed gene expression patterns and complicate the identification of informative spatial variation. We developed SpatioCAD to identify genes with spatially patterned expression while accounting for density-related variation. Using simulated data and datasets from human cancers and normal brain tissue, we show that SpatioCAD identifies genes with coherent spatial patterns across a broad range of expression levels. In cancer tissues, these genes represent diverse biological functions, providing a complementary view of molecular differences within the tumor environment. By supporting the detection of patterns in genes with both high and low expression, SpatioCAD helps researchers explore tissue heterogeneity more broadly. Our work provides a computational tool for investigating the molecular organization of complex tissues and generating hypotheses for further biological study.

## Introduction

Spatial transcriptomics (ST) technologies enable the comprehensive gene expression profiling at near- or sub-cellular resolution within the native tissue context, providing unprecedented insights into the spatial organization of cells, their in situ functional states, the cell-cell communications and spatially distinct gene expression programs [1–4]. They have been widely applied to unravel the complex cellular interactions and delineate spatially organized functional domains that govern a broad spectrum of fundamental biological processes, from development to disease [5–8]. In particular, ST facilitates the systematic characterization of pathological tissue architectures, such as tumor microenvironments and spatially restricted malignant niches, through the analysis of spatially resolved human disease atlases [8].

A fundamental task in spatial transcriptomics data analysis is the identification of genes exhibiting spatially structured expression patterns [4,9]. These genes, known as spatially variable genes (SVGs), play a critical role in deciphering functionally diverse tissue regions and revealing the key molecular drivers of biological processes [10,11]. For instance, SVG analysis has uncovered the distinct spatial-pathological mechanisms underlying amyloid-β and τ proteins in Alzheimer’s disease [12]. Consequently, numerous computational methods have been proposed for SVG identification, each founded on distinct mathematical and statistical principles. For example, SPARK-X [13] and SpatialDE [4] employ statistical frameworks, such as Gaussian process regression and nonparametric approaches, to test spatial variance and dependence of gene expression; SpaGFT [14] utilizes a graph Fourier transform on spatial neighbor networks to decompose gene expression signals into frequency components, effectively highlighting regionally structured variation; Sepal [15] leverages diffusion-based smoothing and spatial autocorrelation analysis to enhance the detection of genes exhibiting coherent local expression domains. Scalable alternatives include nnSVG [16] and SOMDE [17]. nnSVG achieves linear scaling with the number of spatial locations through nearest-neighbor Gaussian processes, but its gene-wise model fitting can still be computationally demanding for large transcriptome-wide datasets. SOMDE accelerates Gaussian-process modelling by aggregating neighboring spatial locations into self-organizing map nodes, but this compression may lead to the loss of local spatial detail.

While existing SVG identification methods have been proven effective in well-structured tissues, their reliability is severely compromised in highly heterogeneous contexts such as the tumor microenvironment (TME). A key feature of the TME is the dramatic spatial variation in cell density resulting from the uncontrolled proliferation of cancer cells [18], which can be misinterpreted as genuine biological signal, thereby constituting a major confounding factor for SVG identification. Failing to account for this confounder risks missing key drivers of tissue function. Although one recent method, STMiner, employs a framework based on Optimal Transport (OT) to address this challenge [19], its high computational cost and sensitivity to outliers limit broader applications [20,21]. Furthermore, a prevalent bias in many current algorithms is the tendency to favor genes with high expression levels, frequently leading to the exclusion of key regulatory genes that are often expressed at low levels [22].

To overcome these challenges, we present SpatioCAD (SPAtially variable gene identification using Context-Aware graph Diffusion model), a computational framework built on our proposed Node-Attributed Graph Diffusion (NAGD) model to explicitly decouple genuine spatial patterns from cellular density variations. When benchmarked on simulated datasets, SpatioCAD achieves high true-SVG recovery with low realized false discovery rates across diverse spatial configurations and expression patterns. Applied to human breast and lung cancer datasets, SpatioCAD demonstrates strong ranking performance, expression-balanced selection, and spatial coherence. Biologically, SpatioCAD identifies SVGs strongly associated with tumor aggressiveness, immune infiltration, and patient prognosis. Furthermore, when applied to a diffuse midline glioma (DMG) sample, SpatioCAD enables the precise delineation of key histological regions, including the tumor core, invasive margin, and peritumoral reactive zone. These results collectively establish SpatioCAD as a powerful and robust tool for deciphering the spatial architecture of complex tissues and offering novel insights into their underlying biology.

## Results

### SpatioCAD overview

SpatioCAD is a computational framework designed for the expression-balanced and efficient identification of SVGs within highly heterogeneous tissues, such as the tumor microenvironment. A critical confounder in these contexts is spatial variation in cell density, which, if not properly addressed, frequently leads to false-positive SVG calls. To overcome this challenge, SpatioCAD is built on two fundamental assumptions: first, biologically meaningful spatial patterns manifest as locally smooth signals; and second, structured signals require a longer diffusion time to reach a steady state than random ones.

Leveraging the first assumption, SpatioCAD first employs a noise filtering module to exclude genes dominated by high-frequency variation. Specifically, we propose a metric called Roughness Score to quantify the magnitude of signal variation during the initial phase of a standard graph diffusion process (Fig 1A). The theoretical foundation of this metric is that genes with structured spatial patterns exhibit minimal signal variations during early diffusion due to their local consistency, while noise genes change drastically. This distinction allows SpatioCAD to adaptively filter out uninformative genes, thereby preventing them from confounding downstream analysis.

**Fig 1:**
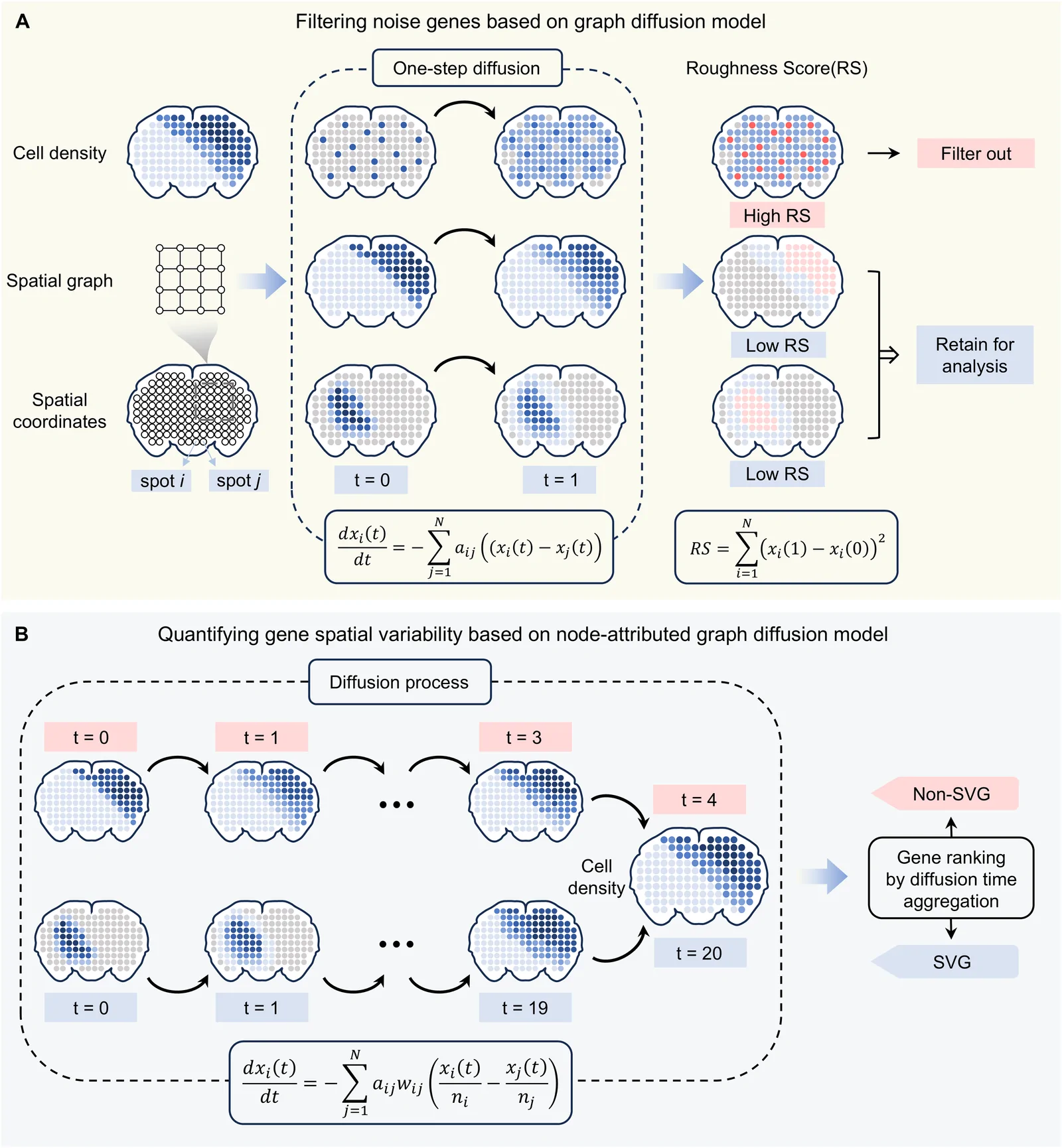
Workflow of SpatioCAD. SpatioCAD is composed of two main modules: noise gene filtering and SVG identification. **A** SpatioCAD first constructs a spatial graph representing the spatial relationships between locations and calculates the cell density for each location using the input spatial transcriptomics data. Then, SpatioCAD filters out noise genes based on the difference between the initial gene expression profile and the profile after a one-step diffusion. **B** SpatioCAD identifies SVGs by computing the diffusion time to the steady state (proportional to cell density) under the Node-Attributed Graph Diffusion (NAGD) model. Genes requiring a longer time to reach the steady state are considered to exhibit significant spatial variation.

Next, SpatioCAD leverages our proposed Node-Attributed Graph Diffusion (NAGD) model to rank the spatial variability of remaining genes based on their characteristic diffusion times (Fig 1B). NAGD generalizes the standard graph diffusion by guiding the diffusion process along per‑cell signal gradients rather than relying on absolute signal differences between spots, thereby decoupling genuine spatial patterns from cellular density variations. Building on the second assumption, we then quantify the spatial variability of each gene by measuring the time required to reach a steady state. A key theoretical foundation of SpatioCAD is that all normalized signals within the NAGD framework converge to a steady state proportional to cell density (Note B in S1 Appendix). This convergence property ensures that the diffusion time serves as a universally comparable metric across genes, enabling a robust and expression-balanced ranking of SVGs.

### Simulation analysis

To systematically benchmark the performance of SpatioCAD against existing methods, we generated datasets simulating the cellular heterogeneity characteristic of tumor tissues (**Methods**). We designed two different domain settings for tissue slices (Fig 2A, first row). Each slice contained five distinct domains, with domain E designated as the tumor core characterized by high cell density. The gene expression counts were generated by sampling from a Zero-Inflated Negative Binomial (ZINB) distribution with different fold change parameters, which were modeled as a product of cell density and spatial variability. The cell density varied between domains and was higher in the tumor core (Fig 2A, second row). The spatial variability for background genes was uniform across domains, whereas for SVGs, it was set to be higher or lower within the specific domains. The resulting final fold change thus captured their compounded effect (Fig 2B). For each simulated tissue architecture, we generated 900 background genes and 200 SVGs, and considered two cases with and without 50 noise genes. We note that although background genes may appear to exhibit spatial patterns, these patterns are driven by cellular heterogeneity and are conceptually distinct from true SVGs (Fig 2C and Fig A in S2 Appendix).

**Fig 2:**
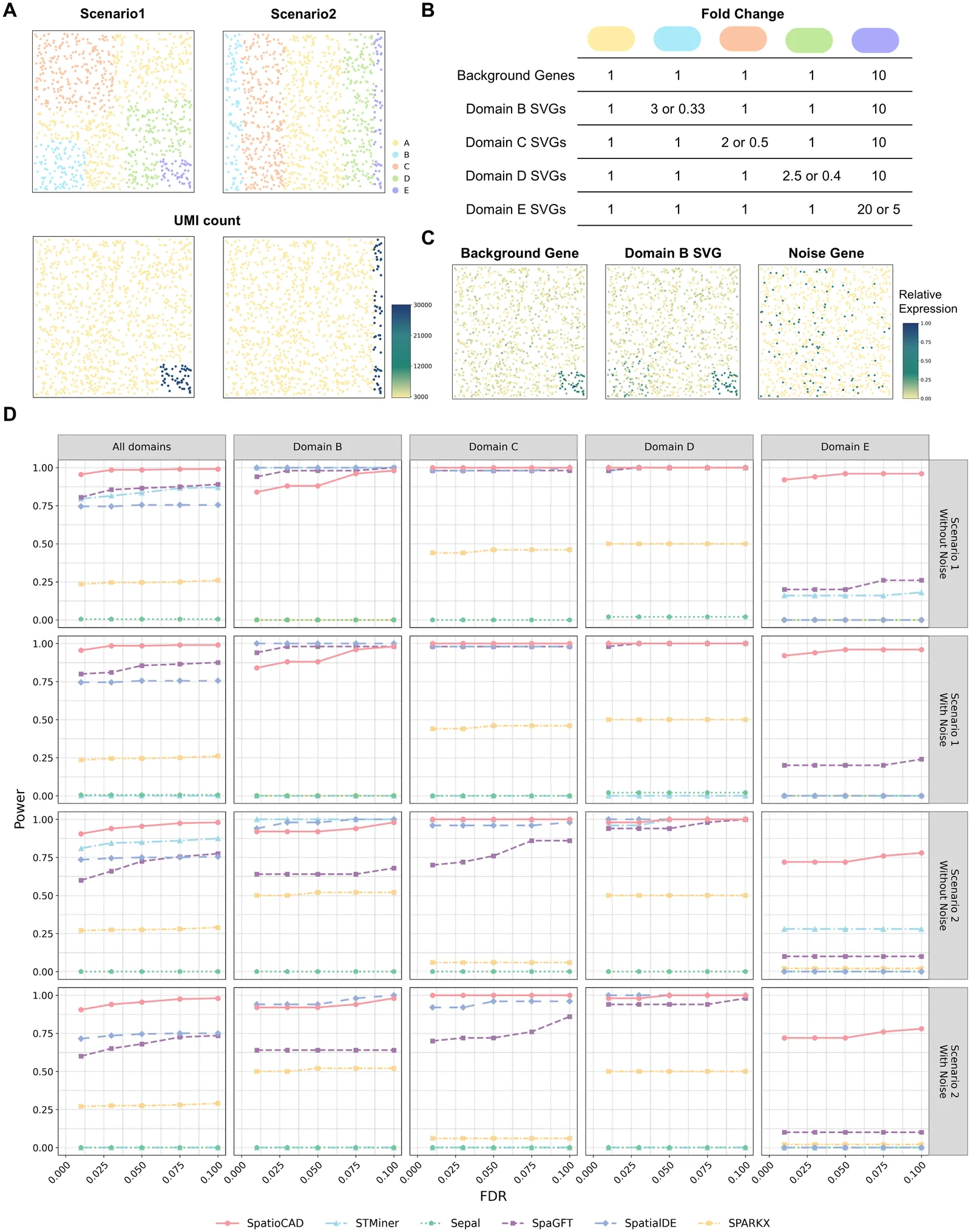
Simulation framework and benchmarking results. **A** Top: two simulated tissue layouts. Scenario 1 with square-shaped domains and Scenario 2 with strip-shaped domains. Colors represent distinct spatial domains. Bottom: spatial distribution of UMI counts corresponding to the two scenarios. **B** Simulation parameter settings of fold-change for different gene categories (**Methods**). **C** Representative spatial expression patterns in Scenario 1 for a background gene, a domain-specific SVG, and a noise gene. **D** Power plots show the statistical power (y-axis) detected by different methods (represented by different colors and line types) at a range of FDR (x-axis) across all domains and the four separate domains (columns) and the two scenarios with and without noise (rows). Results for seed 1 are shown. The complete results for all five seeds are provided in Figs F–I in S2 Appendix.

We benchmarked SpatioCAD against STMiner [19], Sepal [15], SpaGFT [14], SpatialDE [4], and SPARK-X [13]. To account for the diverse output statistics generated by these methods, we evaluated their gene rankings using the known simulation labels. At each predefined false discovery rate (FDR) threshold, we reported the highest empirical power (TPR) attainable among ranked prefixes whose realized FDR did not exceed that threshold (**Methods**).

We first assessed the global performance of each method on simulated datasets without noise genes (Fig 2D, left column). SpatioCAD consistently surpassed all other competing methods, achieving the highest statistical power. While STMiner and SpatialDE demonstrated stability across the two simulation settings, SpaGFT exhibited notable volatility: it performed competitively in Scenario 1 but declined substantially in Scenario 2. SPARK-X showed limited detection capability, while Sepal displayed negligible sensitivity. Specifically, at an FDR threshold of 0.1, SpatioCAD achieved a statistical power of 0.99 (Scenario 1) and 0.98 (Scenario 2), substantially surpassing the second- to fourth-best methods: STMiner (0.87 and 0.88), SpaGFT (0.88 and 0.74), and SpatialDE (0.76 and 0.75). By contrast, the remaining methods yielded powers below 0.30 (SPARK-X: 0.26 and 0.29; Sepal: 0.005 and 0), indicating their limited efficacy under these challenging conditions. The excellent efficacy of SpatioCAD and STMiner is attributable to their specialized designs for complex tissues, as both account for cellular density heterogeneity. And SpatioCAD was more powerful than STMiner by explicitly modeling the diffusion process at the individual cell level, rather than at the location level, thereby achieving finer signal resolution. In comparison, for SpaGFT and SpatialDE, though they normalized cellular expression levels at each location to mitigate density effects, they failed to capture the intrinsic impact of varying cell counts. The performance fluctuations in SpaGFT may stem from the spectral characteristics of graph Fourier transform-based methods, where the capture efficiency for specific spatial patterns depends on their alignment with the graph Laplacian’s eigenvectors. An analysis of SPARK-X revealed that it identified nearly all background genes and SVGs as spatially significant (*p* < 0.05, Fig B in S2 Appendix). This suggests that SPARK-X misinterprets density-driven heterogeneity as true biological signal. Similarly, an examination of Sepal’s gene ranking showed a complete conflation of background genes and SVGs (Fig C in S2 Appendix). This failure likely results from Sepal’s diffusion model assuming a uniform steady state, which fails to account for the confounding effect of cellular density.

We next evaluated the robustness of each method by introducing noise genes into the simulation. While most methods maintained stable performance, STMiner showed a significant performance decline, with its statistical power dropping from approximately 0.87 in noise-free conditions to nearly zero at an FDR threshold of 0.1 upon noise inclusion. Further investigation of the results revealed that STMiner tends to falsely rank noise genes as top candidates (Fig D in S2 Appendix), corroborating the known sensitivity of Optimal Transport-based methods to expression outliers [20,21]. Although stringent quality control in real-world datasets might mitigate this issue, the consistently high performance of SpatioCAD underscores its inherent robustness and reduced reliance on ideal preprocessing.

To assess the contribution of RS-based filtering, we compared the full SpatioCAD pipeline with a variant without this step in both simulation scenarios with noise genes, using five random seeds per scenario (Fig E in S2 Appendix). Removing RS filtering substantially reduced the top-200 F1 score and drove the power at the empirical FDR threshold of 0.05 to zero. The number of noise genes among the top 200 increased from zero in the full pipeline to approximately 50 without RS filtering. These results demonstrate the contribution of the RS module to limiting the prioritization of noise genes and maintaining SVG recovery in these simulations.

We further examined the performance of each method across distinct spatial domains. Given the minimal impact of noise on most methods’ rankings and the limited global performance of SPARK-X and Sepal, we focused our detailed domain-level analysis on the remaining methods under noise-free conditions. Domains B, C, and D represent normal tissue regions with relatively uniform cell density, making SVG detection analogous to standard spatial analysis. In Domains C and D, SpatioCAD, STMiner, and SpatialDE all demonstrated robust performance across two scenarios, achieving near-perfect power (approximately 1.0) across all FDR thresholds (Fig 2D, third and fourth columns from the left). In Domain B, SpatialDE generally achieved higher power than SpatioCAD. To examine whether this difference was specific to the simulation realization shown in Fig 2D, we compared the methods across five random seeds for each scenario, both with and without noise genes (Figs F–I in S2 Appendix). Lower power in Domain B was a recurring tendency for SpatioCAD, although the magnitude and, at some thresholds, the direction of the difference varied across replicates. These results suggest a setting-specific limitation in SpatioCAD’s sensitivity in Domain B, despite its consistently higher power in the all-domain evaluation. Additionally, SpaGFT continued to show scenario-dependent fluctuations across these domains, likely due to the variable alignment between spatial patterns and the graph Laplacian’s eigenvectors.

The most significant performance divergence occurred in Domain E (tumor core), where high cell density creates a strong confounding background. Accurately detecting SVGs in this region is particularly challenging, requiring the capacity to decouple genuine spatial expression from density-driven variation. SpatioCAD was the only method that succeeded in this task (Fig 2D, right column), achieving a power of 0.96 (Scenario 1) and 0.78 (Scenario 2) at an FDR threshold of 0.1, whereas all other methods attained powers below 0.3. This highlights a critical limitation in competing methods: while they may partially adjust for cell density, they fail to disentangle the compounded effects when spatial variability and density variation co‑occur. In contrast, SpatioCAD’s superior performance stems from its NAGD framework, which by directly modeling diffusion at the cellular level, inherently accounts for uneven cell densities and thereby avoids the signal distortion inherent in conventional location‑level normalization.

We further examined the sensitivity of SpatioCAD to the retained eigenmode count, the RS posterior threshold, and the power-transformation set in both noisy simulation scenarios. Across five random seeds per scenario, the reported performance metrics changed little as the eigenmode count varied from 25 to 100 and the posterior threshold from 0.80 to 0.99 (Figs J and K in S2 Appendix). Recovery was more sensitive to the power-transformation set: using only 𝒫={1} yielded lower performance, whereas several alternatives, including the reference set 𝒫={1,1.5,2}, performed well in both scenarios (Fig L in S2 Appendix). Replacing the arithmetic-mean edge weights with geometric or harmonic means also yielded highly similar performance (Fig M in S2 Appendix).

In summary, the simulation analyses not only confirm SpatioCAD as a powerful and robust SVG detection method but also offer a critical benchmarking framework to address the pervasive challenge of cellular heterogeneity in spatial transcriptomics.

### Evaluation of SpatioCAD on human breast cancer data reveals expression-balanced and biologically informative detection of SVGs

We applied SpatioCAD along with the five previously mentioned methods to analyze a public 10X Visium dataset of human breast cancer [23], which comprised 18,085 genes measured across 4,992 spots. Accounting for the diverse output statistics across methods, we focused the comparative analysis on the top 1,000 SVGs identified by each approach to ensure a standardized and equitable assessment. Within the tissue, cancer-associated regions, including invasive regions and DCIS, exhibited higher UMI counts (Fig 3A), aligning well with the established biological prior of increased cell density within tumor regions [24], and highlighting cellularity as a critical confounding factor in spatial transcriptomic analysis.

**Fig 3:**
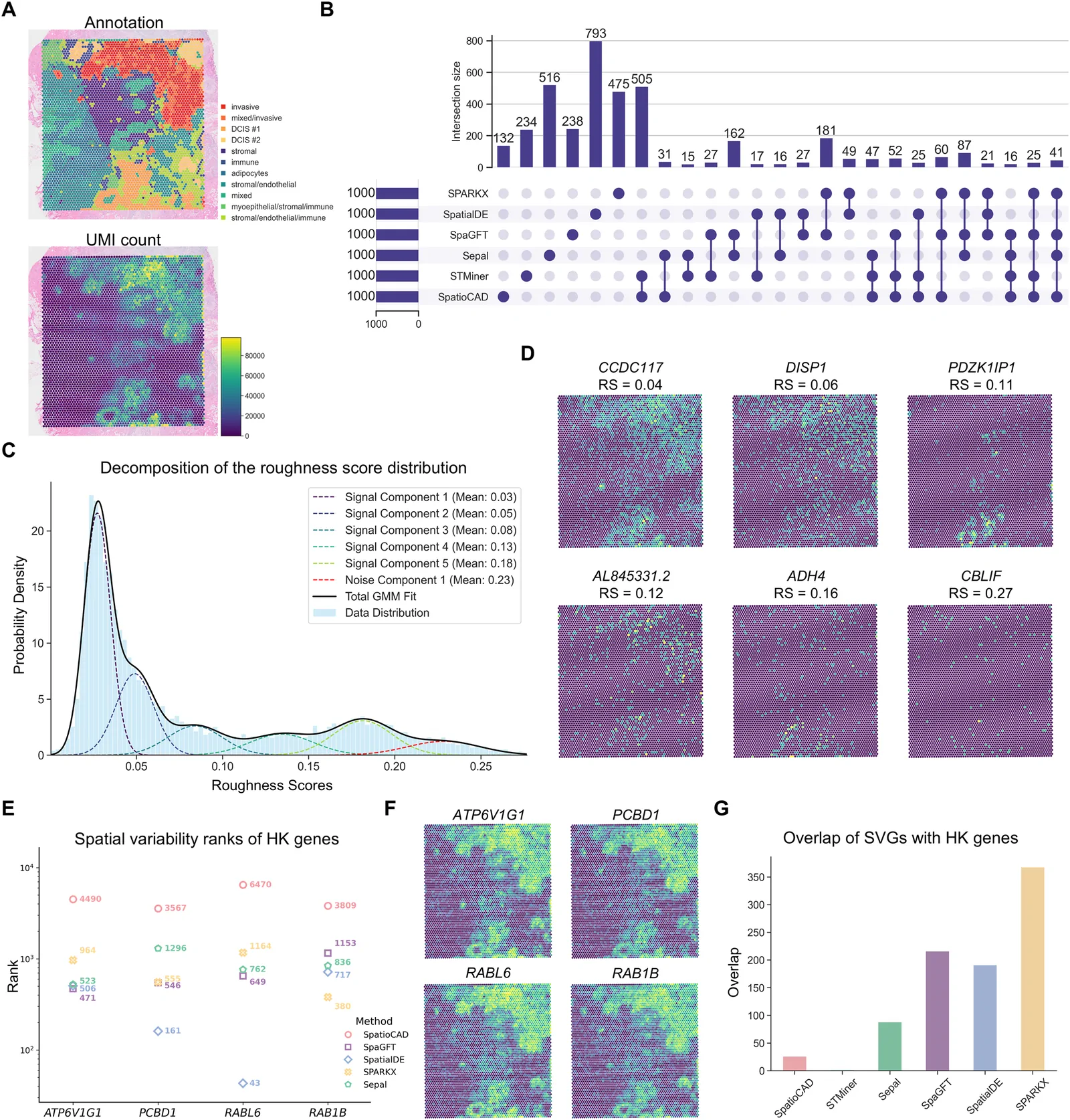
SpatioCAD decouples biological spatial variability from cell density effects in human breast cancer data. **A** Visualization of the human breast cancer dataset with its annotations (upper) and UMI counts (lower). **B** UpSet plot shows the overlap among the top 1,000 SVGs identified by the six compared methods (SpatioCAD, STMiner, Sepal, SpaGFT, SpatialDE, SPARK-X). **C** Decomposition of the Roughness Score (RS) distribution for all genes using a Gaussian Mixture Model, identifying six distinct components in the human breast cancer data. **D** Spatial expression patterns of representative genes from each component with their corresponding RSs. **E** Comparison of spatial variability ranks for representative housekeeping genes when using different methods. **F** Spatial expression patterns of representative housekeeping genes shown in **E**. **G** The number of overlaps between the top 1,000 SVGs identified by different methods and known housekeeping genes in public databases.

#### SpatioCAD effectively decouples biological spatial variability from tissue heterogeneity.

To comprehensively compare the results of the six methods, we generated an UpSet plot depicting the overlaps among their respective top SVGs (Fig 3B), which illustrates the distinct gene preferences of different methods. Notably, SpatioCAD and STMiner shared the largest intersection (505 genes), attributable to their capacity to adjust for cellular heterogeneity. Other pronounced overlaps were also observed, such as between SpaGFT and SPARK-X (181 genes), and between SpaGFT and Sepal (162 genes). Furthermore, SpatioCAD identified the fewest unique SVGs (132 genes), suggesting high detection reliability as most SVGs were corroborated by other methods.

We employed the Roughness Score (RS), derived from standard graph diffusion, to filter irregular expression profiles (**Methods**). The RS distribution was fitted with six Gaussian components, with the component having the highest mean RS designated as the noise component (Fig 3C). This classification was supported by visualization: genes from the highest-RS component (e.g., *CBLIF*) displayed disordered distributions, while genes from lower-RS components (e.g., *ADH4*, *CCDC117*) showed structured patterns (Fig 3D). These results indicate the potential of RS as a metric for noise gene filtering, which is expected to enhance the downstream SVG recovery.

We evaluated the quality of the identified SVGs by using housekeeping (HK) genes as negative controls [25], as they are expected to lack spatial variability. Examination of several HK genes with markedly different rankings between methods revealed that many were spuriously identified as top SVGs by conventional approaches, whereas SpatioCAD correctly excluded them by ranking them beyond the top 1000 (Fig 3E). As shown in Fig 3F, the spatial expression of these genes strongly correlated with UMI counts, confirming their density-dependent nature. Quantitatively, the overlap between the SVGs and HK genes was lowest for STMiner (2 genes) and comparably low for SpatioCAD (26 genes). Both methods significantly outperformed the others, which showed substantially higher overlaps: Sepal (88 genes), SpaGFT (216 genes), SpatialDE (191 genes), and SPARK-X (368 genes) (Fig 3G). This finding emphasizes the importance of accounting for cell density for capturing genuine spatial variation in heterogeneous tissues.

#### SpatioCAD identifies expression-balanced and functionally diverse SVGs.

Given the substantial divergence in SVG lists, we investigated whether the top SVGs identified by each method were systematically skewed toward higher or lower expression by calculating their normalized expression ranks relative to all genes (**Methods**). We used 0.5 as the reference mean normalized expression rank under equal-probability gene selection (**Methods**). Among the evaluated methods, the genes selected by SpatioCAD had the mean rank closest to this reference (mean: 0.487; mean absolute deviation (MAD): 0.013; Fig 4A). In contrast, STMiner showed a preference for lowly expressed genes (mean: 0.398; MAD: 0.102), whereas SpatialDE (mean: 0.687; MAD: 0.187), Sepal (mean: 0.859; MAD: 0.359), SpaGFT (mean: 0.871; MAD: 0.371), and SPARK-X (mean: 0.904; MAD: 0.404) were heavily biased towards highly expressed genes. Spearman correlation analysis between spatial variability scores and expression levels further supported these findings (**Methods**): SpatioCAD displayed negligible correlation (R=−0.04), while STMiner displayed a significant negative correlation (R=−0.58). All others displayed significant positive correlations, ranging from SpatialDE (*R* = 0.71) to SpaGFT (*R* = 0.83), SPARK-X (*R* = 0.90), and Sepal (*R* = 0.92). Visual inspection of rank scatter plots corroborated these metrics: SVGs identified by SpatioCAD were uniformly distributed across expression levels, while the SVGs from STMiner clustered along anti-diagonal and those from the remaining methods clustered along diagonal patterns, indicating systematic bias toward either low or high expression (Fig 4B and Fig N in S2 Appendix). These results support more expression-balanced SVG selection by SpatioCAD.

**Fig 4:**
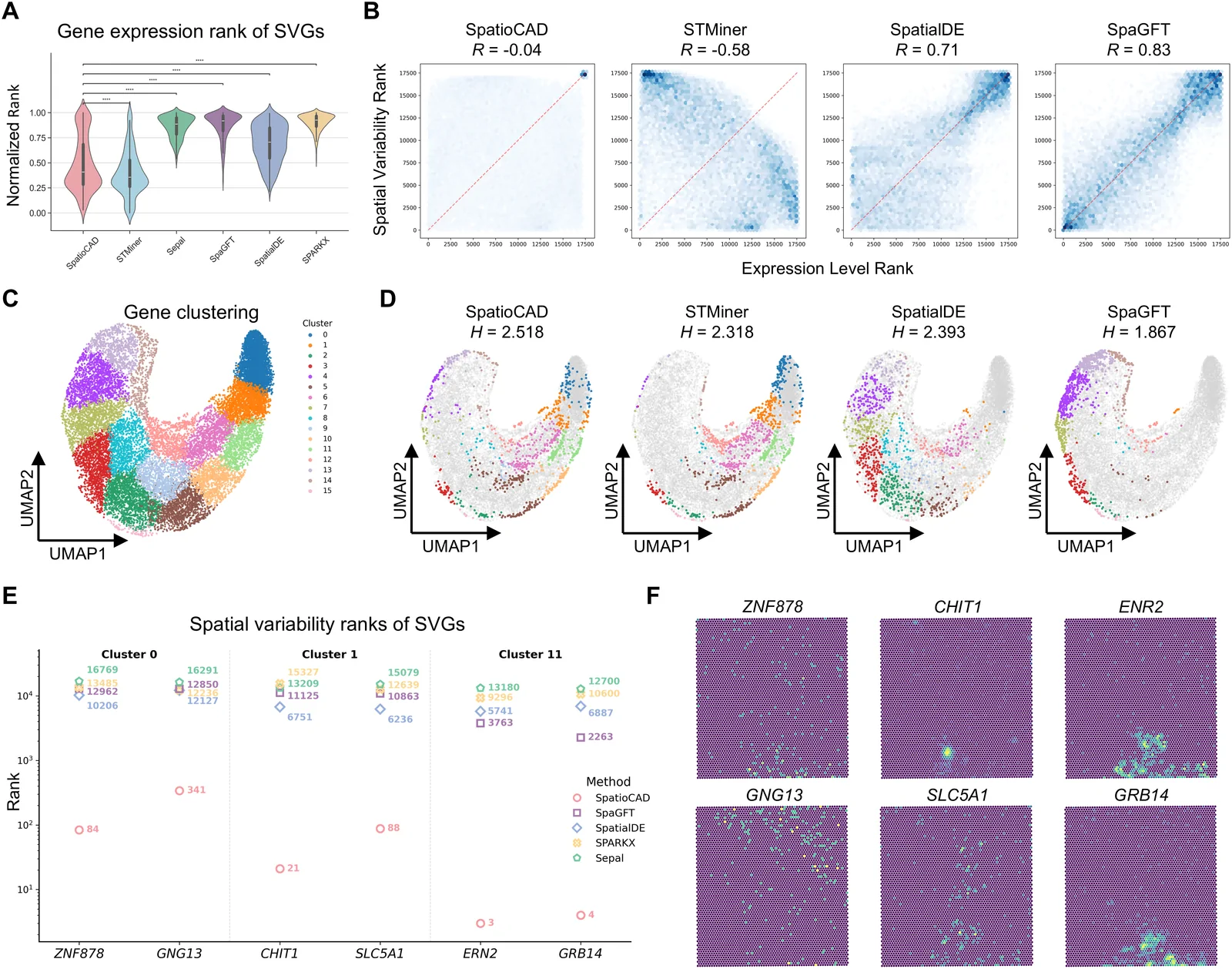
SpatioCAD identifies functionally diverse SVGs with expression balance in human breast cancer data. **A** Violin plot shows the distribution of normalized expression ranks (y-axis) for the top 1,000 SVGs identified by each method. Wilcoxon rank-sum tests were applied to normalized expression ranks comparing SpatioCAD with other methods (*p*-values $=3.37\times 10^{-10}$, $3.30\times 10^{-162}$, $3.66\times 10^{-170}$, $2.03\times 10^{-67}$, $6.94\times 10^{-190}$, respectively). **B** Hexbin density plots show the relationship between gene expression ranks (x-axis) and spatial variability ranks (y-axis). Spearman correlation between the two ranks was calculated for each method (SpatioCAD: −0.04, STMiner: −0.58, SpatialDE: 0.71, SpaGFT: 0.83, SPARK-X: 0.90, Sepal: 0.92). **C** UMAP plot shows the clustering results of all genes based on their expression profiles. **D** Projection of the top 1,000 SVGs identified by each method onto the global gene UMAP. Shannon entropy of their distribution across these clusters was calculated for each method (SpatioCAD: 2.518, STMiner: 2.318, SpatialDE: 2.393, SpaGFT: 1.867, SPARK-X: 1.449, Sepal: 2.013) with higher value indicating more functionally diverse SVGs. **E** Comparison of spatial variability ranks for representative genes from clusters 0, 1, and 11. SpatioCAD ranks these genes higher than other methods (SpatialDE, SpaGFT, SPARK-X, Sepal). **F** Spatial expression patterns of representative genes shown in **E**.

We next evaluated the functional diversity of the SVGs identified by each method. All genes were first grouped into distinct clusters based on their expression profiles (Fig 4C). Gene set enrichment analysis using the Gene Ontology (GO) database confirmed the biological relevance of each cluster (Figs O-Q in S2 Appendix), and the cluster size and expression levels were quantified (Figs R and S in S2 Appendix). We then calculated the Shannon entropy of the SVG distribution across these clusters (**Methods**). A method that captures functionally diverse SVGs should distribute them broadly across clusters, resulting in higher entropy. SpatioCAD achieved the highest entropy (*H* = 2.518), followed by SpatialDE (*H* = 2.393), STMiner (*H* = 2.318), Sepal (*H* = 2.013), SpaGFT (*H* = 1.867), and SPARK-X (*H* = 1.449) (Fig 4D and Fig T in S2 Appendix). This indicates that SpatioCAD captures a significantly broader spectrum of biologically functional signals compared to other methods.

A detailed breakdown of SVG counts per cluster further confirmed the distinct biases for each method (Fig U in S2 Appendix). While SpatioCAD successfully detected biological signals from each cluster, SpatialDE, Sepal, SpaGFT, and SPARK-X identified few or no SVGs in clusters 0, 1, and 11, which correspond to the lowest average gene expression levels (Fig R in S2 Appendix). Conversely, STMiner showed limited sensitivity in clusters 4, 7, 8, 9, and 13, consistent with its bias toward lowly expressed genes. These observations align with the earlier correlation analysis between spatial variability and gene expression levels, underscoring the expression-level bias of these methods in detecting spatial patterns.

We further validated the biological relevance of several SVGs from low-expression clusters (Clusters 0, 1, and 11) that were identified by SpatioCAD but missed by other methods. Their relative rankings across methods are shown in Fig 4E. In Cluster 0, *ZNF878* exhibited a spatial distribution pattern matching the DCIS subtype (Figs 4F and 3A). This observation aligns with the biological context of zinc finger proteins, a family increasingly recognized for their potential roles in cancer progression [26]. *GNG13* exhibited distinct spatial clustering in the invasive regions (Fig 4F). As a member of the G protein γ subunit family, it acts as a critical signal transducer for chemokine receptors [27]. This enrichment at the invasive regions likely reflects the active recruitment of migratory machinery in these disseminating tumor cells. In Cluster 1, *CHIT1* exhibited a highly focal expression pattern within the DCIS regions (Fig 4F). Since *CHIT1* is a biochemical marker for activated macrophages [28], this local region likely reflects tumor-associated macrophage infiltration. Moreover, *SLC5A1* (*SGLT1*) was broadly enriched in DCIS and invasive regions (Fig 4F), consistent with the high glucose uptake required for the glycolytic metabolism of rapidly proliferating pre-invasive cells [29]. In Cluster 11, *ERN2* and *GRB14* exhibited spatial patterns that closely mirrored the DCIS regions (Fig 4F). This co-localization highlights the complex physiological state of pre-invasive tumor cells. Specifically, the high expression of *ERN2*, an endoplasmic reticulum (ER) stress sensor, reflects the activation of the unfolded protein response (UPR) triggered by the intense biosynthetic load and proteotoxic stress inherent to rapidly growing tumor nests [30]. In parallel, *GRB14* functions as a key signal modulator driving breast cancer cell cycle progression and proliferation [31]. These validations demonstrate that SpatioCAD can reliably identify functionally relevant spatial patterns even among low‑abundance transcripts, uncovering biological signals that are otherwise overlooked by expression‑biased methods.

#### SpatioCAD captures more robust biological structures compared to STMiner.

We performed a direct comparison between SpatioCAD and STMiner, a leading approach that addresses cellularity, and focused on the SVGs uniquely identified by each method. After removing common SVGs, both methods yielded an equal number of exclusive candidates. We first assessed spatial coherence of the SVGs using a local linear regression model that predicts gene expression at a specific location based on its neighboring coordinates, to quantify local smoothness, a characteristic of genuine biological signals (**Methods**). The SVGs identified by SpatioCAD exhibited a significantly lower prediction error compared to those from STMiner (MAE: 3.15 vs 4.95, Wilcoxon-rank test *p*-value = $8.00\times 10^{-26}$; Fig 5A). This result indicates that SpatioCAD detects spatial patterns with high spatial continuity, effectively filtering out the spatially incoherent noise.

**Fig 5:**
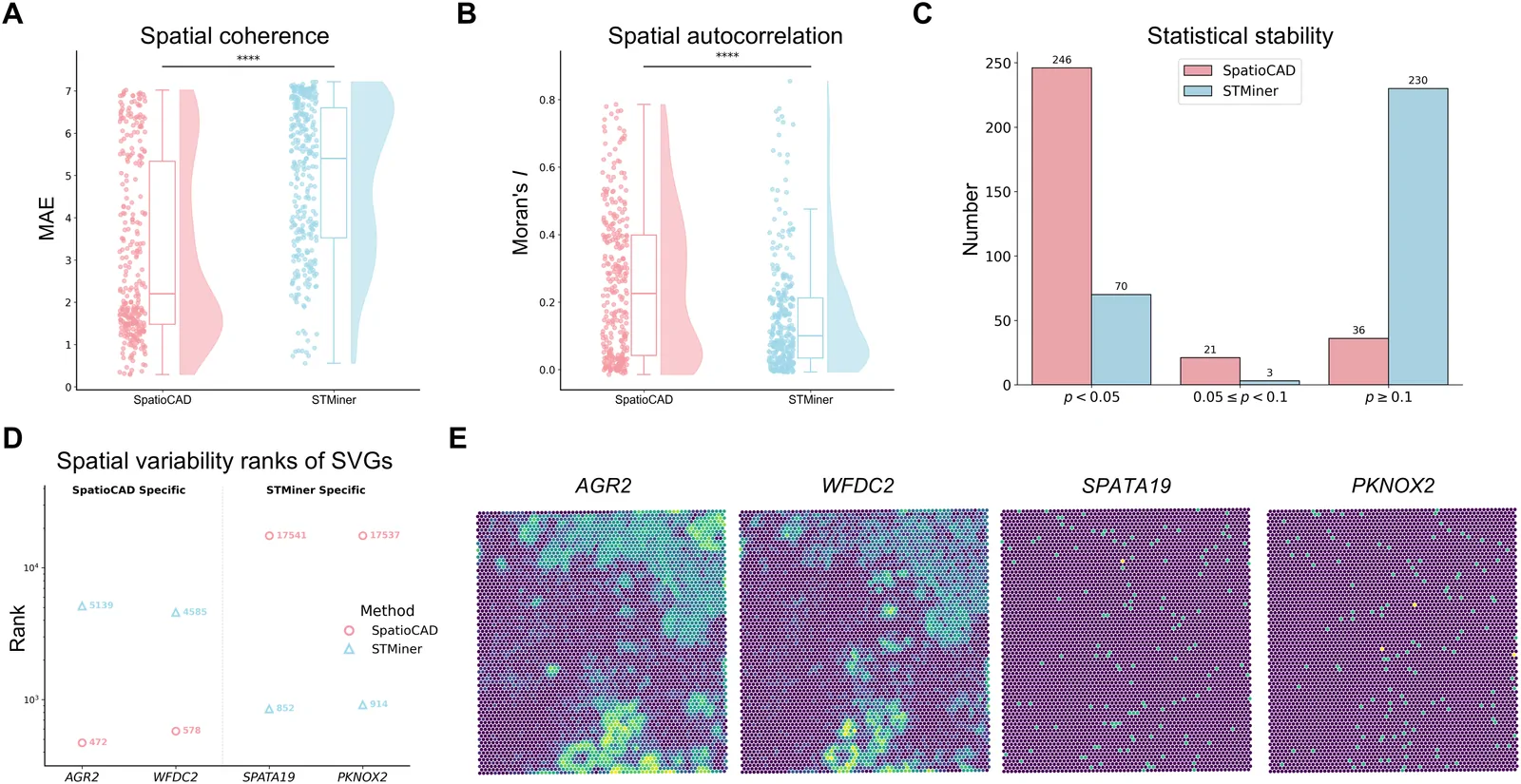
Comparison between SpatioCAD and STMiner for SVG identification in human breast cancer data. **A** Raincloud plots show the distribution of the gene expression reconstruction error (MAE) for method-specific SVGs. Wilcoxon rank-sum test comparing SpatioCAD with STMiner (*p*-value $=8.00\times 10^{-26}$). **B** Raincloud plot shows the distribution of global Moran’s *I* indices for method-specific SVGs. Wilcoxon rank-sum test comparing SpatioCAD with STMiner (*p*-value $=1.72\times 10^{-7}$). **C** Bar plot shows the classification of method-specific SVGs based on permutation-based empirical *p*-values. Empirical *p*-values were estimated using 50 permutations per gene, with a resolution of 0.02. **D** Comparison of spatial variability ranks for representative method-specific SVGs. **E** Spatial expression patterns of representative genes shown in **D**.

We then evaluated the global spatial structure of the identified SVGs using Moran’s *I*, a standard measure of spatial autocorrelation [32]. A higher Moran’s *I* indicates stronger spatial clustering of expression, as opposed to a random or dispersed distribution. SVGs from SpatioCAD consistently displayed significantly higher Moran’s *I* indices (mean: 0.25 vs 0.15, Wilcoxon-rank test *p*-value = $1.72\times 10^{-7}$; Fig 5B). This result confirms that SpatioCAD captures genes with structured spatial patterns, whereas STMiner may select genes with weak or spatially fragmented distributions.

We further assessed the statistical stability of SVG detection using permutation-based test (**Methods**) to distinguish true biological variability from random background fluctuations. SpatioCAD yielded significantly lower empirical *p*-values for its top-ranked SVGs compared to STMiner (Fig 5C). Specifically, 246 SVGs identified by SpatioCAD were statistically significant (*p* < 0.05), whereas only 70 of STMiner’s top SVGs met this threshold. Conversely, a substantial number of genes identified by STMiner (230 genes) exhibited high *p*-values (p≥0.1), suggesting they may be false positives driven by noise. This result suggests that the signals captured by SpatioCAD represent statistically robust biological spatial features rather than stochastic fluctuations.

To visually inspect the discrepancies between the two methods, we examined the spatial expression patterns of genes with significantly different ranks (Fig 5D). SpatioCAD successfully prioritized biologically relevant markers, such as *AGR2* and *WFDC2*. *AGR2* is a well-established estrogen-regulated biomarker and potential therapeutic target in breast cancer [33], while *WFDC2* (*HE4*) has been associated with lymph node metastasis and poor disease-free survival in breast cancer patients [34]. Visual inspection also confirmed that these genes exhibit structured and continuous spatial patterns (Fig 5E, left). In contrast, STMiner prioritized some genes characterized by poor spatial predictability and low statistical confidence, such as *SPATA19* (*p*-value = 0.90) and *PKNOX2* (*p*-value = 0.92), which displayed scattered, discrete noise patterns lacking spatial coherence (Fig 5E, right). Functional analysis also implies that these identifications are likely false positives driven by technical noise. For instance, *SPATA19* is known to be associated with spermatogenesis [35], which is biologically irrelevant to breast tissue.

For the computational efficiency, as STMiner relies on computationally intensive Optimal Transport (OT) to model cellularity, SpatioCAD demonstrated a substantial improvement, completing the analysis in 43 seconds compared to 41,983 seconds for STMiner. This acceleration is directly attributable to the analytical solution of diffusion time in the NAGD model, which enables rapid computation without iterative approximations.

Collectively, these quantitative and qualitative assessments demonstrate SpatioCAD’s superior ability to distinguish biologically meaningful spatial signals from technical artifacts.

### SpatioCAD demonstrates expression-balanced and biologically informative detection of SVGs in human lung cancer data

To assess the generalizability of our findings across different tissue architectures, we applied SpatioCAD and the competing methods to analyze a public 10X Visium dataset of human lung cancer, which comprised 18,085 genes measured across 3,858 spots (Fig 6A). To ensure an equitable comparison, we also focused our analysis on the top 1,000 SVGs identified by each method. The observed heterogeneity in UMI counts across spots further reflects the marked variation in cellular density within this tissue.

**Fig 6:**
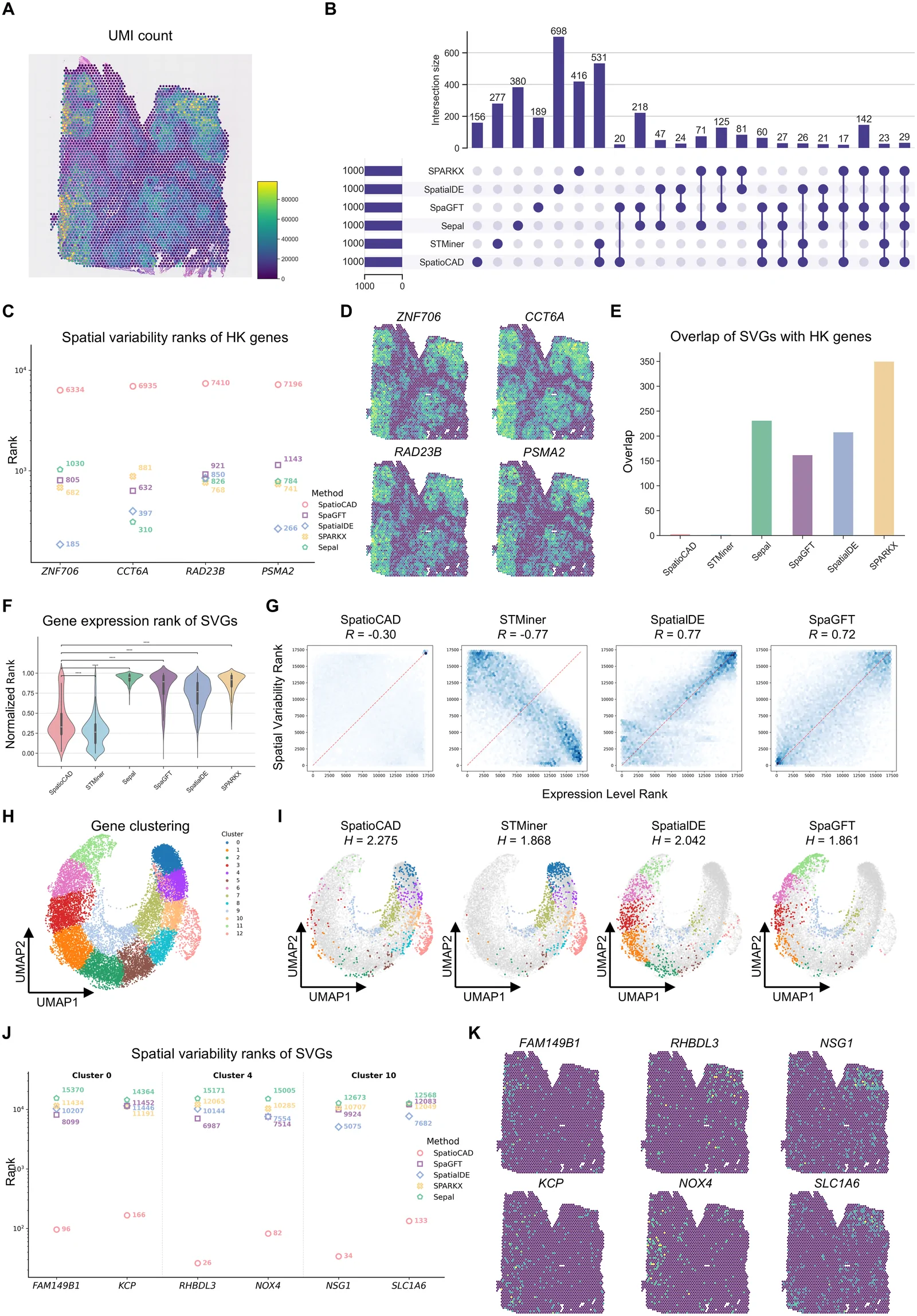
SpatioCAD identifies expression-balanced and functionally diverse SVGs in human lung cancer data. **A** Visualization of the human lung cancer dataset with its UMI counts. **B** UpSet plot shows the overlap among the top 1,000 SVGs identified by the six compared methods (SpatioCAD, STMiner, Sepal, SpaGFT, SpatialDE, SPARK-X). **C** Comparison of spatial variability ranks for representative housekeeping genes. **D** Spatial expression patterns of representative housekeeping genes shown in **C**. **E** The number of overlaps between the top 1,000 SVGs identified by different methods and known housekeeping genes in public databases. **F** Violin plot shows the distribution of normalized expression ranks for the top 1,000 SVGs identified by each method. Wilcoxon rank-sum tests were applied to normalized expression ranks comparing SpatioCAD with other methods (*p*-values $=5.55\times 10^{-29}$, $2.23\times 10^{-266}$, $1.29\times 10^{-202}$, $9.81\times 10^{-161}$, $1.87\times 10^{-239}$, respectively). **G** Hexbin density plots show the relationship between gene expression ranks (x-axis) and spatial variability ranks (y-axis). Spearman correlation between the two ranks was calculated for each method (SpatioCAD: −0.30, STMiner: −0.77, SpatialDE: 0.77, SpaGFT: 0.72, SPARK-X: 0.91, Sepal: 0.97). **H** UMAP plot shows the clustering results of all genes based on their expression profiles. **I** Projection of the top 1,000 SVGs identified by each method onto the global gene UMAP. Shannon entropy of their distribution across these clusters was calculated for each method (SpatioCAD: 2.275, STMiner: 1.868, SpatialDE: 2.042, SpaGFT: 1.861, SPARK-X: 1.645, Sepal: 1.203). **J** Comparison of spatial variability ranks for representative genes from clusters 0, 4, and 10. SpatioCAD ranks these genes higher than other methods (SpatialDE, SpaGFT, SPARK-X, Sepal). **K** Spatial expression patterns of representative genes shown in **J**.

#### SpatioCAD effectively corrects for tissue heterogeneity, supports expression-balanced selection and recovers functionally diverse SVGs.

We generated an UpSet plot depicting the overlaps among the respective top SVGs identified by the compared methods (Fig 6B). The largest overlap was observed between SpatioCAD and STMiner (531 genes), attributable to their shared capability to adjust for cellular heterogeneity. SpatioCAD identified the fewest unique SVGs (156 genes), indicating the high reliability as most of its findings were supported by other methods.

Using housekeeping genes as negative controls, we evaluated the specificity of top-ranked SVGs. As shown in the ranking plot (Fig 6C), several HK genes were spuriously ranked as top SVGs by other methods but were correctly excluded by SpatioCAD by ranking them beyond the top 1000. The spatial expression patterns of these representative HK genes further reveal that their apparent spatial variation closely mirrors the UMI count distribution (Fig 6D), confirming they reflect cell density variation rather than genuine biological patterning. Quantitatively, SpatioCAD and STMiner yielded the lowest overlap with only 3 and 2 genes detected, respectively. This stands in stark contrast to other methods, which generally exhibited higher overlaps: Sepal (231 genes), SpaGFT (162 genes), SpatialDE (208 genes), and SPARK-X (350 genes) (Fig 6E). This consistent and robust performance highlights the importance of decoupling cell density to ensure the identification of genuine biological signals in tumors.

Next, we examined whether each method’s top SVGs were systematically biased toward high or low expression by analyzing their normalized expression ranks (**Methods**). SpatioCAD again yielded the mean normalized expression rank closest to the reference value of 0.5 among the evaluated methods (mean: 0.396; MAD: 0.104). In contrast, STMiner favored lowly expressed genes (mean: 0.272; MAD: 0.228), while SpatialDE (mean: 0.735; MAD: 0.235), SpaGFT (mean: 0.826; MAD: 0.326), SPARK-X (mean: 0.883; MAD: 0.383) and Sepal (mean: 0.935; MAD: 0.435) remained heavily skewed towards highly expressed genes (Fig 6F). Spearman correlation analysis further supported these findings: SpatioCAD showed a weaker monotonic association with expression abundance (R=−0.30) than the competing methods, whereas STMiner exhibited a significant negative correlation (R=−0.77), and all other methods showed strong positive correlations, ranging from SpaGFT (*R* = 0.72) and SpatialDE (*R* = 0.77) to SPARK-X (*R* = 0.91) and Sepal (*R* = 0.97). Visualization of rank scatter plots confirmed that SpatioCAD’s SVGs were uniformly distributed across expression levels, while SVGs from other methods clustered along diagonal or anti-diagonal patterns, indicating systematic expression-level bias (Fig 6G and Fig V in S2 Appendix).

To assess the functional diversity of identified SVGs, we leveraged the established expression-based clustering framework (**Methods**, Figs W-Z in S2 Appendix and Fig A in S3 Appendix). Genes were clustered based on expression profiles, and Shannon entropy of SVG distribution across the clusters was calculated (Fig 6H and 6I). SpatioCAD again achieved the highest entropy (*H* = 2.275), followed by SpatialDE (*H* = 2.042), SpaGFT (*H* = 1.861), STMiner (*H* = 1.868), SPARK-X (*H* = 1.645), and Sepal (*H* = 1.203) (Fig 6I and Fig B in S3 Appendix). This result confirms that SpatioCAD’s capacity to recover a comprehensive spectrum of functional signals is not dataset-dependent but a robust feature of the algorithm.

Analysis of SVG distribution across clusters highlighted method-specific preference (Fig C in S3 Appendix). SpatioCAD maintained robust detection of biological signals in all clusters, while SpatialDE, Sepal, SpaGFT, and SPARK-X identified few or no SVGs in low-expression clusters 0, 4, and 10 (Fig Z in S2 Appendix). In contrast, STMiner exhibited reduced sensitivity in clusters 1, 2, 3, 6, and 11, reinforcing its bias toward lowly expressed genes. These findings corroborate the earlier observed dependency between spatial variability and expression abundance for all the methods.

We further investigated the biological significance of SVGs in the low-expression clusters (Clusters 0, 4, and 10) identified by SpatioCAD but missed by other methods (Fig 6J). These SVGs revealed two spatially distinct regions within the lung cancer tissue (Fig 6K). On the left side of the lung cancer tissue, we observed a spatial aggregation of *NOX4*, *KCP*, and *FAM149B1* (Fig 6K). This co-localization pattern likely suggests the presence of a tumor-stroma interface undergoing active remodeling. Specifically, *NOX4* has been confirmed to play an important role in pulmonary tissue fibrogenesis [36]. Conversely, *KCP* acts as an enhancer of bone morphogenetic protein (BMP) signaling, a pathway shown to attenuate pulmonary fibrosis [37]. The presence of *KCP* thus suggests that the tissue within this niche exhibits a counter-regulatory response to the fibrotic process. The top-right region, enriched with *NSG1*, *SLC1A6*, and *RHBDL3*, likely represents the tumor core. Specifically, *NSG1* has been identified as a key gene deeply implicated in the pathology of lung adenocarcinoma, with its expression levels correlating strongly with tumor staging [38]. *SLC1A6* serves as a robust survival predictor, capable of stratifying lung adenocarcinoma patients into high-risk groups with significantly poorer prognosis [39].

#### SpatioCAD captures more robust biological structures than STMiner.

To directly compare methods that address cell density confounding, we conducted a focused analysis between SpatioCAD and STMiner centered on the SVGs uniquely identified by each method. We first quantified the local smoothness of the top SVGs using a local linear regression framework, which predicts the expression level at each spot using its surrounding coordinates (**Methods**). SpatioCAD achieved significantly lower prediction error compared to STMiner (mean error: 5.00 vs 8.26, Wilcoxon-rank test *p*-value = $1.46\times 10^{-57}$; Fig 7A). This result indicates that SpatioCAD is capable of filtering out spatially fragmented signals, ensuring the identification of robust and continuous spatial structures.

**Fig 7:**
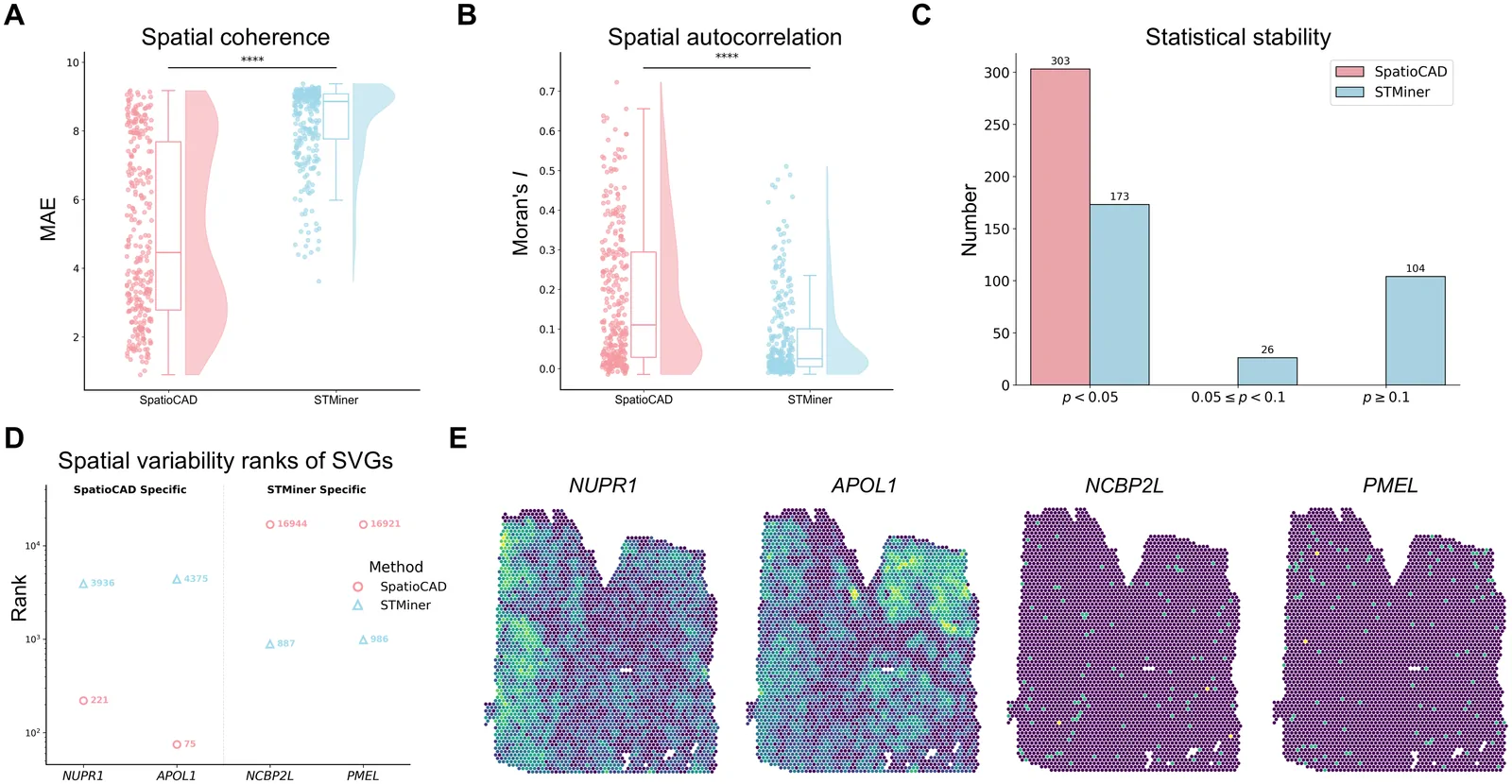
SpatioCAD identifies more spatially coherent and statistically robust SVGs than STMiner in human lung cancer data. **A** Raincloud plot shows the distribution of gene expression reconstruction error for method-specific SVGs. Wilcoxon rank-sum test comparing SpatioCAD with STMiner (*p*-value $=1.46\times 10^{-57}$). **B** Raincloud plot shows the distribution of global Moran’s *I* indices for method-specific SVGs. Wilcoxon rank-sum test comparing SpatioCAD with STMiner (*p*-value $=5.69\times 10^{-18}$). **C** Bar plot shows the classification of method-specific SVGs based on permutation-based empirical *p*-values. Empirical *p*-values were estimated using 50 permutations per gene, with a resolution of 0.02. **D** Comparison of spatial variability ranks for representative method-specific SVGs. **E** Spatial expression patterns of representative genes shown in **D**.

We next employed Moran’s *I* to assess global spatial autocorrelation (**Methods**). The SVGs identified by SpatioCAD exhibited markedly higher Moran’s I indices than those from STMiner (mean: 0.18 vs 0.07, Wilcoxon-rank test *p*-value = $5.69\times 10^{-18}$; Fig 7B). This statistically significant difference confirms that SpatioCAD captures genes with stronger spatial structures, which are more likely to encode rich and interpretable biological information.

We further assessed the statistical stability of identified SVGs using permutation-based testing to evaluate whether their significance persists under random perturbations (**Methods**). SpatioCAD demonstrated exceptional specificity, with all 303 top SVGs exclusive to it achieving statistical significance (*p* < 0.05). In comparison, STMiner yielded a significantly lower number of significant genes (173 genes), with a substantial portion of its top candidates (104 genes) exhibiting high empirical *p*-values (p≥0.1) (Fig 7C). This high proportion of non-significant genes suggests that many of STMiner’s candidates may lack genuine spatial variability and represent false positives driven by stochastic noise. Together, these validations on the lung cancer dataset confirm that SpatioCAD generates statistically robust and biologically coherent results, supporting its reliability across diverse cancer types.

To visually validate these statistical findings, we inspected the expression patterns of several SVGs that were highly ranked by one method but assigned low ranks by another (Fig 7D). SpatioCAD successfully identified key lung cancer-associated markers, such as *NUPR1* and *APOL1*. Specifically, *NUPR1* has been shown to be essential for tumor progression, as its inhibition significantly suppresses the growth of non-small cell lung cancer [40]. Meanwhile, *APOL1* functions as a critical regulator of autophagy and programmed cell death, influencing tumor cell survival under stress [41]. Visualizing these genes reveals clear, spatially aggregated expression structures (Fig 7E, left). Conversely, STMiner prioritized some genes characterized by poor spatial prediction and low statistical confidence, such as *PMEL* (empirical *p*-value: 0.40) and *NCBP2L* (empirical *p*-value: 0.40). From a functional perspective, *PMEL* is a pigment cell-specific protein involved in the biogenesis of pigment organelles [42]. Consequently, its identification as a spatially variable gene in lung tissue is highly indicative of a false positive result. Consistent with this functional discrepancy, the spatial patterns for these genes display scattered, discrete noise patterns (Fig 7E, right), further confirming that SpatioCAD successfully isolates true biological signals from technical artifacts.

Finally, in terms of computational efficiency, SpatioCAD dramatically outperformed STMiner in runtime, completing the analysis in just 40 seconds compared to 23,410 seconds for STMiner.

### SpatioCAD dissects functional heterogeneity in the tumor microenvironment of diffuse midline glioma

One key strength of SpatioCAD is its ability to group SVGs into clusters that share similar spatial expression patterns based on their diffusion profiles, enabling systematic functional dissection of distinct regions within the tumor microenvironment. To demonstrate this capability, we applied SpatioCAD to a publicly available 10X Visium dataset of diffuse midline glioma (DMG). Visualization of three human malignant glioma samples, along with their provided annotations and UMI counts, confirmed the increased cell density in tumor regions (Fig 8A).

**Fig 8:**
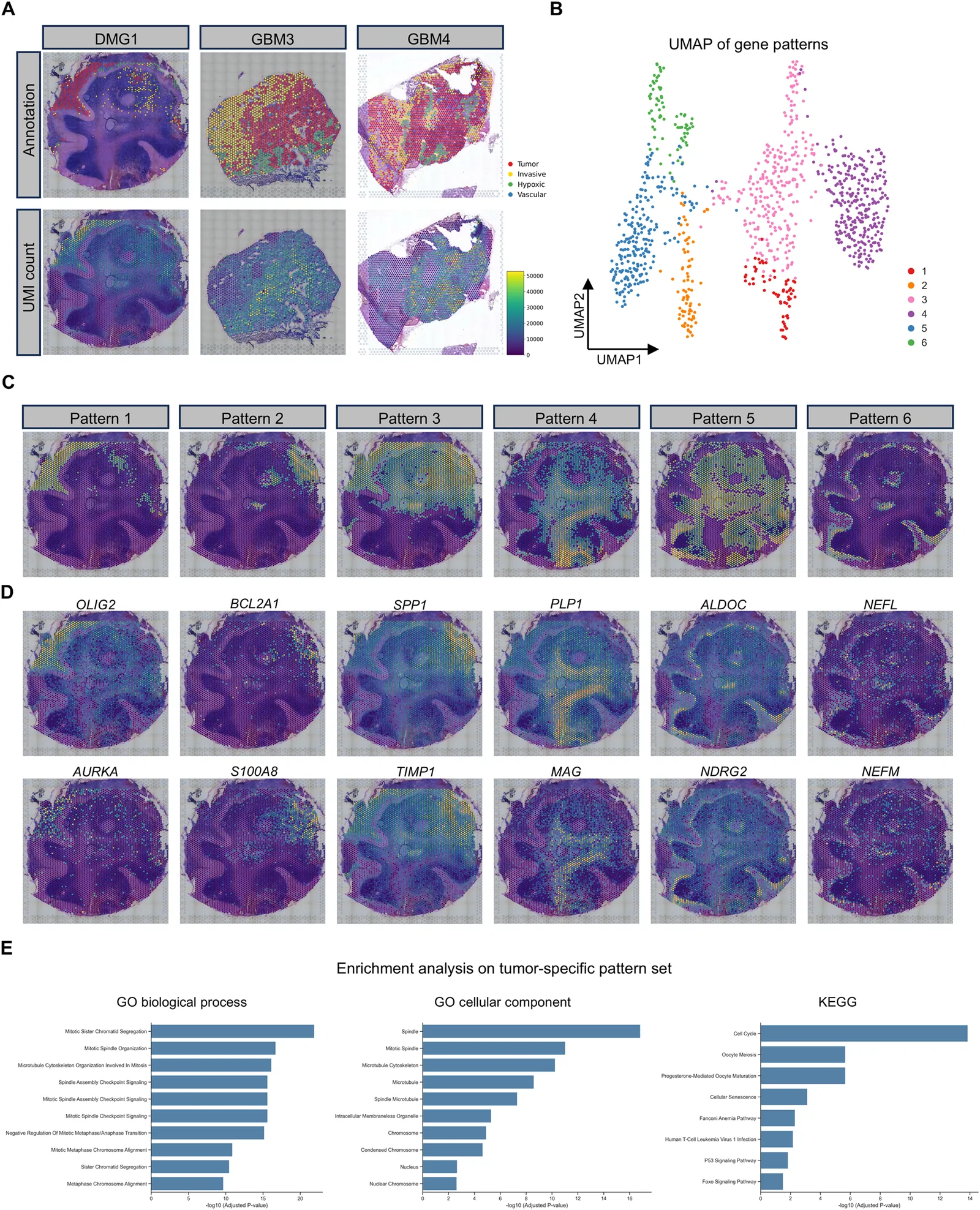
Analysis of human malignant gliomas data. **A** Visualization of three samples from the human malignant gliomas dataset (DMG1, GBM3, and GBM4) with their annotations (upper) and UMI counts (lower). **B** UMAP plot shows the gene clusters based on their spatial distribution characteristics, with genes colored by the cluster labels generated by SpatioCAD. **C** Spatial patterns of the six distinct gene clusters identified by SpatioCAD. **D** Spatial expression patterns of representative genes from each of the six identified clusters. **E** Significantly enriched gene sets (FDR adjusted *p*-value < 0.05) in gene set enrichment results for the tumor-specific gene set (Cluster 1) on three reference gene sets: GO biological process, GO cellular component, and KEGG.

We focused on the top 1,000 SVGs identified by SpatioCAD in sample DMG1, which comprises measurements of 36,601 genes across 4,337 spots. These SVGs were clustered based on their spatial diffusion profiles, which model the diffusion process as a superposition of constituent eigenmodes (**Methods**). To evaluate the biological relevance of these spatial diffusion profiles, we visualized the genes in the UMAP embedding constructed from their normalized expression data, colored by SpatioCAD cluster identities (Fig 8B). The results showed strong concordance: genes belonging to the same SpatioCAD cluster formed compact, distinct groups in expression space, confirming that diffusion profiles effectively capture biologically meaningful spatial patterns. Furthermore, we investigated the spatial patterns of each cluster (Fig 8C). Comparison with the provided annotations revealed that SpatioCAD successfully captured the tumor core, further validating its power in resolving tissue architecture.

We next analyzed the biological functions of the SVGs within each spatial cluster (Fig 8D). Pattern 1, which showed high spatial overlap with the annotated tumor core, was enriched for genes integral to the pathogenesis of DMG. These included *OLIG2*, a marker highly expressed in DMG that is essential for the tumor cell propagation [43], and *AURKA*, whose inhibition has been demonstrated to block DMG cell cycle progression [44]. Pattern 2 was predominantly active in the tumor periphery, indicative of a reactive host response to the encroaching malignancy. This cluster was characterized by key inflammatory genes, including *BCL2A1* and *S100A8* [45,46], a molecular signature characteristic of a robust macrophage response to tumor infiltration [47]. Pattern 3, spanning both the annotated tumor and invasive regions, appeared to represent an active zone of tumor invasion. This observation is supported by previous studies confirming that genes within this cluster, such as *SPP1* and *TIMP1*, are associated with DMG pathogenesis [48,49].

Beyond characterizing the tumor microenvironment, SpatioCAD also successfully recapitulated the fundamental brain tissue architecture (Fig 8D). Pattern 4 exhibited a spatial distribution closely corresponding to the inner eosinophilic (pink) regions observed in the H&E-stained histology. This morphological alignment suggested its identity as white matter, which was biologically validated by the specific enrichment of myelin-sheath-associated genes, such as *PLP1* and *MAG* [50]. Pattern 5 spatially coincided with the hematoxylin-rich (dark purple) regions. We thus identified this region as gray matter, a classification strongly supported by the enrichment of astrocyte-specific markers, including *ALDOC* and *NDRG2* [51,52]. Pattern 6 delineated a distinct, thin layer at the outermost periphery of the tissue. The molecular profile of this pattern was characterized by expression of the neurofilament genes, such as *NEFL* and *NEFM*, indicating the specific capture of a neuronal layer distinct from the inner parenchyma [50,51].

The gene set enrichment analysis on the tumor-specific cluster (Cluster 1) using the Gene Ontology (GO) and KEGG databases is shown in Fig 8E. The results indicated an overwhelming enrichment of genes involved in the regulation and machinery of cell division. Specifically, GO biological process analysis showed strong enrichment in mitotic processes, while GO cellular component analysis revealed that the genes were significantly enriched in components of the spindle and mitotic spindle. This finding is highly consistent with uncontrolled proliferation, a core hallmark of cancer [53]. Corroborating this conclusion, the KEGG pathway analysis also identified ‘Cell Cycle’ as the most significantly enriched pathway. We further extended this functional characterization to the remaining clusters (Clusters 2–6), where the enrichment analyses consistently validated our spatial annotations, spanning from inflammatory responses to specific metabolic and synaptic signatures (Fig D in S3 Appendix).

Finally, we extended our analysis to the other two malignant glioma samples (GBM3 and GBM4). SpatioCAD consistently resolved the heterogeneous tumor architecture and identified the tumor core in both datasets, confirming the method’s robustness across independent samples (Figs E-H in S3 Appendix).

### SpatioCAD identifies expression-balanced and biologically informative SVGs in DLPFC

To further examine whether SpatioCAD remains effective beyond cancer tissues, we applied SpatioCAD and the competing methods to a public 10X Visium dataset of human dorsolateral prefrontal cortex (DLPFC), comprising 12 sections with annotated cortical layers and white matter with the representative slices shown in Fig 9A [54]. Unlike the tumour datasets analysed above, the DLPFC has a well-organized laminar architecture, providing an orthogonal benchmark to evaluate whether each method can recover spatially coherent SVGs in normal tissue contexts. The SVG analyses were performed independently for each section. For each method and section, we selected the top 500 SVGs, and the reported evaluation metrics were obtained by averaging these section-level summaries across the 12 sections.

**Fig 9:**
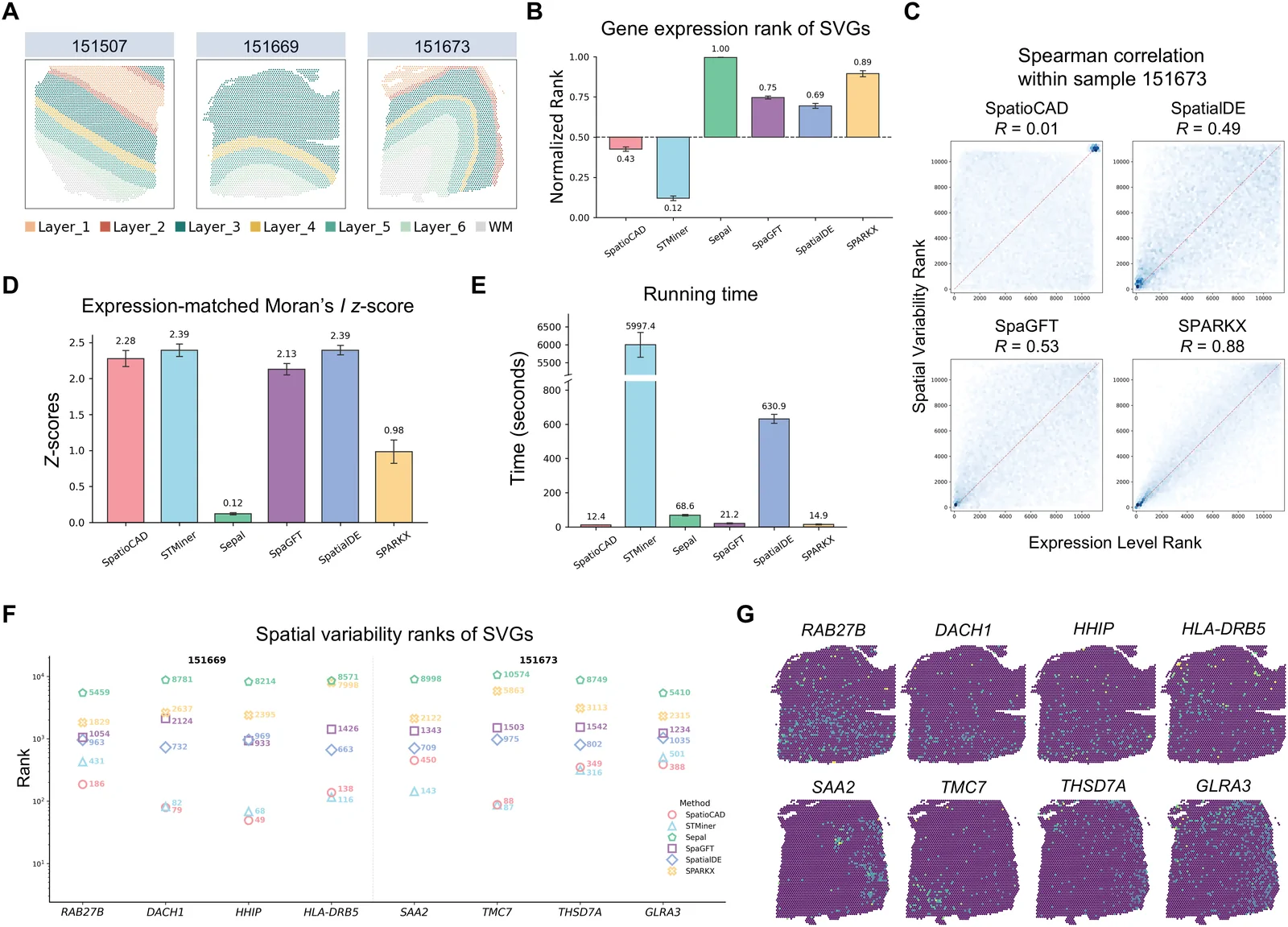
Analysis of human dorsolateral prefrontal cortex data. **A** Visualization of three representative DLPFC sections with their annotations. **B** Bar plot shows the mean normalized expression ranks (y-axis) for the top 500 SVGs identified by each method across 12 DLPFC samples. **C** Hexbin density plots show the relationship between gene expression ranks (x-axis) and spatial variability ranks (y-axis) in sample 151673. Spearman correlation between the two ranks was calculated for each method (SpatioCAD: 0.01, STMiner: −0.60, SpatialDE: 0.49, SpaGFT: 0.53, SPARK-X: 0.88, Sepal: 0.99). **D** Bar plot shows the mean expression-matched Moran’s *I z*-score for the top 500 SVGs identified by each method across 12 DLPFC samples. **E** Running time of each method. **F** Comparison of spatial variability ranks for representative genes in samples 151669 and 151673. **G** Spatial expression patterns of representative genes shown in **F**.

We first examined the expression-rank distribution of the top 500 SVGs identified by each method (**Methods**). The genes selected by SpatioCAD had a mean normalized rank of 0.43, which was closest to the equal-probability selection reference of 0.5 among the evaluated methods. STMiner preferentially selected lowly expressed genes, with a mean normalized rank of 0.12. In contrast, Sepal, SPARK-X, SpaGFT and SpatialDE were more strongly skewed towards highly expressed genes, with mean normalized ranks of 1.00, 0.89, 0.75 and 0.69, respectively (Fig 9B). This pattern was also evident at the single-section level. In sample 151673, the Spearman correlation between expression-level rank and method-specific spatial-variability rank was nearly absent for SpatioCAD (*R* = 0.01), whereas Sepal, SPARK-X, STMiner, SpaGFT and SpatialDE showed stronger correlations with expression abundance (*R* = 0.99, 0.88, −0.60, 0.53 and 0.49, respectively) (Fig 9C and Fig I in S3 Appendix). These results indicate that SpatioCAD rankings were less driven by gene expression level than those of the competing methods.

As spatial autocorrelation can still be influenced by expression abundance and detection frequency, we further calculated the expression-matched Moran’s *I z*-score, which compares each gene with background genes of similar mean expression and detection rate (**Methods**). SpatioCAD achieved a mean expression-matched Moran’s *I z*-score of 2.28, compared with 2.39 for STMiner, 2.39 for SpatialDE, 2.13 for SpaGFT, 0.98 for SPARK-X, and 0.12 for Sepal (Fig 9D). STMiner and SpatialDE therefore also prioritized genes with high expression-matched spatial autocorrelation, although their selected gene sets were skewed toward lower and higher expression levels, respectively. SpatioCAD combined high matched spatial autocorrelation with a mean expression rank closer to the reference value of 0.5. It also retained favorable computational efficiency, with a mean per-section runtime of 12.4 s, compared with 5997.4 s for STMiner, 630.9 s for SpatialDE, 68.6 s for Sepal, 21.2 s for SpaGFT, and 14.9 s for SPARK-X (Fig 9E).

The combination of expression-balanced selection and strong matched spatial autocorrelation suggests that SpatioCAD provides a complementary view of spatial gene regulation. To illustrate this point at the gene level, we examined representative SpatioCAD-prioritized SVGs in two DLPFC sections using the same top-500 criterion. In section 151669, SpatioCAD ranked *RAB27B*, *DACH1*, *HHIP*, and *HLA-DRB5* within the top 500. These four genes were also ranked within the top 500 by STMiner, but outside this cutoff by Sepal, SpaGFT, SpatialDE, and SPARK-X (Fig 9F). In section 151673, SpatioCAD ranked *SAA2*, *TMC7*, *THSD7A*, and *GLRA3* within the top 500. STMiner likewise prioritized *SAA2*, *TMC7*, and *THSD7A* within the top 500, while *GLRA3* was ranked 501st by STMiner and 388th by SpatioCAD. The remaining four methods ranked all four genes outside the top 500 (Fig 9F). These two sets of genes are linked to diverse biological contexts, including presynaptic vesicle trafficking and exocytosis (*RAB27B*) [55] neocortical developmental programmes (*DACH1*) [56], Hedgehog-pathway modulation, a signalling axis involved in cortical development (*HHIP*) [57], MHC class II–associated antigen presentation and neuroimmune interactions (*HLA-DRB5*) [58,59], acute-phase inflammatory response (*SAA2*) [60], Piezo2-associated mechanosensory modulation (*TMC7*) [61], endothelial migration and angiogenic regulation (*THSD7A*) [62], and forebrain glycinergic neurotransmission (*GLRA3*) [63]. Their spatial expression maps revealed gene-specific and section-specific domains rather than a shared high-abundance pattern (Fig 9G), supporting that SpatioCAD can recover biologically interpretable spatial signals that are not simply explained by expression abundance and are partly missed by existing methods. Together with the runtime analysis, these results indicate that SpatioCAD identifies spatially structured and expression-balanced SVGs in normal human cortical tissue while retaining favourable computational efficiency.

## Discussion

In complex tissues such as tumors, varying degrees of cellular aggregation can lead to marked heterogeneity in cell density. This heterogeneity often complicates the analysis of spatial transcriptomics data, resulting in the misidentification of false-positive SVGs. To address this challenge, we developed SpatioCAD, a two-step computational framework. Leveraging the structural instability of noise signals, SpatioCAD first employs the Roughness Score, derived from standard graph diffusion model, to filter out uninformative genes. Subsequently, it calculates the diffusion time for each gene under the Node-Attributed Graph Diffusion (NAGD) model, a generalization of standard graph diffusion, which effectively decouples spatial patterns from cell density variations, thereby enabling the robust identification of genuine SVGs.

The experimental results demonstrate that SpatioCAD effectively mitigates the false positives caused by cellular heterogeneity while yielding more expression-balanced gene selection. This capability facilitates the detection of lowly expressed yet biologically important SVGs, yielding a more diverse and informative SVG set. Furthermore, in a direct comparison with STMiner, an existing method developed for the same purpose, SpatioCAD demonstrates superior efficiency, statistical significance, and robustness. Finally, investigation into the biological functions of the SVGs identified by SpatioCAD reveals its capability to pinpoint clinically significant genes in multiple cancer datasets, confirming its practical relevance.

SpatioCAD is not without limitations. First, unlike many statistical methods, SpatioCAD does not provide a formal statistical measure, such as a *p*-value or *z*-score, for a definitive classification of SVGs and non-SVGs. Second, this framework relies on graph construction and eigen-decomposition of the graph Laplacian matrix. Developing a construction strategy that balances computational efficiency with the preservation of spatial information remains an important direction for future investigation. Lastly, SpatioCAD uses summed normalized expression as an expression-derived relative proxy for cell density rather than a direct cell count. For fixed-area capture locations, total detected expression reflects the number of contributing cells, their average transcript abundance, and capture efficiency. This motivates the use of expression-derived information when variation in cell number is substantial relative to variation in the other factors, but does not imply exact proportionality between the implemented proxy and cell density. Previous studies have documented relationships between cell number and transcript abundance while also identifying contributions from cell type and tissue region [64,65]. Differences in transcriptional activity, RNA quality, tissue permeability, or capture efficiency can therefore affect the proxy. In particular, broad biological changes in transcript abundance may be partly attributed to density by an expression-based adjustment. Histology-derived cellularity would provide valuable independent information for validating or refining this approximation.

In recent years, identifying cell type-specific SVGs (ct-SVGs) has become an active area of research [66–68]. These methods aim to identify genes that exhibit spatial patterns within specific cell types. While SpatioCAD can be adapted to find variations associated with a particular cell type by using its proportion instead of overall cell density in the NAGD framework, it cannot yet resolve the contributions of multiple cell types simultaneously. Extending SpatioCAD to integrate cell-type deconvolution results represents a promising direction for future research.

## Materials and methods

### Context-aware graph diffusion model for pinpointing spatially variable genes in heterogeneous tissues

SpatioCAD is a computational framework designed to identify spatially variable genes (SVGs) within heterogeneous tissues, such as tumors. The core principle of SpatioCAD is to model the spatial diffusion process of gene expression in complex tissues, based on the assumption that genes with structured spatial patterns will reach a steady state more slowly than those with random distributions. The framework consists of two main stages. First, a Roughness Score (RS), derived from the standard graph diffusion model, is calculated to filter out genes with noisy expression patterns, thereby preventing them from confounding downstream analyses. Second, a node-attributed graph diffusion model is proposed to simulate signal propagation across the heterogeneous tumor microenvironment. The spatial variability of each gene is then quantified by the diffusion time required to reach a steady state, with genes exhibiting stronger spatial structure showing longer diffusion durations. Theoretical proofs for this framework are provided in the Notes A-C in S1 Appendix.

#### Spatial graph construction.

Let $X\in {\mathbb{R}}^{N\times G}$ be the preprocessed spatial transcriptomics data matrix, where *N* is the number of spatial locations and *G* is the number of genes. The spatial coordinates of the locations are denoted by $s\in {\mathbb{R}}^{N\times D}$, with the dimensionality *D* = 2 or 3. First, SpatioCAD constructs an undirected graph G=(V,E), where the nodes *V* represent the *N* locations and the edges *E* are established using the mutual *k*-nearest neighbor principle. Specifically, an edge $e_{ij}$ exists between nodes *i* and *j* if and only if $i\in {𝒩}_{k}(j)$ and $j\in {𝒩}_{k}(i)$, where ${𝒩}_{k}(j)$ denotes the set of *k*-nearest neighbors of node *j*. A symmetric adjacency matrix $A=(a_{ij})\in {\mathbb{R}}^{N\times N}$ is then constructed, where the edge weights are calculated using a self-tuning adaptive Gaussian kernel [69]:


$$\begin{array}{c} a_{ij}=\left\{ \begin{array}{cc} \exp\left(-\frac{\|s_{i}-s_{j}{\|}_{2}^{2}}{\sigma_{i}\sigma_{j}}\right) & \text{if }e_{ij}\in E,\\ 0 & \text{otherwise}, \end{array}\right. \end{array}$$ (1)

where $\|\cdot {\|}_{2}$ denotes the Euclidean distance. The local scale parameter $\sigma_{i}$ adapts to the local neighborhood density and is defined as the Euclidean distance from location *i* to its $k^{\prime }$-th nearest neighbor.

Based on simulation experiments (Fig J in S3 Appendix), the default values of *k* and $k^{\prime }$ were set to $\sqrt{N}$ and 0.5logN, respectively, to balance performance and computational efficiency. The constructed graph is checked for connectivity before further analysis, disconnected inputs are flagged and are not processed by the current implementation.

#### Noise gene filtering.

To enhance the statistical power and biological relevance of SVG identification, we implement a filtering strategy based on the standard graph diffusion model to exclude genes dominated by noise. Here, each gene’s expression profile is modeled as a graph signal on a constructed spatial graph, where nodes correspond to distinct spatial locations. We denote the expression level of any gene as $x\in {\mathbb{R}}^{N\times 1}$, with $x_{i}$ being the expression value at spot *i*. The diffusion process of these signals is governed by the graph heat equation [70,71]:


$$\begin{array}{c} \frac{dx_{i}(t)}{dt}=-\sum_{j=1}^{N}a_{ij}\left(x_{i}(t)-x_{j}(t)\right), \end{array}$$ (2)

where $x_{i}(t)$ denotes the signal state of location *i* at time *t*, with the initial s*t*ate x(0) representing the observed gene expression in X. We can express this in vector form:


$$\begin{array}{c} \frac{dx(t)}{dt}=-Lx(t). \end{array}$$ (3)

Here, L is the graph Laplacian matrix defined as L=D−A, where A is the previously defined adjacency matrix and D is the diagonal degree matrix with entries $d_{ii}=\sum_{j}a_{ij}$. This differential equation has a well-defined analytical solution:


$$\begin{array}{c} x(t)=e^{-tL}x(0), \end{array}$$ (4)

which precisely describes the state of the gene signal at any given time *t*.

Biologically meaningful patterns are typically characterized by local consistency, while noise signals tend to change drastically during the initial diffusion steps. Thus, we define a Roughness Score (RS) to filter the noise genes. This score is formally derived from the *L*_2_-norm of the initial signal variation:


$$\begin{array}{c} \text{Roughness Score}=\frac{\|x(t)-\text{x}(0){\|}_{2}}{t}\approx \frac{\|(I-tL)x(0)-x(0){\|}_{2}}{t}=\|Lx(0){\|}_{2}, \end{array}$$ (5)

where x(0) and x(t) denote the initial and time-*t* state signal vectors, respectively. The approximation is obtained using a first-order Taylor expansion of Eq (4). In prac*t*ice, we may directly set *t* = 1 and use $\|x(t)-\text{x}(0){\|}_{2}$ or $\|x(t)-\text{x}(0){\|}_{2}^{2}$ as the RS. Expression concentrated at isolated high-expression spots can produce sharp contras*t*s with neighboring low-expression spots, resulting in larger changes during early diffusion and hence higher RS. RS-based filtering uses these local variations to screen irregular expression profiles before NAGD-based ranking. Notably, from the perspective of spectral graph theory, a higher RS indicates that the signal is dominated by high-frequency components on the graph (Note A in S1 Appendix).

To establish an adaptive filtering threshold, we model the distribution of the Roughness Scores across all genes using a Gaussian Mixture Model (GMM). We fit GMMs with varying component numbers (by default, from 1 to 10) and select the optimal model based on the Bayesian Information Criterion (BIC) [72]. The Gaussian component with the highest mean RS is then identified as the noise component. Finally, we calculate the posterior probability of belonging to this noise component for each gene. Genes with a posterior probability exceeding a pre-defined threshold (by default, 0.9) are classified as lacking sufficient spatial information and are subsequently excluded from further analysis.

#### Gene spatial variability quantification.

Complex biological tissues, such as tumor tissues, exhibit significant heterogeneity in cellular composition. However, standard graph diffusion models assume uniform node properties, limiting their applicability in heterogeneous contexts. Thus, we propose a Node-Attributed Graph Diffusion (NAGD) model that explicitly incorporates node-specific attributes. The NAGD model extends the conventional diffusion framework by integrating the following key biological principles. First, consistent with canonical models, diffusion between nodes is affected by their spatial connectivity, as encoded in the adjacency matrix A. Second, to account for tissue heterogeneity, we assume the diffusion process is driven by the difference in signal concentration ($x_{i}/n_{i}$ for node *i*) rather than raw signal intensity. This concentration difference between paired nodes is represented by $\left(x_{i}/n_{i}-x_{j}/n_{j}\right)$. Finally, the diffusion process is further constrained by the number of potential communication channels between nodes, denoted as $w_{ij}$, which is related to the cellular populations of the two interacting nodes.

Integrating these principles yields the following equation:


$$\begin{array}{c} \frac{dx_{i}(t)}{dt}=-\sum_{j=1}^{N}a_{ij}w_{ij}\left(\frac{x_{i}(t)}{n_{i}}-\frac{x_{j}(t)}{n_{j}}\right), \end{array}$$ (6)

where $n_{i}$ denotes the cell density of node *i*, which we approximate as the sum of normalized expression across all genes at node *i*. We use the arithmetic mean $w_{ij}=(n_{i}+n_{j})/2$ as a simple symmetric and positive coupling for positive node densities, without introducing an additional tuning parameter. Notably, our model represents a generalization of the standard graph diffusion model, to which it reduces when cell numbers across all nodes are uniform. We compared this definition with the geometric mean, $w_{ij}=\sqrt{n_{i}n_{j}}$, and the harmonic mean, $w_{ij}=2n_{i}n_{j}/(n_{i}+n_{j})$, while keeping the density proxy and its normalization unchanged. These alternatives yielded highly similar performance in the evaluated simulation settings (Fig M in S2 Appendix).

By setting ${\hat{x}}_{i}(t)=x_{i}(t)/\sqrt{n_{i}}$, Eq (6) can be rewritten as:


$$\begin{array}{c} \frac{d{\hat{x}}_{i}(t)}{dt}=-\sum_{j=1}^{N}a_{ij}w_{ij}\left(\frac{{\hat{x}}_{i}(t)}{n_{i}}-\frac{{\hat{x}}_{j}(t)}{\sqrt{n_{i}n_{j}}}\right), \end{array}$$ (7)

which can be expressed in vector form as:


$$\begin{array}{c} \frac{d\hat{x}(t)}{dt}=-L_{\text{ND}}\hat{x}(t), \end{array}$$ (8)

with $L_{\text{ND}}$ defined as:


$$\begin{array}{c} L_{\text{ND}}=N^{-1}D_{A⊙W}-N^{-1/2}(A⊙W)N^{-1/2}. \end{array}$$ (9)

In this expression, ⊙ denotes the Hadamard (element-wise) product, $D_{A⊙W}$ is the degree matrix derived from the element-wise product of A and W, and $N=\text{diag}(n_{1},n_{2},...,n_{N})$ is the diagonal matrix of the cell density vector. Analogous to Eq (4), this linear equation has the analytical solution:


$$\begin{array}{c} \hat{x}(t)=e^{-tL_{\text{ND}}}\hat{x}(0). \end{array}$$ (10)

To further simplify the analytical solution, we perform a spectral decomposition of the NAGD Laplacian matrix $L_{\text{ND}}$:


$$\begin{array}{c} L_{\text{ND}}=V\Lambda V^{⊤},\;\Lambda =\text{diag}(\lambda_{1},\lambda_{2},...,\lambda_{N}),\;V=(v_{1},v_{2},...,v_{N}). \end{array}$$ (11)

Here, for a connected graph, $0=\lambda_{1}<\lambda_{2}\leq ...\leq \lambda_{N}$ are the eigenvalues of $L_{\text{ND}}$, and V is the orthogonal matrix whose columns $v_{k}$ are the corresponding eigenvectors, with the first eigenvector $v_{1}$ corresponding to the steady state (Note B in S1 Appendix).

By substituting this spectral decomposition into Eq (10), the diffusion process can be expressed as a sum of its constituent eigenmodes:


$$\begin{array}{c} \hat{x}(t)=\sum_{k=1}^{N}e^{-t\lambda_{k}}(v_{k}^{⊤}\hat{x}(0))v_{k}. \end{array}$$ (12)

As t→∞, the system converges to its steady state, which is determined by the eigenvector corresponding to the single zero eigenvalue ($\lambda_{1}=0$):


$$\begin{array}{c} \lim_{t\to \infty }\hat{x}(t)=(v_{1}^{⊤}\hat{x}(0))v_{1}. \end{array}$$ (13)

For a connected graph, provided all genes are first normalized to uniform total expression, their diffusion processes will converge to an identical steady state, underpinned by the following two properties. First, for a symmetric matrix $W=(w_{ij})$, the total signal sum, $\sum_{i=1}^{N}x_{i}(t)$, is conserved throughout the diffusion process (Note B in S1 Appendix). Second, the system evolves to a unique steady state $x^{*}$, which is directly proportional to the cell density vector: $x^{*}=c\cdot n$, where $n=(n_{1},n_{2},...,n_{N}{)}^{⊤}$ and *c* is a scalar constant (Note B in S1 Appendix). This provides a common endpoint, making their rates of convergence directly comparable. The connectivity assumption is essential to this interpretation. On a disconnected graph, signal mass is conserved separately within each connected component, and the proportionality constant in the steady state can differ between components. Consequently, a signal can be stationary within every component while retaining expression contrasts between components. Such contrasts are not captured by diffusion decay (Note C in S1 Appendix). We therefore introduce a characteristic diffusion time as a robust metric to quantify the spatial variability of each gene.

We formally define the characteristic diffusion time, $t^{*}$, as the time required for the change rate norm of the diffusion state vector, $\left\|d\hat{x}(t)/dt\right\|$, to fall below a predefined threshold ϵ:


$$\begin{array}{c} t^{*}=\inf\left\{t\geq 0|\left\|\sum_{k=2}^{N}-\lambda_{k}e^{-t\lambda_{k}}(v_{k}^{⊤}\hat{x}(0))v_{k}\right\|\leq \epsilon \right\}. \end{array}$$ (14)

Given the orthogonality of the eigenvectors, this expression simplifies under the *L*_2_-norm to:


$$\begin{array}{c} t^{*}=\inf\left\{t\geq 0|\sum_{k=2}^{N}{\left(\lambda_{k}e^{-t\lambda_{k}}(v_{k}^{⊤}\hat{x}(0))\right)}^{2}\leq \epsilon \right\}. \end{array}$$ (15)

In practice, the convergence rate is governed by the slowest-decaying terms, which correspond to the smallest non-zero eigenvalues. To obtain a tractable analytical expression, we approximate $t^{*}$ by considering the *m* (by default, *m* = 50) slowest-decaying eigenmodes (from $\lambda_{2}$ to $\lambda_{m+1}$), which dominate the diffusion process. This simplification yields the final closed-form approximation:


$$\begin{array}{c} t^{*}=\max_{s=2,...,m+1}\left(\frac{\ln\left(\lambda_{s}|v_{s}^{⊤}\hat{x}(0)|\right)-\ln(\epsilon )}{\lambda_{s}}\right). \end{array}$$ (16)

The characteristic diffusion time thus provides a quantitative estimate of spatial variability for each gene, where a larger $t^{*}$ value implies a more significant spatial pattern.

To enhance sensitivity to spatial patterns across the wide dynamic range of gene expression, we introduce a rank aggregation strategy. We apply a series of *p*-th power transformations (by default, p∈{1,1.5,2}) to the normalized expression matrix before computing the diffusion time, thereby generating a distinct gene spatial variability ranking for each transformed version. These rankings are then integrated using the lexicographical ordering method to produce a final, robust rank of spatial variability for all genes.

#### Computational complexity.

The computational complexity of SpatioCAD is primarily determined by four key steps. First, constructing the spatial graph is dominated by the *k*-nearest neighbor search, with a complexity of O(NlogN). Second, calculating the Roughness Score for all *G* genes involves a single sparse-dense matrix multiplication, with a complexity of *O*(*k G N*). Third, a key computational advantage of SpatioCAD is that it only requires the *m* eigenmodes corresponding to the smallest non-zero eigenvalues of $L_{\text{ND}}$. These are computed efficiently using iterative solvers with an approximate complexity of *O*(*k m N*), thus avoiding the costly *O*(*N*^3^) required for a full decomposition. Finally, computing the diffusion time $t^{*}$ for all *G* genes, as defined in Eq (16), has a complexity of *O*(*m G N*). Therefore, the overall computational complexity of the SpatioCAD pipeline is approximately *O*((*k* + *m*) *G N*), demonstrating that the method scales linearly with the number of genes (*G*) and spatial locations (*N*).

#### Computational implementation.

SpatioCAD is implemented in Python 3.9 using NumPy [73], SciPy [74], and scikitlearn [75] for core numerical computations. All computational analyses were performed on a high-performance computing cluster with specifications detailed in Table A in S1 Appendix.

### Compared methods

To evaluate the performance of SpatioCAD, we benchmarked it against five existing methods for identifying SVGs: STMiner [19], Sepal [15], SpaGFT [14], SpatialDE [4] and SPARK-X [13]. These methods were selected to provide a representative comparison across complementary formulations, including optimal transport, diffusion, graph-spectral analysis, Gaussian-process modelling, and nonparametric testing. The comparator set combines established approaches with more recent methods, including SpaGFT and STMiner.

To ensure a fair comparison, we filtered out spots with ≤1 detected genes and genes expressed in ≤100 spots. Each method was subsequently run on the common filtered expression matrix using its default parameters.

**STMiner** [19] is a method that leverages optimal transport theory to identify SVGs. This method is available within the Python package STMiner. We applied the core function spatial_high_variable_genes to log-transformed expression counts. Genes were ranked in descending order based on the resulting z-score.

**Sepal** [15] models spatial expression patterns using Fick’s second law of diffusion. This method is available within the Python package sepal. The analysis was performed with the function models.propagate on normalized and scaled data. Genes were ranked in descending order based on the resulting time.

**SpaGFT** [14] utilizes a graph Fourier transform to identify SVGs based on the Fourier mode. This method is available within the Python package SpaGFT. Following standard library size normalization and log-transformation of the count data, we ran the function detect_svg to obtain scores. Genes were ranked in descending order based on the resulting gft_score.

**SpatialDE** [4] employs Gaussian processes to model and identify genes with significant spatial variation. This method is available within the Python package SpatialDE. Following the standard protocol, we first stabilized the data variance (NaiveDE.stabilize) and regressed out the library size effect (NaiveDE.regress_out) before running the main function SpatialDE.run. Genes were ranked in ascending order based on the resulting qval.

**SPARK-X** [13] is a non-parametric method that uses a robust kernel-based statistical test to detect diverse spatial patterns. This method is available within the R package SPARK. We performed the analysis using the function sparkx with the option mixture. Genes were ranked in ascending order based on the resulting adjustedPval.

### Datasets

#### Simulation datasets.

To simulate the tumor microenvironment, we utilized the reference-free module of SRTsim [76] to generate a square-shaped tissue slice containing 1079 randomly distributed spots. To evaluate algorithmic robustness, we implemented two different scenarios: one with square-shaped domains and another with strip-shaped domains (Fig 2A). The tissue slice was spatially partitioned into five distinct domains, denoted as A, B, C, D, and E. In both scenarios, domain E was designated as the tumor core, while domains A-D represented the normal tissue.

We then simulated the gene expression counts by sampling from a Zero-Inflated Negative Binomial (ZINB) distribution using SRTsim, with global parameters set to a zero percentage of 0.01 and a dispersion of 0.3. These genes were assigned to three functional groups: 900 background genes, 200 SVGs, and 50 noise genes.

To disentangle the contributions of heterogeneity in cell density from the diverse spatial expression patterns of genes, we defined the fold-change (FC) for each gene *g* at a given spot *i* as a product of two components: cell density, ρ(i), and Spatial Variability, ${\text{SV}}_{g}(i)$. The relationship is expressed as:


$$\begin{array}{c} {\text{FC}}_{g}(i)=\rho (i)\times {\text{SV}}_{g}(i). \end{array}$$ (17)

Here, the cell density ρ(i) is solely dependent on the spatial location and is designed to simulate variations in cell density, such as the hypercellularity in tumor tissues. We therefore set ρ(i) to 10 for spots in domain E (tumor) and 1 for spots in domains A-D (normal). The second component, ${\text{SV}}_{g}(i)$, is a gene-specific parameter, which governs the intrinsic spatial expression pattern of each gene. Accordingly, we assigned specific ${\text{SV}}_{g}(i)$ values for genes in the three predefined groups. For each scenario, we simulated data under two conditions: with and without noise genes.

**Background Genes.** For background genes, the SV value was maintained at a constant of 1 across all domains. This configuration results in a final FC vector of [1,1,1,1,10] across domains [A, B, C, D, E], thereby simulating an increase in transcript abundance driven solely by the higher cell density in the tumor domain. To model varying expression levels, these genes were assigned different base mean expression values, stratifying them into a high-expression group (base mean of 4) and a low-expression group (base mean of 2).

**SVGs.** The SVGs were assigned a base mean expression of 2 and stratified into two categories: high-signal (100 genes) and low-signal (100 genes). High-signal SVGs were characterized by higher SV ratios in the designated domains (3.0 for domain B, 2.0 for domain C, 2.5 for domain D, and 2.0 for domain E). Conversely, low-signal SVGs exhibited lower SV ratios in each designated domain with the same predefined domain-specific SV ratios. The resulting FC vectors for these SVGs, which reflect the complex interplay between cell density and intrinsic gene activity, are illustrated in Fig 2B.

**Noise Genes.** To simulate non-informative signals, we employed the noise gene generation module in SRTsim, setting a low base mean expression of 0.1. This module distributes expression counts randomly across all spots, independently of the predefined domain architecture and cell density variations. These genes therefore serve as a negative control, representing random biological or technical noise.

For each scenario, simulated datasets were generated using five different random seeds, and the methods were evaluated under both conditions, with and without noise genes.

#### Real datasets.

We applied SpatioCAD and the five compared methods to four publicly available spatial transcriptomics datasets.

**Human breast cancer data.** This dataset, generated from human breast cancer tissue using the 10X Visium platform, is publicly accessible through the 10X Genomics public data repository [23] (https://www.10xgenomics.com/products/xenium-in-situ/preview-dataset-human-breast). It initially comprised 18,085 genes profiled at 4,992 spatial locations. We retained all locations with non-zero expression for at least one gene and filtered out genes expressed in fewer than 100 locations, yielding a final set of 17,633 genes across 4,992 locations for analysis.

The dataset is accompanied by detailed annotations provided by 10X Genomics. In our analysis, we focused on all 11 annotated cell types: invasive, mixed/invasive, DCIS #1, DCIS #2, stromal, immune, adipocytes, stromal/endothelial, mixed, myoepithelial/stromal/immune, and stromal/endothelial/immune.

**Human lung cancer data.** This dataset was generated from human lung cancer tissue using the 10X Visium platform, and is publicly available from the 10X genomics public datasets (https://www.10xgenomics.com/datasets/human-lung-cancer-ffpe-2-standard). It initially comprised 18,085 genes measured across 3,858 spatial locations. With the same filtering method, we retained a final set of 17,269 genes across 3,858 locations for analysis.

**Human malignant gliomas data.** It was generated from human malignant glioma tissue using the 10X Visium platform, and is publicly available from the Gene Expression Omnibus (GEO: GSE194329) [77], which contains five diffuse midline glioma (DMG) and five glioblastoma (GBM) samples. Our analysis focused on the DMG1 sample (sample id: DMG1_short read spatial transcriptomics), with the raw data containing 36,601 genes measured across 4,337 spatial locations. We retained a final set of 15,448 genes across 4,337 locations for analysis with the same filtering method as above. The original study provides annotations for four distinct histopathological regions within the tumor microenvironment: Tumor, Invasive, Hypoxic, and Vascular.

**Human dorsolateral prefrontal cortex data.** This dataset was generated from human dorsolateral prefrontal cortex (DLPFC) tissue using the 10X Visium platform, and is publicly available through the spatialLIBD project [54,78] (https://research.libd.org/spatialLIBD/). The dataset contains 12 tissue sections from three adult human donors, with sample IDs 151507–151510 and 151669–151676. Each section was designed to span the six cortical layers and adjacent white matter. After retaining annotated spots and filtering genes expressed in fewer than 100 spots, the final analysis included 47,329 spatial locations in total, with 9,133–12,262 genes retained per section. We used the provided layer annotations: Layer 1–Layer 6 and WM.

### Details of analyses

#### Data preprocessing.

We performed quality control (QC) on the raw count matrix using the Scanpy package [79]. We removed genes expressed in fewer than 50 (simulated datasets) or 100 (real datasets) locations, as well as locations with no gene detection. This filtered matrix then served as the common input for all methods.

Following this common QC, subsequent preprocessing steps diverged according to the recommended guidelines for each compared method. For SpatioCAD, each gene’s expression vector was normalized such that its total counts summed to 10,000 across all locations to preserve the underlying biological variability. This normalized expression matrix was log-transformed before being input into the model.

#### Evaluation on simulation data.

To compare methods with different output statistics, we evaluated ranking-based SVG recovery using the known simulation labels. Genes were ordered according to the method-specific statistics. For a rank cutoff *k*, let TP(*k*) and FP(*k*) denote the numbers of true SVGs and non-SVGs among the top *k* ranked genes, respectively. Empirical power, defined as the true positive rate (TPR), and the realized false discovery rate (FDR) were calculated as


$$\begin{array}{c} TPR(k)=\frac{TP(k)}{M_{1}},\;FDR(k)=\frac{FP(k)}{TP(k)+FP(k)}, \end{array}$$ (18)

where *M*_1_ is the total number of true SVGs defined for the evaluation. For each FDR threshold α∈{0.01,0.03,0.05,0.075,0.1}, we selected the largest rank cutoff satisfying FDR(k)≤α and plotted the corresponding TPR. If no nonempty ranked prefix satisfied this condition, the TPR was set to zero. This evaluation uses ground-truth labels to characterize ranking performance.

For the ablation and sensitivity analyses, we used both simulation scenarios containing noise genes, with five simulation seeds per scenario. Comparisons were paired by seed. The ablation removed only the RS-based filtering step, while the sensitivity analyses varied one parameter or design choice at a time. All other settings were held fixed within each comparison.

In addition to power at the FDR threshold of 0.05, we evaluated the top 200 ranked genes using the F1 score and the number of simulated noise genes in this selected set. The F1 score was calculated as


$$\begin{array}{c} F1\;score=\frac{2\;TP(200)}{2\;TP(200)+FP(200)+FN(200)}, \end{array}$$ (19)

where TP(200) and FP(200) are the numbers of true SVGs and non-SVGs among the top 200 genes, respectively, and FN(200) is the number of true SVGs in the evaluation set that are not selected. The same ground-truth evaluation set was used across variants, so true SVGs removed by RS filtering remained included when counting false negatives. Metrics were calculated separately for each simulation replicate and summarized as the mean and sample standard deviation across the five seeds within each scenario.

#### Evaluation on real data.

**Overlap between SVGs and housekeeping genes.** To evaluate the propensity of each method to identify false positives, we measured the overlap between the top 1,000 identified SVGs and the HRT Atlas v1.0 database of human housekeeping genes [25]. A higher overlap was considered indicative of a greater likelihood of false positives, as housekeeping genes are generally not expected to exhibit spatial variability.

**Expression-level distribution and ranking association.** To characterize the relationship between SVG selection and gene expression level, we performed a two-stage evaluation of the relationship between spatial variability and gene expression levels. First, we examined the distribution of expression ranks of the top 1,000 SVGs identified by each method relative to all filtered genes. Specifically, all filtered genes were sorted in descending order based on their mean expression level. The rank of each gene *g*, ${\text{Rank}}_{g,\text{original}}$, was subsequently normalized to a range of [0, 1]:


$$\begin{array}{c} {\text{Rank}}_{g,\text{normalized}}=1-\frac{{\text{Rank}}_{g,\text{original}}-1}{G-1}, \end{array}$$ (20)

where *G* represents the total number of filtered genes and ${\text{Rank}}_{g,\text{normalized}}$ thereby quantifies a gene’s relative expression standing. In this framework, a normalized rank of 0 corresponds to the lowest expression value, while 1 indicates the highest. The value 0.5 is used as a descriptive reference: it equals the mean normalized expression rank across all filtered genes and the expected mean rank of a fixed-size gene set selected with equal probability from this background. A smaller absolute deviation of the selected genes’ mean rank from 0.5 indicates greater expression balance by this summary measure, rather than statistical unbiasedness or a biologically optimal selection.

Second, to quantify the dependence of SVG detection on expression level, we calculated the Spearman correlation coefficient [80] (*R*) between the rank of spatial variability and mean expression level of the genes:


$$\begin{array}{c} R=1-\frac{6\sum_{g=1}^{G}d_{g}^{2}}{G(G^{2}-1)}, \end{array}$$ (21)

where $d_{g}$ represents the difference between the two ranks of gene *g*, defined as $d_{g}={\text{Rank}}_{g,\text{original}}-{\text{Rank}}_{g,\text{variability}}$. A Spearman correlation close to zero indicates little monotonic association between expression level and spatial-variability ranking. A positive correlation indicates that more hi*g*hly expressed genes tend to receive higher priority, whereas a negative correlation indicates the opposite tendency.

**Functional diversity of SVGs.** To verify whether the identified SVGs capture a broad spectrum of biological functions, we quantified their functional diversity based on gene clustering. Specifically, all genes were first embedded into a low-dimensional space using Principal Component Analysis (PCA) and Uniform Manifold Approximation and Projection (UMAP) [81]. The Leiden algorithm [82] was then applied to cluster the genes into *M* distinct co-expression modules, denoted as $𝒞=\{C_{1},C_{2},...,C_{M}\}$. We assessed the biological relevance of each cluster through gene set enrichment analysis using the Gene Ontology (GO) database, confirming that these modules correspond to distinct biological functions.

A method capable of capturing functionally diverse SVGs should distribute them across multiple independent modules, rather than concentrating them toward a narrow functional subset. For each method, we calculated the probability distribution of the SVGs across these clusters. Functional diversity was then quantified using the Shannon entropy [83] (*H*):


$$\begin{array}{c} H=-\sum_{k=1}^{M}p_{k}\ln p_{k}, \end{array}$$ (22)

where $p_{k}$ is the proportion of SVGs belonging to cluster $C_{k}$. A higher entropy value indicates that the identified SVGs are more evenly distributed across different functional clusters, reflecting the method’s ability to recover diverse spatial‑functional patterns.

**Spatial coherence of SVGs.** To further assess the quality of SVGs, we evaluated the spatial coherence of the identified SVGs using a neighborhood-based prediction model. This framework is based on the assumption that biologically genuine spatial patterns should exhibit sufficient local smoothness, such that the expression level at any given location can be reliably predicted from its immediate spatial neighbors.

The evaluation was executed in two steps for each location *i*. First, a local linear regression model was trained using exclusively its neighbors, ${𝒩}_{k}(i)$, to capture the local relationship between spatial location and gene expression. Second, we used this fitted model to predict the gene expression at the central location of spot *i* itself. The corresponding prediction error quantifies the local coherence of the expression pattern.

Specifically, for a given gene expression $x_{i}$ at location *i*, the local linear parameter estimates, $\left(\beta_{0},\beta_{1}\right)$, are obtained by minimizing the following least squares objective function:


$$\begin{array}{c} ({\hat{\beta }}_{0},{\hat{\beta }}_{1})=\text{arg}\min_{\beta_{0},\beta_{1}}\sum_{j\in {𝒩}_{k}(i)}(x_{j}-\beta_{0}-\beta_{1}^{⊤}s_{j}{)}^{2}, \end{array}$$ (23)

where ${𝒩}_{k}(i)$ is the set of *k* = 15 nearest neighbors of location *i*, and $s_{j}$ is the spatial coordinate vector of location *j*. The estimated parameters, $\left({\hat{\beta }}_{0},{\hat{\beta }}_{1}\right)$, capturing the local expression trend, were then used to predict the expression at the target location *i*:


$$\begin{array}{c} {\hat{x}}_{i}={\hat{\beta }}_{0}+{\hat{\beta }}_{1}^{⊤}s_{i}. \end{array}$$ (24)

The overall coherence of a given gene and its local neighborhood regions was quantified using the Mean Absolute Error (MAE) of the prediction across all *N* locations:


$$\begin{array}{c} \text{Prediction Error}=\frac{1}{N}\sum_{i=1}^{N}|{\hat{x}}_{i}-x_{i}|. \end{array}$$ (25)

Here, a lower MAE signifies superior coherence, indicating that the gene’s spatial pattern can be reconstructed from its local neighborhood structure.

**Spatial autocorrelation of SVGs.** To validate the spatial significance of the detected genes from a global perspective, we calculated Moran’s *I* statistic [32] for the top-ranked SVGs. This metric serves as a standard measure of spatial autocorrelation, quantifying the extent to which gene expression exhibits clustered spatial patterns rather than a random distribution. A higher average Moran’s *I* indicates a superior capability to prioritize genes with stable spatial structures.

**Statistical stability of SVG detection.** To assess the stability of the detected SVGs for each method, we employed a permutation-based testing framework. For a given SVG identified by a specific method, we randomly shuffled its spatial coordinates while holding the expression profiles of all other genes fixed. The rank of spatial variability in descending order for the permuted gene was then calculated. We quantified statistical stability using an empirical *p*-value derived from *N*_perm_ permutations (by default, *N*_perm_ = 50):


$$\begin{array}{c} P=\frac{1}{N_{\text{perm}}}\sum_{k=1}^{N_{\text{perm}}}𝕀({\text{Rank}}_{k,\text{sv.perm}}\leq {\text{Rank}}_{\text{sv.original}}) \end{array}$$ (26)

where Rank_sv.original_ denotes the spatial variability ranking of the gene in the observed data, ${\text{Rank}}_{k,\text{sv.perm}}$ represents its rank in the *k*-th permutation, and 𝕀(·) is the indicator function. A lower *p*-value indicates higher statistical stability of the identified spatial pattern. With *N*_perm_ = 50, the empirical *p*-values have a resolution of 0.02. We selected this permutation budget to balance computational cost and resolution, particularly given the cost of repeated STMiner ranking evaluations. For comparison, the values were summarized in three categories: *p* < 0.05, 0.05≤p<0.1, and p≥0.1. The resulting candidate-set summaries were evaluated together with local prediction error and Moran’s *I* to characterize the spatial support of the identified genes.

**SVG clustering.** To systematically categorize the identified SVGs into distinct spatial modules, SpatioCAD employs a clustering step leveraging their spatial diffusion profiles. The spectral decomposition of the diffusion process (Eq (12)) provides a natural feature space for gene representation. Specifically, the projection of a gene’s expression ($\hat{x}$) onto the *k*-th eigenvector ($v_{k}$), quantified by the coefficient $v_{k}^{⊤}\hat{x}$, measures the alignment of the gene with the corresponding eigenmode. To group genes with similar spatial topologies, we constructed a low-dimensional spectral embedding, $\left(v_{2}^{⊤}\hat{x},...,v_{m+1}^{⊤}\hat{x}\right)$, and subsequently applied *k*-means clustering to these feature vectors.

Following the identification of distinct gene modules, SpatioCAD derived a representative spatial pattern for each gene set. First, we computed an expression ratio map by calculating the fraction of genes within the cluster that are expressed at each location. The final spatial pattern was then generated by applying a threshold to this map: specifically, the value at any given location was set to the cluster’s mean expression if its local expression ratio exceeds a defined cutoff (by default, 20%); otherwise, it was assigned a value of zero.

**UMAP visualization.** For the human malignant gliomas dataset, we projected the normalized spatial expression profiles of the top 1,000 SVGs into a two-dimensional space using the Uniform Manifold Approximation and Projection (UMAP) [81]. This visualization aimed to validate the coherence of our previously identified gene clusters. In the resulting UMAP embedding, each point corresponds to a gene, and the proximity between points is indicative of the similarity in their spatial expression profiles.

**Expression-matched Moran’s *I z*-score.** To evaluate whether the top-ranked SVGs exhibit spatial structure beyond that expected from their expression level, we calculated expression-matched Moran’s *I z*-score for the top 500 SVGs identified by each method. For each gene *g*, Moran’s *I* was first computed on the spatial nearest-neighbour graph constructed from spot coordinates. We then matched *g* to a local background set of genes with similar expression properties, using two features: log-transformed mean expression, $\log(1+{\bar{x}}_{g})$, and detection rate, defined as the proportion of spots with non-zero expression. These two features were standardized across genes, and the 200 nearest genes in this two-dimensional space were used as the matched background set ${ℬ}_{g}$.

The matched Moran’s *I z*-score was then defined as


$$\begin{array}{c} z_{g}=\frac{I_{g}-{mean}_{h\in {ℬ}_{g}}(I_{h})}{{std}_{h\in {ℬ}_{g}}(I_{h})}, \end{array}$$ (27)

where $I_{g}$ denotes the Moran’s *I* of gene *g*, and ${ℬ}_{g}$ represents the matched background *g*ene set. Thus, $z_{g}$ quantifies whether a gene shows stronger spatial autocorrelation than expected among genes with similar expression characteristics. A higher value indicates that the selected genes retain stronger spatial organization after controlling for expression abundance and detection frequency, suggesting a greater ability to recover genuine spatially variable genes rather than expression-driven artefacts.

**Gene set enrichment analysis.** We performed gene set enrichment analysis using the Python package gseapy [84], querying against several gene set libraries: GO_Biological_Process_2025, GO_Cellular_Component_2025, and KEGG_2021_Human. Enriched terms with an adjusted *p*-value < 0.05 were considered statistically significant.

## Supporting information

S1 Appendix Supplementary theoretical notes and tables. This file contains theoretical notes supporting the graph diffusion framework, and two tables detailing the computational environment and gene counts before and after filtering.
(PDF)

S2 Appendix Supplementary figures (Part I). This file contains supplementary figures (Figs A–Z) and their legends supporting the simulation analyses and real-data applications of SpatioCAD.
(PDF)

S3 Appendix Supplementary figures (Part II). This file contains supplementary figures (Figs A–J) and their legends supporting the real-data applications of SpatioCAD.
(PDF)

## References

[1] Moses L, Pachter L. Museum of spatial transcriptomics. Nat Methods. 2022;19(5):534–46. doi: 10.1038/s41592-022-01409-2 35273392

[2] Asp M, Bergenstråhle J, Lundeberg J. Spatially Resolved Transcriptomes-Next Generation Tools for Tissue Exploration. Bioessays. 2020;42(10):e1900221. doi: 10.1002/bies.201900221 32363691

[3] Li Z, Wang T, Liu P, Huang Y. SpatialDM for rapid identification of spatially co-expressed ligand-receptor and revealing cell-cell communication patterns. Nat Commun. 2023;14(1):3995. doi: 10.1038/s41467-023-39608-w 37414760 PMC10325966

[4] Svensson V, Teichmann SA, Stegle O. SpatialDE: identification of spatially variable genes. Nat Methods. 2018;15(5):343–6. doi: 10.1038/nmeth.4636 29553579 PMC6350895

[5] Moffitt JR, Bambah-Mukku D, Eichhorn SW, Vaughn E, Shekhar K, Perez JD, et al. Molecular, spatial, and functional single-cell profiling of the hypothalamic preoptic region. Science. 2018;362(6416):eaau5324. doi: 10.1126/science.aau5324 30385464 PMC6482113

[6] Ståhl PL, Salmén F, Vickovic S, Lundmark A, Navarro JF, Magnusson J, et al. Visualization and analysis of gene expression in tissue sections by spatial transcriptomics. Science. 2016;353(6294):78–82. doi: 10.1126/science.aaf2403 27365449

[7] Lohoff T, Ghazanfar S, Missarova A, Koulena N, Pierson N, Griffiths JA, et al. Integration of spatial and single-cell transcriptomic data elucidates mouse organogenesis. Nat Biotechnol. 2022;40(1):74–85. doi: 10.1038/s41587-021-01006-2 34489600 PMC8763645

[8] Wu SZ, Al-Eryani G, Roden DL, Junankar S, Harvey K, Andersson A, et al. A single-cell and spatially resolved atlas of human breast cancers. Nat Genet. 2021;53(9):1334–47. doi: 10.1038/s41588-021-00911-1 34493872 PMC9044823

[9] Sun S, Zhu J, Zhou X. Statistical analysis of spatial expression patterns for spatially resolved transcriptomic studies. Nat Methods. 2020;17(2):193–200. doi: 10.1038/s41592-019-0701-7 31988518 PMC7233129

[10] Wu L, Yan J, Bai Y, Chen F, Zou X, Xu J, et al. An invasive zone in human liver cancer identified by Stereo-seq promotes hepatocyte-tumor cell crosstalk, local immunosuppression and tumor progression. Cell Res. 2023;33(8):585–603. doi: 10.1038/s41422-023-00831-1 37337030 PMC10397313

[11] Sziraki A, Lu Z, Lee J, Banyai G, Anderson S, Abdulraouf A, et al. A global view of aging and Alzheimer’s pathogenesis-associated cell population dynamics and molecular signatures in human and mouse brains. Nat Genet. 2023;55(12):2104–16. doi: 10.1038/s41588-023-01572-y 38036784 PMC10703679

[12] Yu M, Risacher SL, Nho KT, Wen Q, Oblak AL, Unverzagt FW, et al. Spatial transcriptomic patterns underlying amyloid-β and tau pathology are associated with cognitive dysfunction in Alzheimer’s disease. Cell Rep. 2024;43(2):113691. doi: 10.1016/j.celrep.2024.113691 38244198 PMC10926093

[13] Zhu J, Sun S, Zhou X. SPARK-X: non-parametric modeling enables scalable and robust detection of spatial expression patterns for large spatial transcriptomic studies. Genome Biol. 2021;22(1):184. doi: 10.1186/s13059-021-02404-0 34154649 PMC8218388

[14] Chang Y, Liu J, Jiang Y, Ma A, Yeo YY, Guo Q, et al. Graph Fourier transform for spatial omics representation and analyses of complex organs. Nat Commun. 2024;15(1):7467. doi: 10.1038/s41467-024-51590-5 39209833 PMC11362340

[15] Andersson A, Lundeberg J. sepal: identifying transcript profiles with spatial patterns by diffusion-based modeling. Bioinformatics. 2021;37(17):2644–50. doi: 10.1093/bioinformatics/btab164 33704427 PMC8428601

[16] Weber LM, Saha A, Datta A, Hansen KD, Hicks SC. nnSVG for the scalable identification of spatially variable genes using nearest-neighbor Gaussian processes. Nat Commun. 2023;14(1):4059. doi: 10.1038/s41467-023-39748-z 37429865 PMC10333391

[17] Hao M, Hua K, Zhang X. SOMDE: a scalable method for identifying spatially variable genes with self-organizing map. Bioinformatics. 2021;37(23):4392–8. doi: 10.1093/bioinformatics/btab471 34165490

[18] Spill F, Reynolds DS, Kamm RD, Zaman MH. Impact of the physical microenvironment on tumor progression and metastasis. Curr Opin Biotechnol. 2016;40:41–8. doi: 10.1016/j.copbio.2016.02.007 26938687 PMC4975620

[19] Sun P, Bush SJ, Wang S, Jia P, Li M, Xu T, et al. STMiner: Gene-centric spatial transcriptomics for deciphering tumor tissues. Cell Genom. 2025;5(2):100771. doi: 10.1016/j.xgen.2025.100771 39947134 PMC11872602

[20] Mukherjee D, Guha A, Solomon JM, Sun Y, Yurochkin M. Outlier-Robust Optimal Transport. In: Proceedings of the 38th International Conference on Machine Learning, 2021. 7850–60. https://proceedings.mlr.press/v139/mukherjee21a.html

[21] Nietert S, Goldfeld Z, Cummings R. Outlier-robust optimal transport: duality, structure, and statistical analysis. In: Proceedings of the 25th International Conference on Artificial Intelligence and Statistics, 2022. 11691–719. https://proceedings.mlr.press/v151/nietert22a.html

[22] Statello L, Guo C-J, Chen L-L, Huarte M. Gene regulation by long non-coding RNAs and its biological functions. Nat Rev Mol Cell Biol. 2021;22(2):96–118. doi: 10.1038/s41580-020-00315-9 33353982 PMC7754182

[23] Janesick A, Shelansky R, Gottscho AD, Wagner F, Williams SR, Rouault M, et al. High resolution mapping of the tumor microenvironment using integrated single-cell, spatial and in situ analysis. Nat Commun. 2023;14(1):8353. doi: 10.1038/s41467-023-43458-x 38114474 PMC10730913

[24] Keren L, Bosse M, Marquez D, Angoshtari R, Jain S, Varma S, et al. A Structured Tumor-Immune Microenvironment in Triple Negative Breast Cancer Revealed by Multiplexed Ion Beam Imaging. Cell. 2018;174(6):1373-1387.e19. doi: 10.1016/j.cell.2018.08.039 30193111 PMC6132072

[25] Hounkpe BW, Chenou F, de Lima F, De Paula EV. HRT Atlas v1.0 database: redefining human and mouse housekeeping genes and candidate reference transcripts by mining massive RNA-seq datasets. Nucleic Acids Res. 2021;49(D1):D947–55. doi: 10.1093/nar/gkaa609 32663312 PMC7778946

[26] Jen J, Wang Y-C. Zinc finger proteins in cancer progression. J Biomed Sci. 2016;23(1):53. doi: 10.1186/s12929-016-0269-9 27411336 PMC4944467

[27] Smrcka AV. G protein βγ subunits: central mediators of G protein-coupled receptor signaling. Cell Mol Life Sci. 2008;65(14):2191–214. doi: 10.1007/s00018-008-8006-5 18488142 PMC2688713

[28] Di Rosa M, Malaguarnera G, De Gregorio C, Drago F, Malaguarnera L. Evaluation of CHI3L-1 and CHIT-1 expression in differentiated and polarized macrophages. Inflammation. 2013;36(2):482–92. doi: 10.1007/s10753-012-9569-8 23149946

[29] Zhou J, Zhu J, Yu S-J, Ma H-L, Chen J, Ding X-F, et al. Sodium-glucose co-transporter-2 (SGLT-2) inhibition reduces glucose uptake to induce breast cancer cell growth arrest through AMPK/mTOR pathway. Biomed Pharmacother. 2020;132:110821. doi: 10.1016/j.biopha.2020.110821 33068934

[30] Clarke R, Cook KL, Hu R, Facey COB, Tavassoly I, Schwartz JL, et al. Endoplasmic reticulum stress, the unfolded protein response, autophagy, and the integrated regulation of breast cancer cell fate. Cancer Res. 2012;72(6):1321–31. doi: 10.1158/0008-5472.CAN-11-3213 22422988 PMC3313080

[31] Kairouz R, Parmar J, Lyons RJ, Swarbrick A, Musgrove EA, Daly RJ. Hormonal regulation of the Grb14 signal modulator and its role in cell cycle progression of MCF-7 human breast cancer cells. J Cell Physiol. 2005;203(1):85–93. doi: 10.1002/jcp.20199 15372466

[32] Moran PAP. Notes on continuous stochastic phenomena. Biometrika. 1950;37(1–2):17–23. doi: 10.1093/biomet/37.1-2.17 15420245

[33] Salmans ML, Zhao F, Andersen B. The estrogen-regulated anterior gradient 2 (AGR2) protein in breast cancer: a potential drug target and biomarker. Breast Cancer Res. 2013;15(2):204. doi: 10.1186/bcr3408 23635006 PMC3672732

[34] Kamei M, Yamashita S-I, Tokuishi K, Hashioto T, Moroga T, Suehiro S, et al. HE4 expression can be associated with lymph node metastases and disease-free survival in breast cancer. Anticancer Res. 2010;30(11):4779–83. 21115941

[35] Mi Y, Shi Z, Li J. Spata19 is critical for sperm mitochondrial function and male fertility. Mol Reprod Dev. 2015;82(11):907–13. doi: 10.1002/mrd.22536 26265198

[36] Hecker L, Vittal R, Jones T, Jagirdar R, Luckhardt TR, Horowitz JC, et al. NADPH oxidase-4 mediates myofibroblast activation and fibrogenic responses to lung injury. Nat Med. 2009;15(9):1077–81. doi: 10.1038/nm.2005 19701206 PMC2743335

[37] Myllärniemi M, Lindholm P, Ryynänen MJ, Kliment CR, Salmenkivi K, Keski-Oja J, et al. Gremlin-mediated decrease in bone morphogenetic protein signaling promotes pulmonary fibrosis. Am J Respir Crit Care Med. 2008;177(3):321–9. doi: 10.1164/rccm.200706-945OC 17975199 PMC2218851

[38] Sun GZ, Zhao TW. Lung adenocarcinoma pathology stages related gene identification. Math Biosci Eng. 2019;17(1):737–46. doi: 10.3934/mbe.2020038 31731374

[39] Beer DG, Kardia SLR, Huang C-C, Giordano TJ, Levin AM, Misek DE, et al. Gene-expression profiles predict survival of patients with lung adenocarcinoma. Nat Med. 2002;8(8):816–24. doi: 10.1038/nm733 12118244

[40] Guo X, Wang W, Hu J, Feng K, Pan Y, Zhang L, et al. Lentivirus-mediated RNAi knockdown of NUPR1 inhibits human nonsmall cell lung cancer growth in vitro and in vivo. Anat Rec (Hoboken). 2012;295(12):2114–21. doi: 10.1002/ar.22571 22961798

[41] Hu C-AA, Klopfer EI, Ray PE. Human apolipoprotein L1 (ApoL1) in cancer and chronic kidney disease. FEBS Lett. 2012;586(7):947–55. doi: 10.1016/j.febslet.2012.03.002 22569246 PMC3349435

[42] Watt B, van Niel G, Raposo G, Marks MS. PMEL: a pigment cell-specific model for functional amyloid formation. Pigment Cell Melanoma Res. 2013;26(3):300–15. doi: 10.1111/pcmr.12067 23350640 PMC3633693

[43] Ligon KL, Alberta JA, Kho AT, Weiss J, Kwaan MR, Nutt CL, et al. The oligodendroglial lineage marker OLIG2 is universally expressed in diffuse gliomas. J Neuropathol Exp Neurol. 2004;63(5):499–509. doi: 10.1093/jnen/63.5.499 15198128

[44] Metselaar DS, du Chatinier A, Meel MH, Ter Huizen G, Waranecki P, Goulding JR, et al. AURKA and PLK1 inhibition selectively and synergistically block cell cycle progression in diffuse midline glioma. iScience. 2022;25(6):104398. doi: 10.1016/j.isci.2022.104398 35637734 PMC9142558

[45] Wang S, Song R, Wang Z, Jing Z, Wang S, Ma J. S100A8/A9 in Inflammation. Front Immunol. 2018;9:1298. doi: 10.3389/fimmu.2018.01298 PMC6004386 29942307

[46] Vogler M. BCL2A1: the underdog in the BCL2 family. Cell Death Differ. 2012;19(1):67–74. doi: 10.1038/cdd.2011.158 22075983 PMC3252829

[47] Coussens LM, Werb Z. Inflammation and cancer. Nature. 2002;420(6917):860–7. doi: 10.1038/nature01322 12490959 PMC2803035

[48] Tang W, Lo CWS, Ma W, Chu ATW, Tong AHY, Chung BHY. Revealing the role of SPP1+ macrophages in glioma prognosis and therapeutic targeting by investigating tumor-associated macrophage landscape in grade 2 and 3 gliomas. Cell Biosci. 2024;14(1):37. doi: 10.1186/s13578-024-01218-4 38515213 PMC10956315

[49] Han J, Jing Y, Han F, Sun P. Comprehensive analysis of expression, prognosis and immune infiltration for TIMPs in glioblastoma. BMC Neurol. 2021;21(1):447. doi: 10.1186/s12883-021-02477-1 34781885 PMC8591954

[50] Zhang Y, Chen K, Sloan SA, Bennett ML, Scholze AR, O’Keeffe S, et al. An RNA-sequencing transcriptome and splicing database of glia, neurons, and vascular cells of the cerebral cortex. J Neurosci. 2014;34(36):11929–47. doi: 10.1523/JNEUROSCI.1860-14.2014 25186741 PMC4152602

[51] Zeisel A, Muñoz-Manchado AB, Codeluppi S, Lönnerberg P, La Manno G, Juréus A, et al. Brain structure. Cell types in the mouse cortex and hippocampus revealed by single-cell RNA-seq. Science. 2015;347(6226):1138–42. doi: 10.1126/science.aaa1934 25700174

[52] Flügge G, Araya-Callis C, Garea-Rodriguez E, Stadelmann-Nessler C, Fuchs E. NDRG2 as a marker protein for brain astrocytes. Cell Tissue Res. 2014;357(1):31–41. doi: 10.1007/s00441-014-1837-5 24816982 PMC4077251

[53] Hanahan D, Weinberg RA. Hallmarks of cancer: the next generation. Cell. 2011;144(5):646–74. doi: 10.1016/j.cell.2011.02.013 21376230

[54] Maynard KR, Collado-Torres L, Weber LM, Uytingco C, Barry BK, Williams SR, et al. Transcriptome-scale spatial gene expression in the human dorsolateral prefrontal cortex. Nat Neurosci. 2021;24(3):425–36. doi: 10.1038/s41593-020-00787-0 33558695 PMC8095368

[55] Pavlos NJ, Grønborg M, Riedel D, Chua JJE, Boyken J, Kloepper TH, et al. Quantitative analysis of synaptic vesicle Rabs uncovers distinct yet overlapping roles for Rab3a and Rab27b in Ca2+-triggered exocytosis. J Neurosci. 2010;30(40):13441–53. doi: 10.1523/JNEUROSCI.0907-10.2010 20926670 PMC6634719

[56] Castiglioni V, Faedo A, Onorati M, Bocchi VD, Li Z, Iennaco R, et al. Dynamic and Cell-Specific DACH1 Expression in Human Neocortical and Striatal Development. Cereb Cortex. 2019;29(5):2115–24. doi: 10.1093/cercor/bhy092 29688344 PMC6458905

[57] Griffiths SC, Schwab RA, El Omari K, Bishop B, Iverson EJ, Malinauskas T, et al. Hedgehog-Interacting Protein is a multimodal antagonist of Hedgehog signalling. Nat Commun. 2021;12(1):7171. doi: 10.1038/s41467-021-27475-2 34887403 PMC8660895

[58] Ishina IA, Zakharova MY, Kurbatskaia IN, Mamedov AE, Belogurov AAJ, Gabibov AG. MHC class II presentation in autoimmunity. Cells. 2023;12(2):314. doi: 10.3390/cells12020314 PMC9856717 36672249

[59] Schetters STT, Gomez-Nicola D, Garcia-Vallejo JJ, Van Kooyk Y. Neuroinflammation: microglia and T cells get ready to tango. Front Immunol. 2018;8:1905. doi: 10.3389/fimmu.2017.01905 PMC5788906 29422891

[60] Uhlar CM, Grehan S, Steel DM, Steinkasserer A, Whitehead AS. Use of the acute phase serum amyloid A2 (SAA2) gene promoter in the analysis of pro- and anti-inflammatory mediators: differential kinetics of SAA2 promoter induction by IL-1 beta and TNF-alpha compared to IL-6. J Immunol Methods. 1997;203(2):123–30. doi: 10.1016/s0022-1759(96)00220-7 9149805

[61] Zhang X, Shao J, Wang C, Liu C, Hao H, Li X, et al. TMC7 functions as a suppressor of Piezo2 in primary sensory neurons blunting peripheral mechanotransduction. Cell Rep. 2024;43(4):114014. doi: 10.1016/j.celrep.2024.114014 38568807

[62] Kuo M-W, Wang C-H, Wu H-C, Chang S-J, Chuang Y-J. Soluble THSD7A is an N-glycoprotein that promotes endothelial cell migration and tube formation in angiogenesis. PLoS One. 2011;6(12):e29000. doi: 10.1371/journal.pone.0029000 22194972 PMC3237571

[63] McCracken LM, Lowes DC, Salling MC, Carreau-Vollmer C, Odean NN, Blednov YA, et al. Glycine receptor α3 and α2 subunits mediate tonic and exogenous agonist-induced currents in forebrain. Proc Natl Acad Sci U S A. 2017;114(34):E7179–86. doi: 10.1073/pnas.1703839114 28784756 PMC5576794

[64] Saiselet M, Rodrigues-Vitória J, Tourneur A, Craciun L, Spinette A, Larsimont D, et al. Transcriptional output, cell-type densities, and normalization in spatial transcriptomics. J Mol Cell Biol. 2020;12(11):906–8. doi: 10.1093/jmcb/mjaa028 32573704 PMC7883818

[65] Bhuva DD, Tan CW, Salim A, Marceaux C, Pickering MA, Chen J, et al. Library size confounds biology in spatial transcriptomics data. Genome Biol. 2024;25(1):99. doi: 10.1186/s13059-024-03241-7 38637899 PMC11025268

[66] Shang L, Wu P, Zhou X. Statistical identification of cell type-specific spatially variable genes in spatial transcriptomics. Nat Commun. 2025;16(1):1059. doi: 10.1038/s41467-025-56280-4 39865128 PMC11770176

[67] Su H, Wu Y, Chen B, Cui Y. STANCE: a unified statistical model to detect cell-type-specific spatially variable genes in spatial transcriptomics. Nat Commun. 2025;16(1):1793. doi: 10.1038/s41467-025-57117-w 39979358 PMC11842841

[68] Wang Z, Zeng Y, Tan Z, Chen Y, Huang X, Zhao H, et al. A unified framework for identification of cell-type-specific spatially variable genes in spatial transcriptomic studies. Proc Natl Acad Sci U S A. 2025;122(46):e2503952122. doi: 10.1073/pnas.2503952122 41223223 PMC12646224

[69] Zelnik-Manor L, Perona P. Self-tuning spectral clustering. In: Saul L, Weiss Y, Bottou L, editors. Advances in Neural Information Processing Systems. Cambridge, MA: MIT Press. 2004. p. 1601–8. https://proceedings.neurips.cc/paper/2004/hash/40173ea48d9567f1f393b20c855bb40b-Abstract.html

[70] Shuman DI, Narang SK, Frossard P, Ortega A, Vandergheynst P. The emerging field of signal processing on graphs: Extending high-dimensional data analysis to networks and other irregular domains. IEEE Signal Process Mag. 2013;30(3):83–98. doi: 10.1109/msp.2012.2235192

[71] Chung FRK. Spectral graph theory. Providence, RI: American Mathematical Society. 1997. doi: 10.1090/cbms/092

[72] Schwarz G. Estimating the dimension of a model. Ann Stat. 1978;6(2):461–4. doi: 10.1214/aos/1176344136

[73] Harris CR, Millman KJ, van der Walt SJ, Gommers R, Virtanen P, Cournapeau D, et al. Array programming with NumPy. Nature. 2020;585(7825):357–62. doi: 10.1038/s41586-020-2649-2 32939066 PMC7759461

[74] Virtanen P, Gommers R, Oliphant TE, Haberland M, Reddy T, Cournapeau D, et al. SciPy 1.0: fundamental algorithms for scientific computing in Python. Nat Methods. 2020;17(3):261–72. doi: 10.1038/s41592-019-0686-2 32015543 PMC7056644

[75] Pedregosa F, Varoquaux G, Gramfort A, Michel V, Thirion B, Grisel O. Scikit-learn: Machine learning in Python. J Mach Learn Res. 2011;12(85):2825–30.

[76] Zhu J, Shang L, Zhou X. SRTsim: spatial pattern preserving simulations for spatially resolved transcriptomics. Genome Biol. 2023;24(1):39. doi: 10.1186/s13059-023-02879-z 36869394 PMC9983268

[77] Ren Y, Huang Z, Zhou L, Xiao P, Song J, He P, et al. Spatial transcriptomics reveals niche-specific enrichment and vulnerabilities of radial glial stem-like cells in malignant gliomas. Nat Commun. 2023;14(1):1028. doi: 10.1038/s41467-023-36707-6 36823172 PMC9950149

[78] Pardo B, Spangler A, Weber LM, Page SC, Hicks SC, Jaffe AE, et al. spatialLIBD: an R/Bioconductor package to visualize spatially-resolved transcriptomics data. BMC Genomics. 2022;23(1):434. doi: 10.1186/s12864-022-08601-w 35689177 PMC9188087

[79] Wolf FA, Angerer P, Theis FJ. SCANPY: large-scale single-cell gene expression data analysis. Genome Biol. 2018;19(1):15. doi: 10.1186/s13059-017-1382-0 29409532 PMC5802054

[80] Spearman C. The proof and measurement of association between two things. In: Jenkins JJ, Paterson DG, editors. Studies in individual differences: The search for intelligence. East Norwalk: Appleton-Century-Crofts. 1961. p. 45–58. doi: 10.1037/11491-005

[81] McInnes L, Healy J, Melville J. UMAP: Uniform manifold approximation and projection for dimension reduction. In: 2018. 10.48550/arXiv.1802.03426

[82] Traag VA, Waltman L, van Eck NJ. From Louvain to Leiden: guaranteeing well-connected communities. Sci Rep. 2019;9(1):5233. doi: 10.1038/s41598-019-41695-z 30914743 PMC6435756

[83] Shannon CE. A Mathematical Theory of Communication. Bell System Technical Journal. 1948;27(4):623–56. doi: 10.1002/j.1538-7305.1948.tb00917.x

[84] Fang Z, Liu X, Peltz G. GSEApy: a comprehensive package for performing gene set enrichment analysis in Python. Bioinformatics. 2023;39(1):btac757. doi: 10.1093/bioinformatics/btac757 36426870 PMC9805564

