# Supplementary material for: SpatioCAD: Context-aware graph diffusion model for pinpointing spatially variable genes in heterogeneous tissues
Source: PLoS Comput Biol. 2026 Nov 2;22(11):e1014923. doi: 10.1371/journal.pcbi.1014923 (PMC13750662; doi:10.1371/journal.pcbi.1014923)
Supplement: Supplementary file 1 [file pcbi.1014923.s001.pdf]

S1 Appendix

Supplementary theoretical notes and tables

Supporting information for
"SpatioCAD: Context-aware graph diffusion model for pinpointing spatially variable genes in heterogeneous tissues"

Note A. Spectral Interpretation of the Roughness Score and High-Frequency Components

To explain the relationship between the Roughness Score and the frequency components of the gene expression signal, we analyze the score from the perspective of spectral graph theory.

Let $\mathbf{L}$ be the graph Laplacian matrix. The spectral decomposition of $\mathbf{L}$ is:

$$\mathbf{L} = \mathbf{V}\Lambda\mathbf{V}^\top, \tag{1}$$

where $\Lambda = \mathrm{diag}(\lambda_1, \lambda_2, \ldots, \lambda_n)$ is the diagonal matrix of eigenvalues sorted in increasing order ($0 = \lambda_1 \leq \lambda_2 \leq \cdots \leq \lambda_N$), and $\mathbf{V} = (\mathbf{v}_1, \mathbf{v}_2, \ldots, \mathbf{v}_N)$ is the orthogonal matrix of corresponding eigenvectors. In the context of graph signal processing, the eigenvectors $\mathbf{v}_i$ represent the graph Fourier modes, and the eigenvalues λ_i represent the graph frequencies. Smaller eigenvalues correspond to low-frequency components, while larger eigenvalues correspond to high-frequency components.

Notice that any graph signal $\mathbf{x}$ can be projected onto the spectral domain using the Graph Fourier Transform:

$$\tilde{\mathbf{x}} = \mathbf{V}^\top\mathbf{x} \implies \mathbf{x} = \mathbf{V}\tilde{\mathbf{x}} = \sum_{i=1}^{N} \tilde{x}_i \mathbf{v}_i, \tag{2}$$

where $\tilde{x}_i$ is the projection coefficient of the signal on the i-th frequency component.

Recall the definition of the Roughness Score:

$$\text{Roughness Score} = \|\mathbf{L}\mathbf{x}\|_2. \tag{3}$$

Substituting the spectral decomposition of $\mathbf{x}$ into this equation, we obtain:

$$\begin{aligned} \|\mathbf{L}\mathbf{x}\|_2^2 &= (\mathbf{L}\mathbf{x})^\top(\mathbf{L}\mathbf{x}) \\ &= (\mathbf{V}\Lambda\mathbf{V}^\top\mathbf{x})^\top(\mathbf{V}\Lambda\mathbf{V}^\top\mathbf{x}) \\ &= (\Lambda\tilde{\mathbf{x}})^\top(\Lambda\tilde{\mathbf{x}}) \\ &= \sum_{i=1}^{N} \tilde{x}_i^2 \lambda_i^2. \end{aligned} \tag{4}$$

Therefore, a high Roughness Score serves as a robust indicator that the gene expression signal is dominated by high-frequency spatial noise rather than smooth, spatially coherent biological domains.

Note B. Spectral Properties of the NAGD Laplacian

In this section, we analyze the spectral properties of the Node-Attributed Graph Diffusion (NAGD) Laplacian matrix, $\mathbf{L}_{\text{ND}}$. First, we analyze the steady state of the system. Recall that the NAGD Laplacian matrix is defined as $\mathbf{L}_{\text{ND}} = \mathbf{N}^{-1}\mathbf{D}_{\mathbf{M}} - \mathbf{N}^{-1/2}\mathbf{M}\mathbf{N}^{-1/2}$, where $\mathbf{M} = \mathbf{A} \odot \mathbf{W}$. Since $\mathbf{N}$ and $\mathbf{D}_{\mathbf{M}}$ are both diagonal, $\mathbf{L}_{\text{ND}}$ can be factorized as a symmetrically normalized Laplacian:

$$\mathbf{L}_{\text{ND}} = \mathbf{N}^{-1/2}(\mathbf{D}_{\mathbf{M}} - \mathbf{M})\mathbf{N}^{-1/2} = \mathbf{N}^{-1/2}\mathbf{L}_{\mathbf{M}}\mathbf{N}^{-1/2}, \tag{5}$$

where $\mathbf{L_M}$ is the standard Laplacian of the weighted graph defined by $\mathbf{M}$. For a connected graph, $\mathbf{L_M}$ possesses a unique zero eigenvalue with the corresponding eigenvector $\mathbf{1}$, satisfying $\mathbf{L_M 1} = \mathbf{0}$. Consequently, due to the matrix congruence, $\mathbf{L}_{\text{ND}}$ also possesses a unique zero eigenvalue ($\lambda_1 = 0$), with all remaining eigenvalues being strictly positive ($\lambda_k > 0$ for $k \geq 2$).

To derive the first eigenvector $\mathbf{v}_1$ associated with the zero eigenvalue of $\mathbf{L}_{\text{ND}}$, we solve the equation $\mathbf{L}_{\text{ND}}\mathbf{v}_1 = \mathbf{0}$. Substituting the factorization from Eq (5) yields:

$$\mathbf{N}^{-1/2}\mathbf{L_M}(\mathbf{N}^{-1/2}\mathbf{v}_1) = \mathbf{0} \implies \mathbf{L_M}(\mathbf{N}^{-1/2}\mathbf{v}_1) = \mathbf{0}. \tag{6}$$

This implies that $\mathbf{N}^{-1/2}\mathbf{v}_1$ must be proportional to $\mathbf{1}$. Therefore, the normalized eigenvector corresponding to the unique steady state is:

$$\mathbf{v}_1 = c \cdot \mathbf{N}^{1/2}\mathbf{1} = c \cdot (\sqrt{n_1}, \sqrt{n_2}, \dots, \sqrt{n_N})^\top, \tag{7}$$

where c is the normalization constant.

Consequently, the steady state of the system, $\mathbf{x}^*$, is given by:

$$\mathbf{x}^* = \lim_{t\to\infty} \mathbf{x}(t) = \mathbf{N}^{1/2}(\lim_{t\to\infty} \hat{\mathbf{x}}(t)) = \mathbf{N}^{1/2}(c \cdot \mathbf{N}^{1/2}\mathbf{1}) = c \cdot \mathbf{N1} = c \cdot \mathbf{n}. \tag{8}$$

This proves that the diffusion process evolves to a unique steady state where gene expression is strictly proportional to the cell density $\mathbf{n}$.

Second, we verify the conservation of the total signal. The time derivative of the total expression sum is obtained by summing the NAGD equation over all nodes:

$$\frac{d}{dt}\sum_{i=1}^{N} \mathbf{x}_i(t) = -\sum_{i=1}^{N}\sum_{j=1}^{N} m_{ij}\left(\frac{\mathbf{x}_i(t)}{n_i} - \frac{\mathbf{x}_j(t)}{n_j}\right). \tag{9}$$

Since the weighted adjacency matrix is symmetric ($m_{ij} = m_{ji}$), the summation terms cancel out pairwise:

$$\sum_{i,j} m_{ij}\frac{\mathbf{x}_i(t)}{n_i} - \sum_{i,j} m_{ij}\frac{\mathbf{x}_j(t)}{n_j} = \sum_{i,j} m_{ij}\frac{\mathbf{x}_i(t)}{n_i} - \sum_{j,i} m_{ji}\frac{\mathbf{x}_i(t)}{n_i} = 0. \tag{10}$$

Thus, the total signal sum $\sum_{i=1}^{N} \mathbf{x}_i(t)$ is conserved throughout the diffusion process.

Note C. Connectivity requirement and stationary contrasts between disconnected components

Let the spatial graph have C connected components, $V_1, \dots, V_C$, and assume positive node densities. Under the assumption of the diffusion model, no diffusion occurs between different components. The total signal within each component is therefore conserved. Consequently, the steady state satisfies

$$x_i^* = n_i \frac{\sum_{j\in V_c} x_j(0)}{\sum_{j\in V_c} n_j}, \qquad i \in V_c.$$

The proportionality constant can differ between components. Thus, equal total expression across the entire graph does not ensure a common steady state across genes when the graph is disconnected.

To illustrate the consequence for diffusion-based scoring, consider two disconnected components with uniform densities $n_i = 1$. Let

$$x_i(0) = \begin{cases} a, & i \in V_1, \\ 0, & i \in V_2, \end{cases} \qquad a > 0.$$

This signal represents a region-restricted expression pattern. However, it is constant within each component, so every edge-wise expression difference is zero. Hence

$$\frac{dx(t)}{dt} = 0 \quad \text{for all } t \geq 0.$$

The signal is stationary from the outset, and the characteristic diffusion time defined by the change-rate criterion is zero.

More generally, signals of the form $x_i = c_r n_i$ within component V_r are stationary even when the constants c_r differ between components. Such contrasts belong to the stationary subspace and cannot be recovered by merely discarding the additional zero eigenmodes. Therefore, the current SpatioCAD formulation requires a connected graph for global diffusion-based ranking. The implementation checks connectivity before scoring and does not silently process disconnected inputs.

Supplementary Tables

Table A. Hardware configuration for computational analyses

Component	Specification
CPU	Intel Xeon Gold 6226R (2.90 GHz, 16 cores per job)
GPU	NVIDIA A30 (24GB GDDR6, CUDA 12.2)
Memory	1TB total RAM with dynamic allocation
Storage	High-speed SSD
OS & Scheduler	Ubuntu 22.04.4 LTS with SLURM

Table B. Gene filtering by roughness score (RS) in SpatioCAD across real datasets

Dataset / slice	Genes entering RS after common QC	RS-removed genes	Removed (%)	Genes retained after RS
Human breast cancer	17,633	567	3.22	17,066
Human lung cancer	17,269	443	2.57	16,826
GSE194329 / DMG1	15,448	439	2.84	15,009
GSE194329 / GBM3	12,833	390	3.04	12,443
GSE194329 / GBM4	13,862	627	4.52	13,235
DLPFC / 151507	9,826	450	4.58	9,376
DLPFC / 151508	9,133	569	6.23	8,564
DLPFC / 151509	10,389	633	6.09	9,756
DLPFC / 151510	9,958	414	4.16	9,544
DLPFC / 151669	10,294	512	4.97	9,782
DLPFC / 151670	9,848	433	4.40	9,415
DLPFC / 151671	10,907	493	4.52	10,414
DLPFC / 151672	10,412	595	5.71	9,817
DLPFC / 151673	11,270	496	4.40	10,774
DLPFC / 151674	12,262	447	3.65	11,815
DLPFC / 151675	10,510	410	3.90	10,100
DLPFC / 151676	10,652	575	5.40	10,077
