# Supplementary material for: SpatioCAD: Context-aware graph diffusion model for pinpointing spatially variable genes in heterogeneous tissues
Source: PLoS Comput Biol. 2026 Nov 2;22(11):e1014923. doi: 10.1371/journal.pcbi.1014923 (PMC13750662; doi:10.1371/journal.pcbi.1014923)
Supplement: Supplementary file 2 [file pcbi.1014923.s002.pdf]

S2 Appendix

Supplementary figures (Part I)

Supporting information for
"SpatioCAD: Context-aware graph diffusion model for pinpointing spatially variable genes in heterogeneous tissues"

Supplementary Figures

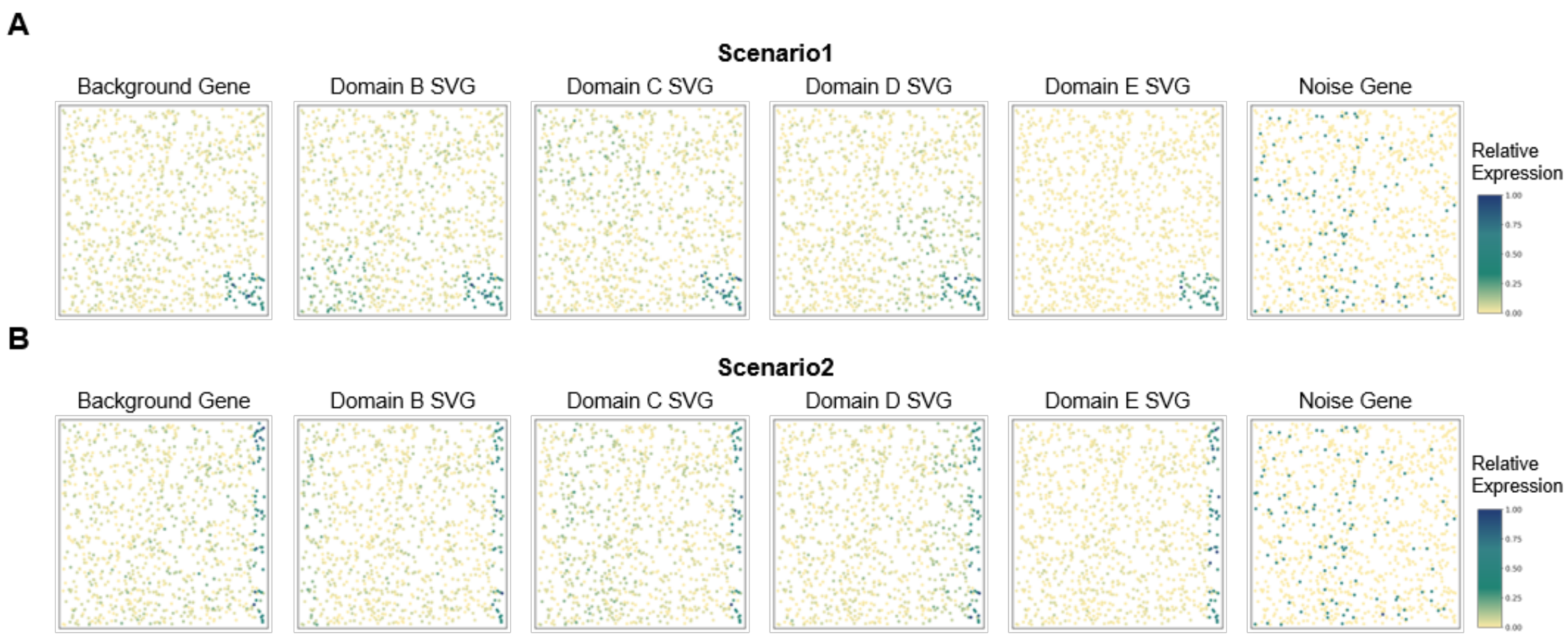

Fig A. (A) Representative spatial expression patterns in Scenario 1 for background genes, SVGs specific to domains B–E, and noise genes. (B) Representative spatial expression patterns in Scenario 2 for background genes, SVGs specific to domains B–E, and noise genes.

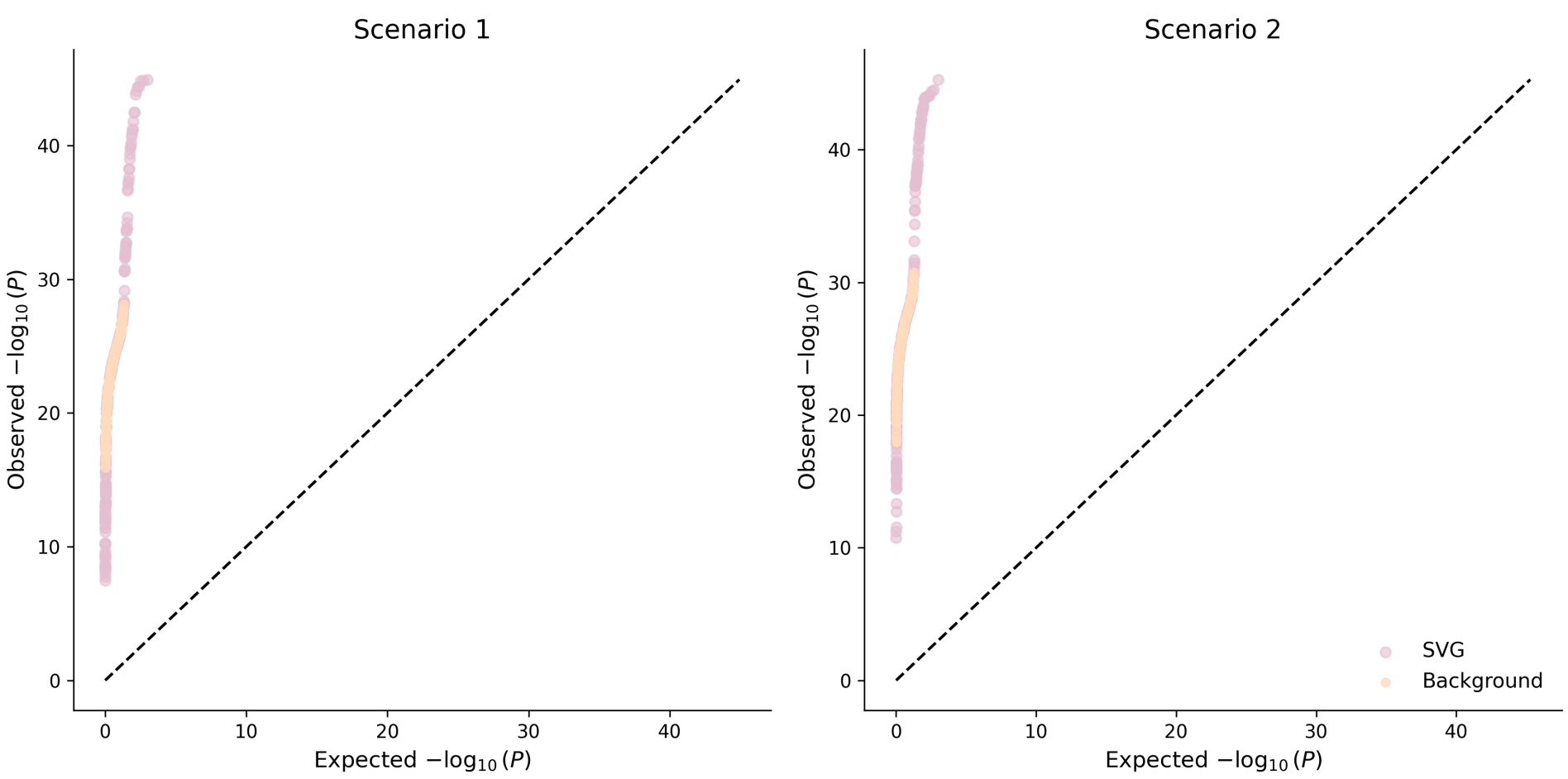

Fig B. Quantile-quantile plots of the observed $-\log_{10}(p)$ against the expected $-\log_{10}(p)$ of SPARKX in two simulated scenarios.

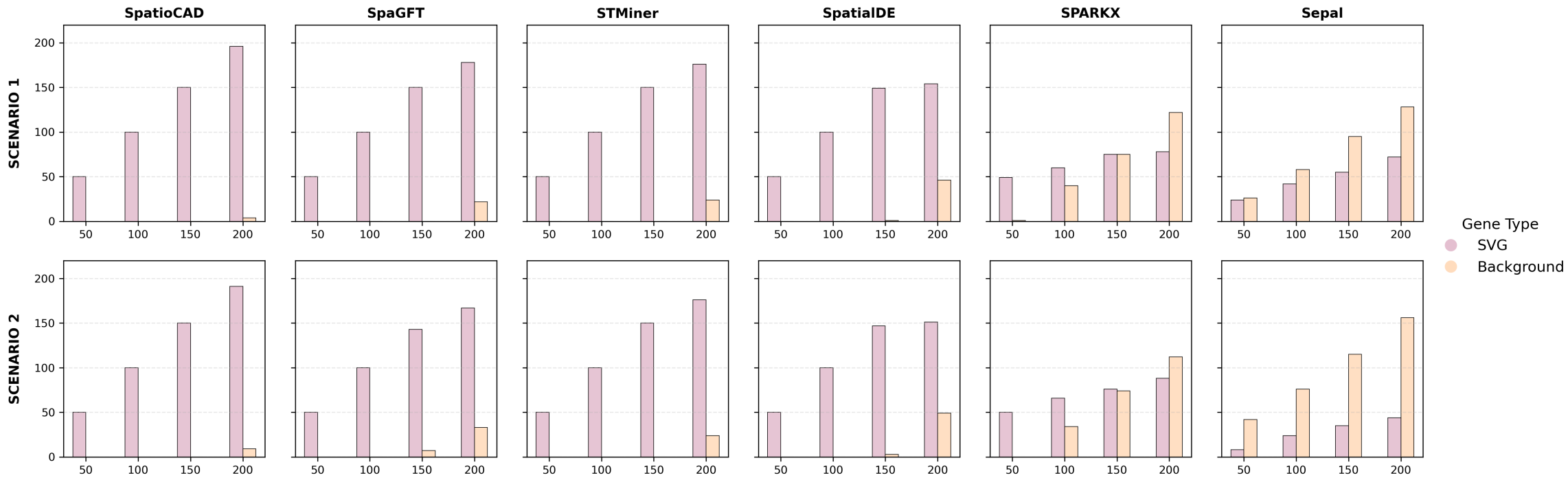

Fig C. Composition of gene types within the top-ranked genes identified by six methods in noise-free simulations. The bar plots display the counts of ground-truth SVGs (pink) and background genes (orange) among the top N genes ($N = 50, 100, 150, 200$) ranked by each method. Results are shown for Scenario 1 (top row) and Scenario 2 (bottom row).

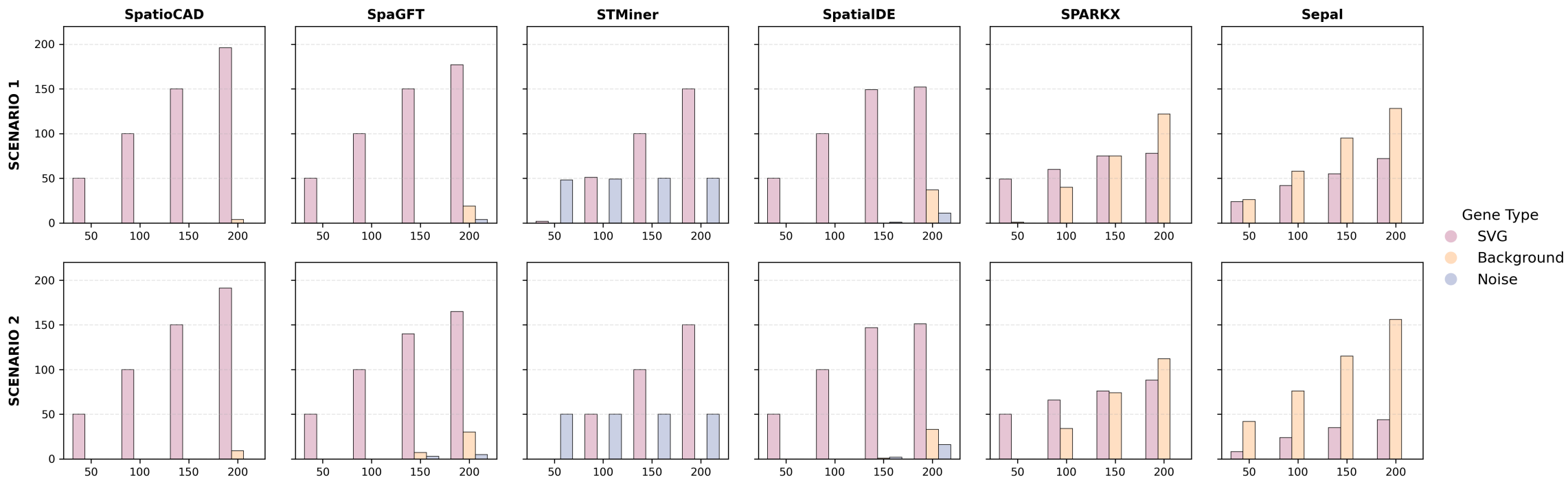

Fig D. Composition of gene types within the top-ranked genes identified by six methods in simulations with noise genes. The bar plots display the counts of ground-truth SVGs (pink), background genes (orange), and noise genes (blue) among the top N genes ($N = 50, 100, 150, 200$) ranked by each method. Results are shown for Scenario 1 (top row) and Scenario 2 (bottom row).

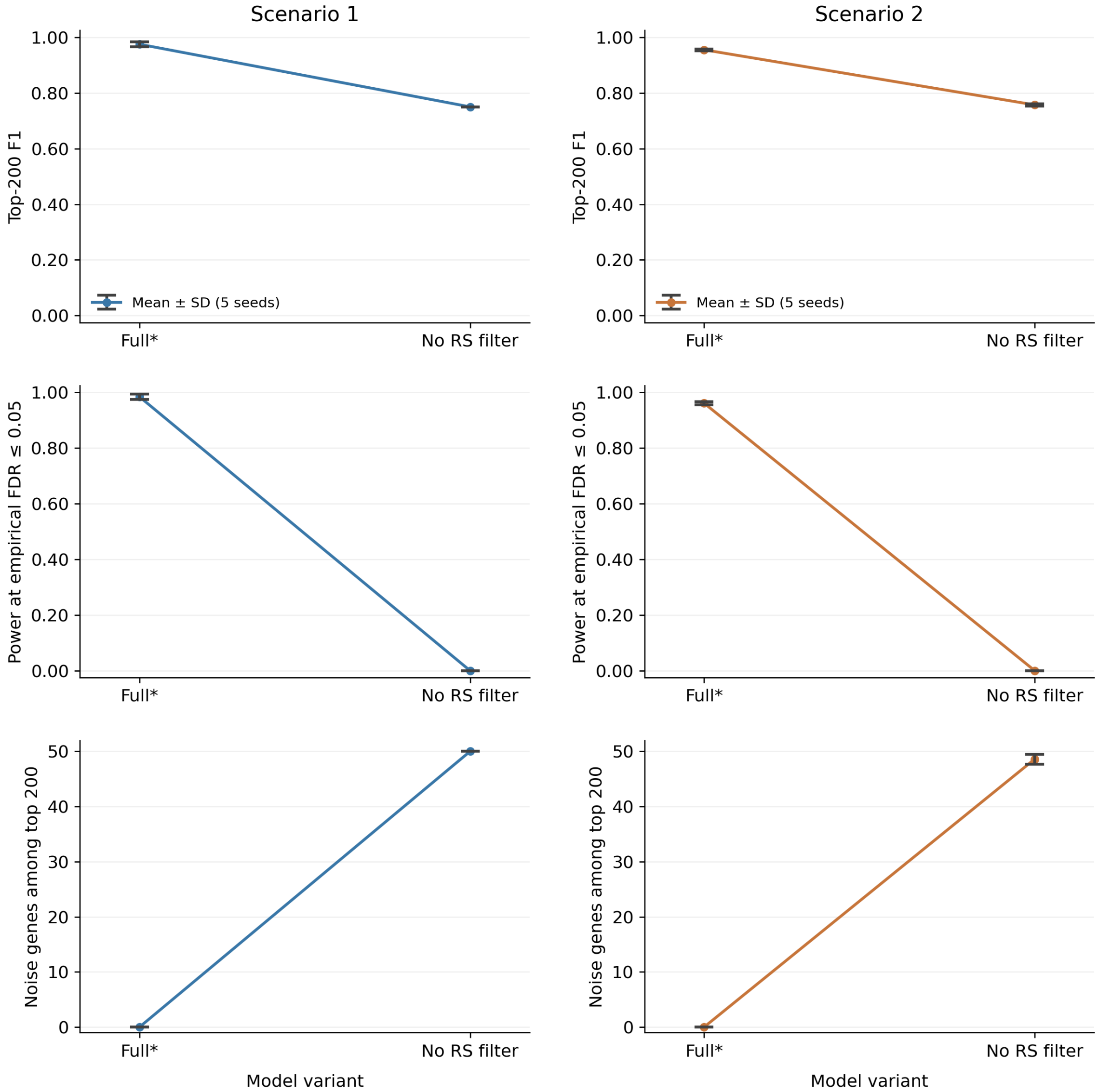

Fig E. Contribution of Roughness Score-based noise filtering. The full SpatioCAD pipeline was compared with a variant without RS-based noise filtering in simulated datasets containing noise genes. Columns show Scenarios 1 and 2. Rows show the Top-200 F1 score, power at the empirical FDR threshold of 0.05, and the number of simulated noise genes among the top 200 ranked genes, respectively. Points and error bars indicate the mean and sample standard deviation across five simulation replicates per scenario. The full pipeline is marked with an asterisk as the reference model.

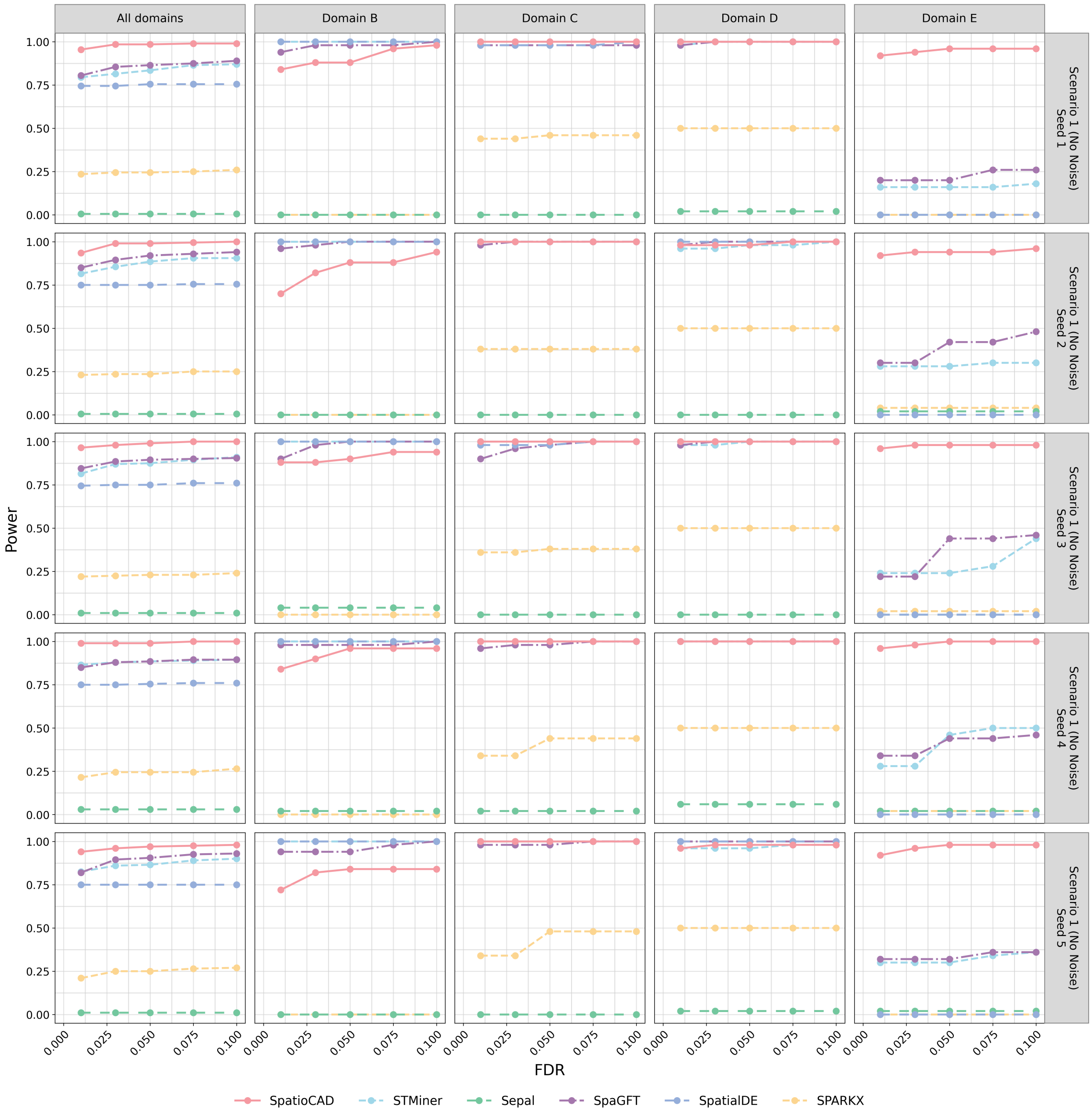

Fig F. Performance comparison across five random seeds in Scenario 1 without noise genes. Power plots show the statistical power (y-axis) detected by different methods (represented by different colors and line types) at a range of FDR (x-axis) across all domains and the four separate domains (columns) and the five random seeds (rows).

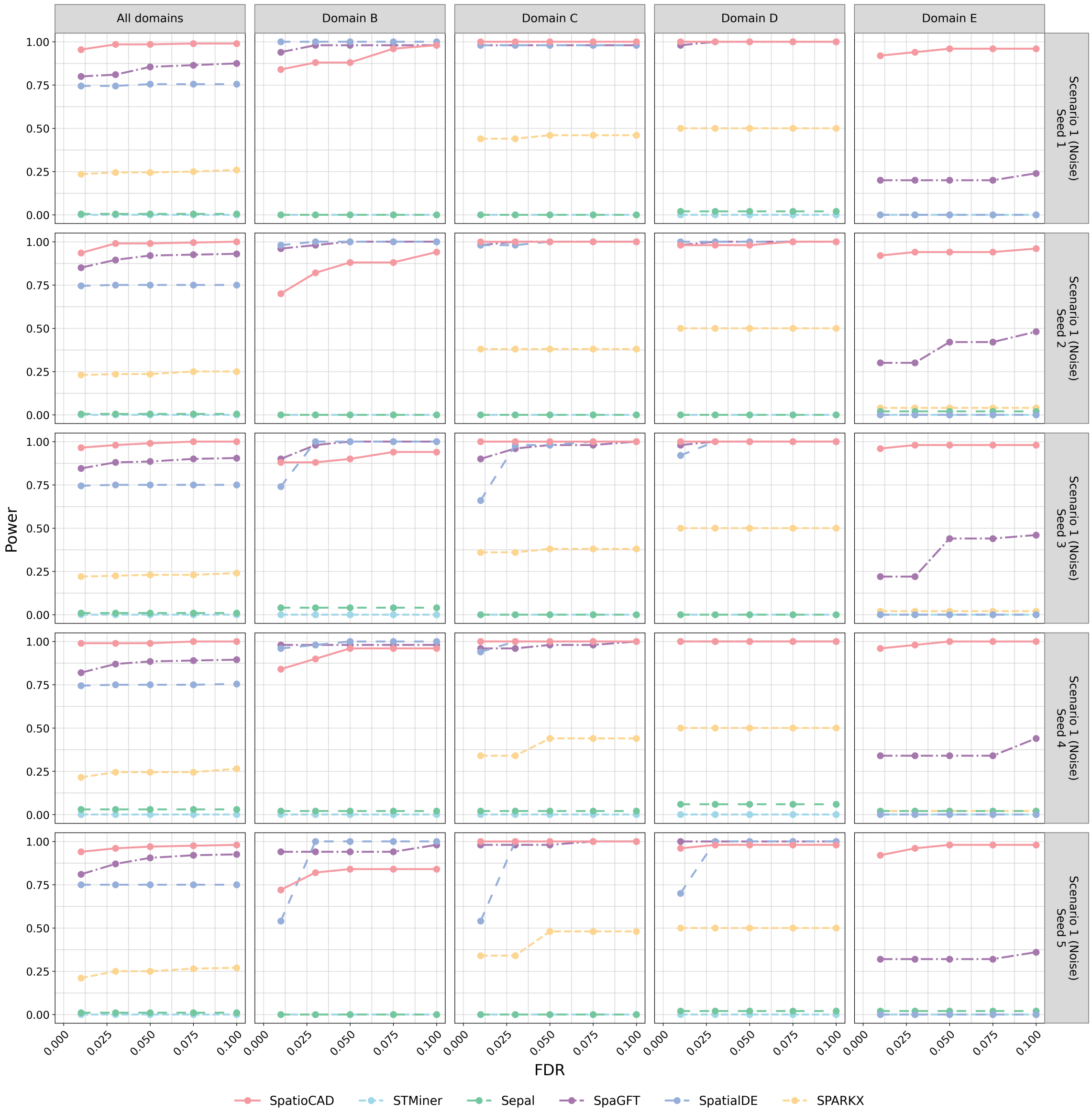

Fig G. Performance comparison across five random seeds in Scenario 1 with noise genes. Power plots show the statistical power (y-axis) detected by different methods (represented by different colors and line types) at a range of FDR (x-axis) across all domains and the four separate domains (columns) and the five random seeds (rows).

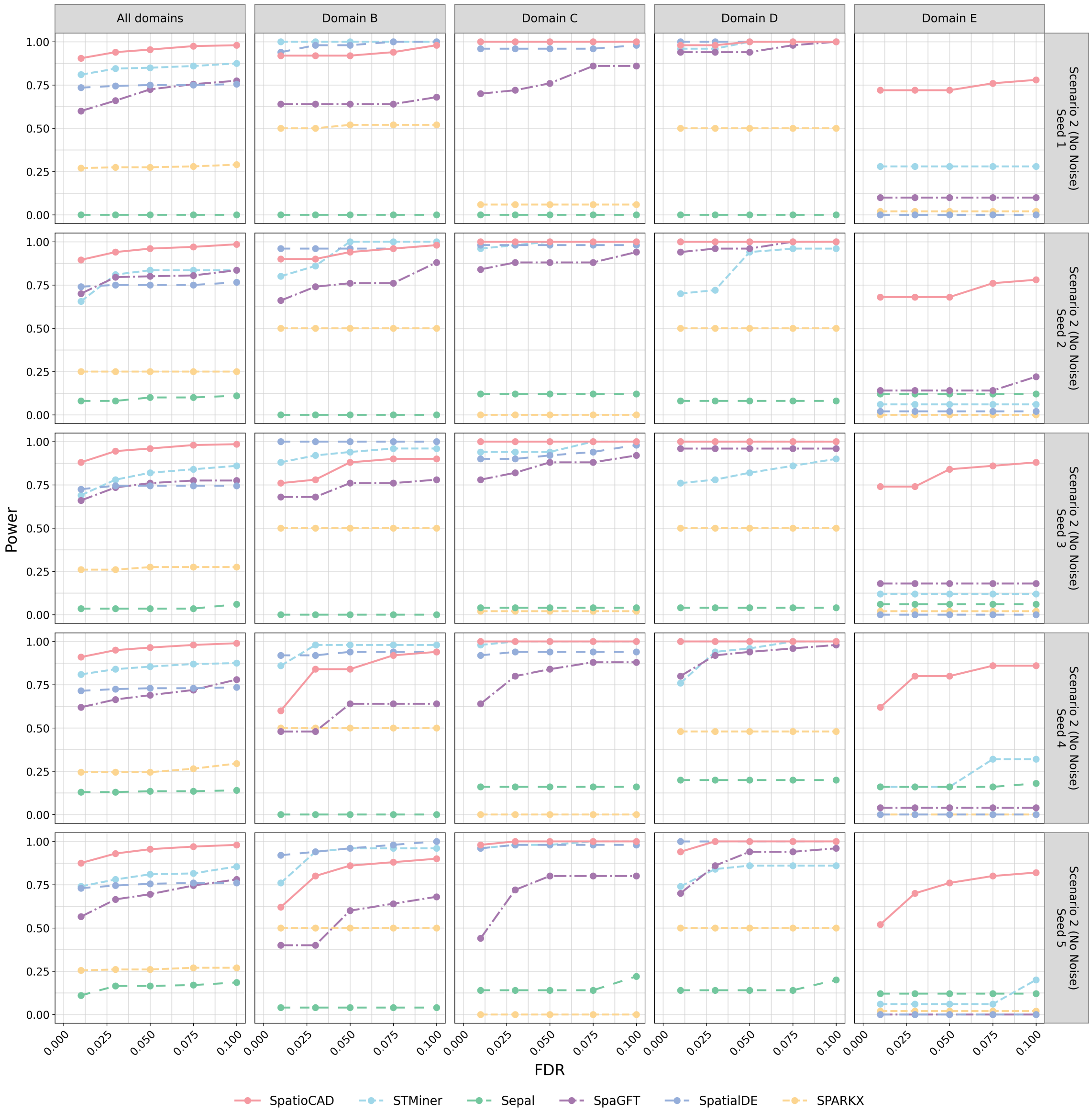

Fig H. Performance comparison across five random seeds in Scenario 2 without noise genes. Power plots show the statistical power (y-axis) detected by different methods (represented by different colors and line types) at a range of FDR (x-axis) across all domains and the four separate domains (columns) and the five random seeds (rows).

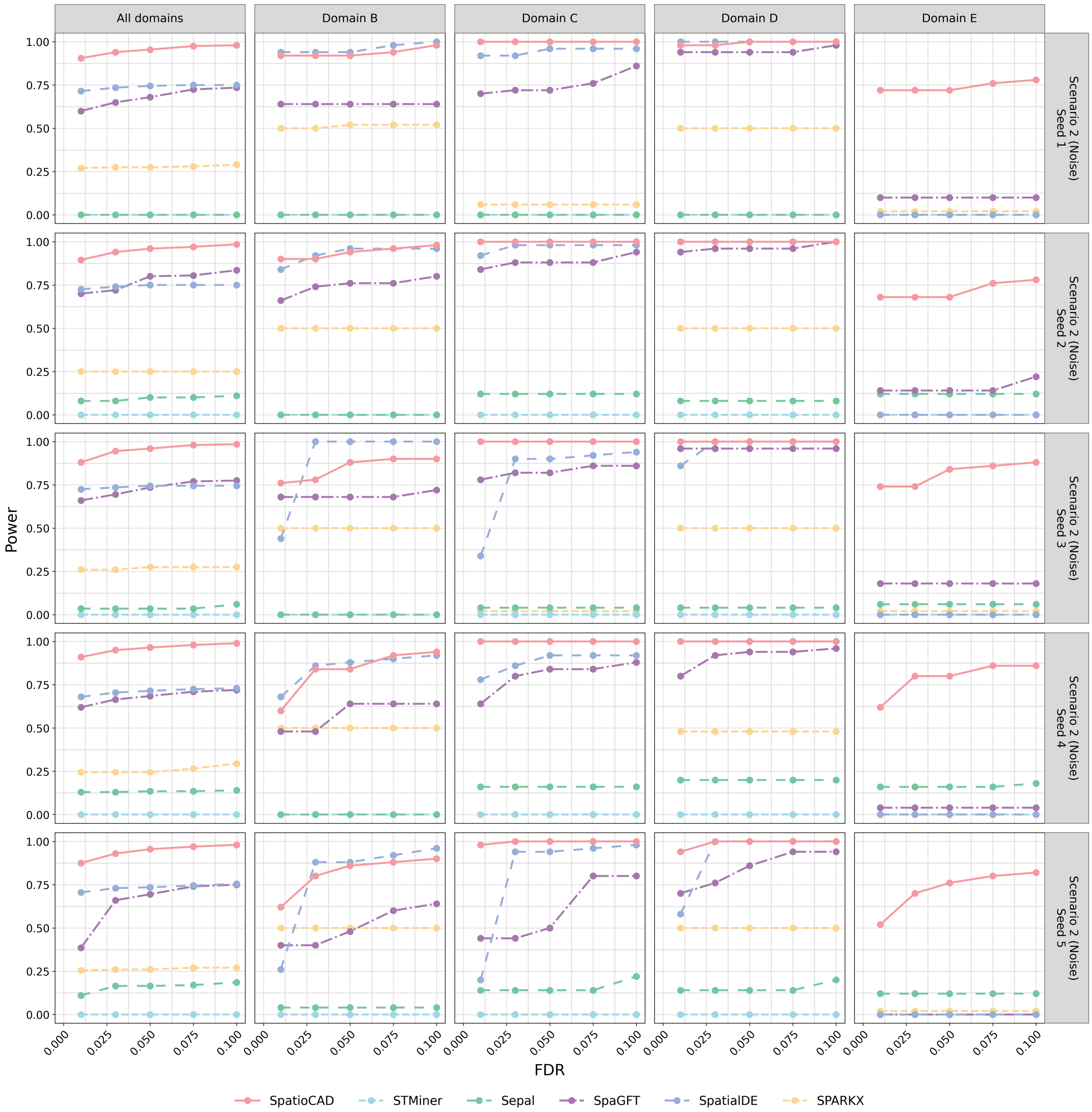

Fig I. Performance comparison across five random seeds in Scenario 2 with noise genes. Power plots show the statistical power (y-axis) detected by different methods (represented by different colors and line types) at a range of FDR (x-axis) across all domains and the four separate domains (columns) and the five random seeds (rows).

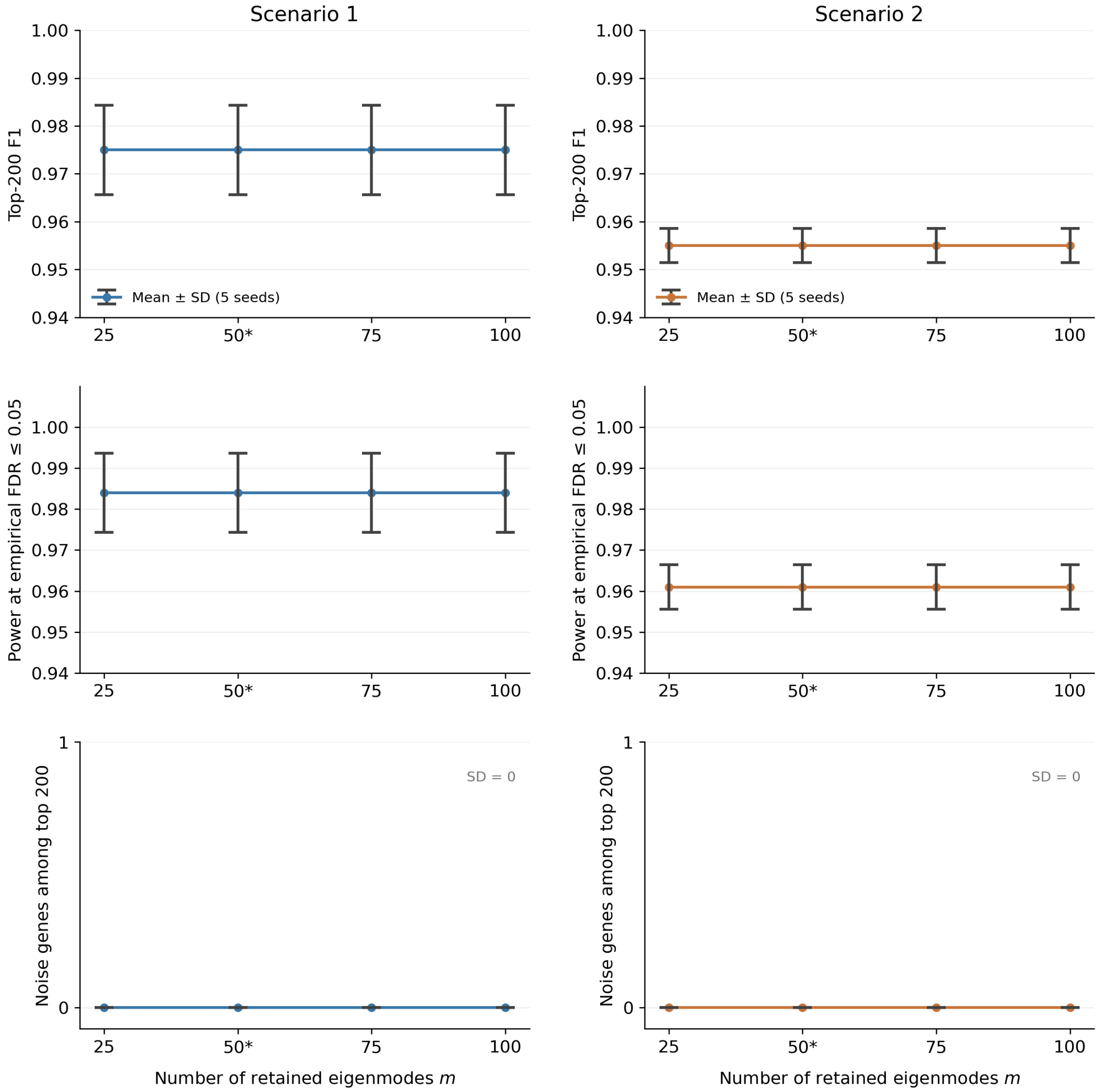

Fig J. Sensitivity to the number of retained eigenmodes. The number of retained nonzero eigenmodes was varied over $m \in \{25, 50, 75, 100\}$, with all other settings held fixed. Analyses were performed on simulated datasets containing noise genes. Columns show Scenarios 1 and 2. Rows show the Top-200 F1 score, power at the empirical FDR threshold of 0.05, and the number of simulated noise genes among the top 200 ranked genes, respectively. Points and error bars indicate the mean and sample standard deviation across five simulation replicates per scenario. The reference value $m = 50$ is marked with an asterisk.

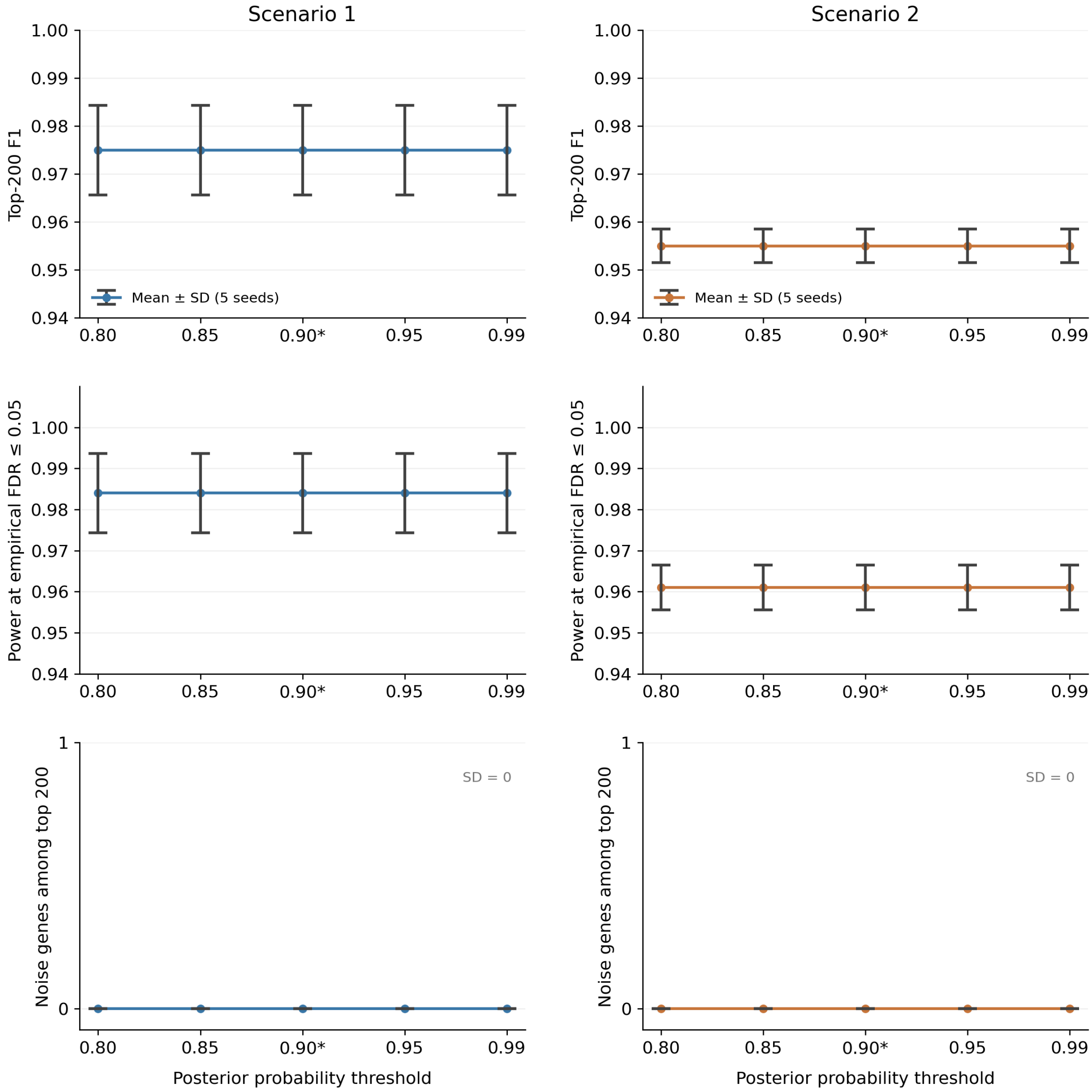

Fig K. Sensitivity to the posterior probability threshold for RS-based filtering. The posterior probability threshold was varied over $\tau \in \{0.80, 0.85, 0.90, 0.95, 0.99\}$. Genes were excluded when their posterior probability of belonging to the GMM component with the highest mean RS exceeded τ. Analyses were performed on simulated datasets containing noise genes. Columns show Scenarios 1 and 2. Rows show the Top-200 F1 score, power at the empirical FDR threshold of 0.05, and the number of simulated noise genes among the top 200 ranked genes, respectively. Points and error bars indicate the mean and sample standard deviation across five simulation replicates per scenario. The reference value $\tau = 0.90$ is marked with an asterisk.

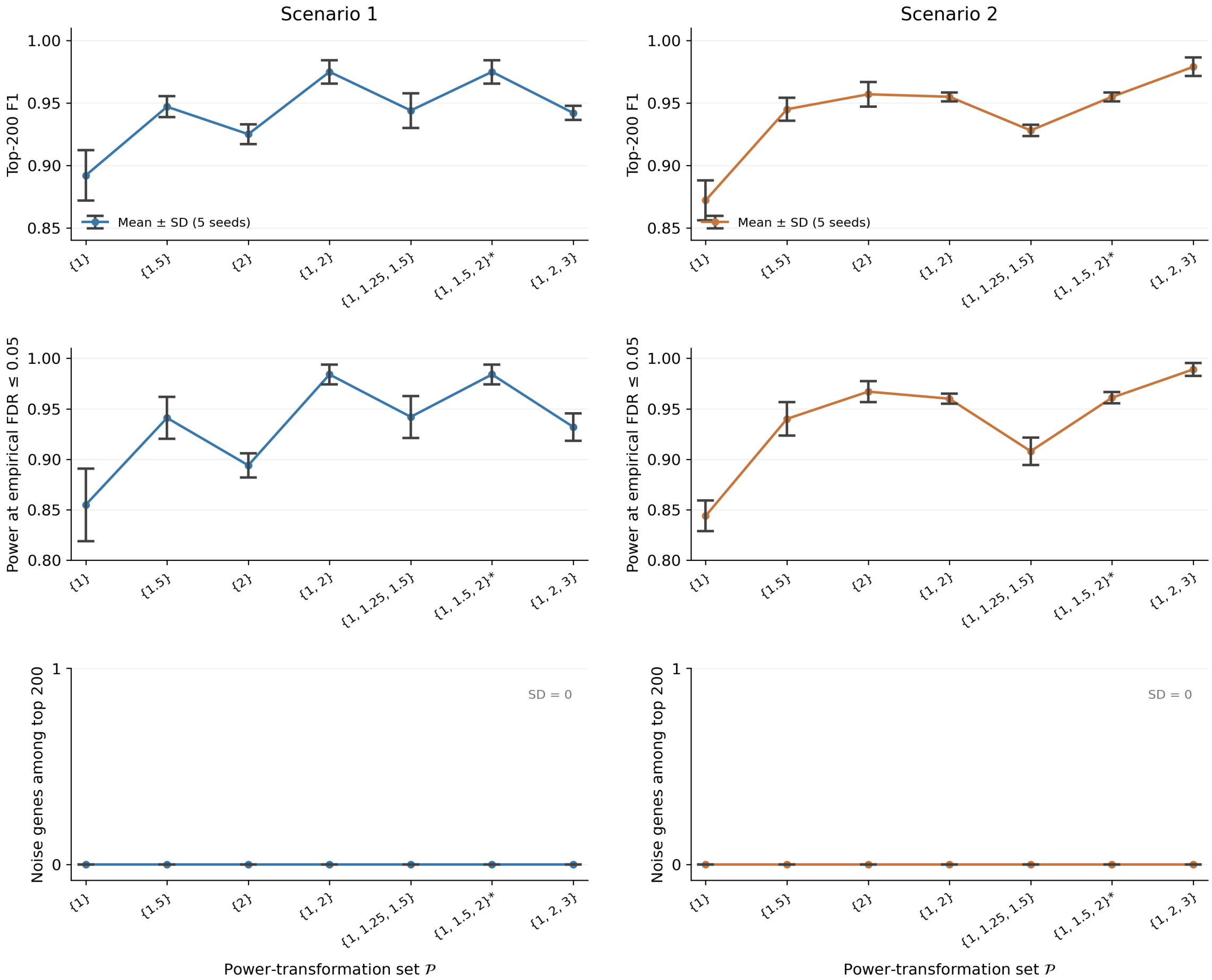

Fig L. Sensitivity to the power-transformation set. Seven power-transformation sets were evaluated: $\{1\}$, $\{1.5\}$, $\{2\}$, $\{1, 2\}$, $\{1, 1.25, 1.5\}$, $\{1, 1.5, 2\}$, and $\{1, 2, 3\}$. Each set specifies the exponents applied before diffusion-time calculation and rank aggregation. Analyses were performed on simulated datasets containing noise genes. Columns show Scenarios 1 and 2. Rows show the Top-200 F1 score, power at the empirical FDR threshold of 0.05, and the number of simulated noise genes among the top 200 ranked genes, respectively. Points and error bars indicate the mean and sample standard deviation across five simulation replicates per scenario. The reference set $\mathcal{P} = \{1, 1.5, 2\}$ is marked with an asterisk.

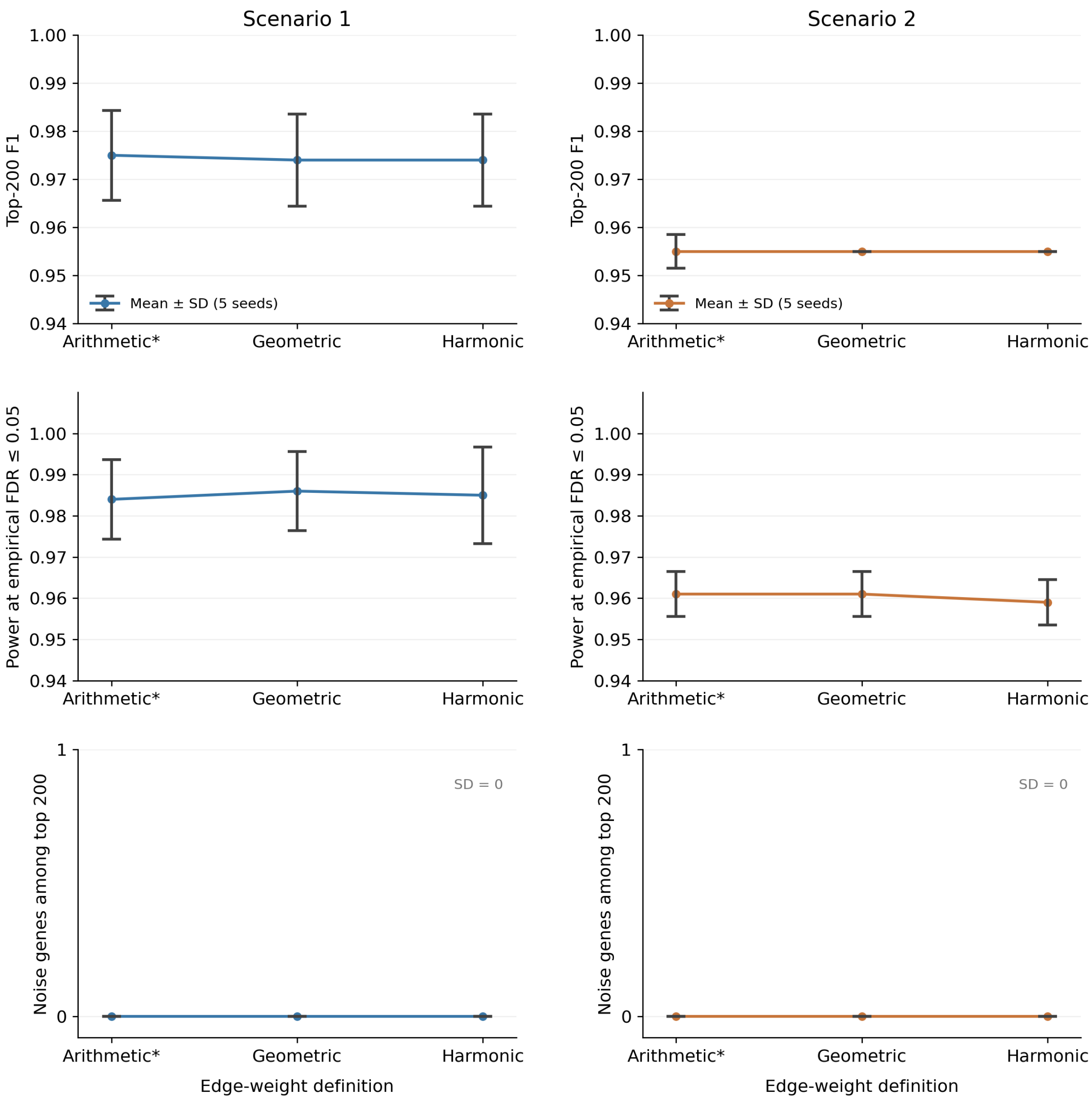

Fig M. Sensitivity to the density-dependent edge-weight definition. Three definitions of $w_{i,j}$ were compared: the arithmetic mean, $(n_i + n_j)/2$; the geometric mean, $\sqrt{n_i n_j}$; and the harmonic mean, $2n_i n_j/(n_i + n_j)$. The spatial adjacency matrix, density proxy, and density normalization were unchanged. Analyses were performed on simulated datasets containing noise genes. Columns show Scenarios 1 and 2. Rows show the Top-200 F1 score, power at the empirical FDR threshold of 0.05, and the number of simulated noise genes among the top 200 ranked genes, respectively. Points and error bars indicate the mean and sample standard deviation across five simulation replicates per scenario. The reference definition arithmetic mean is marked with an asterisk.

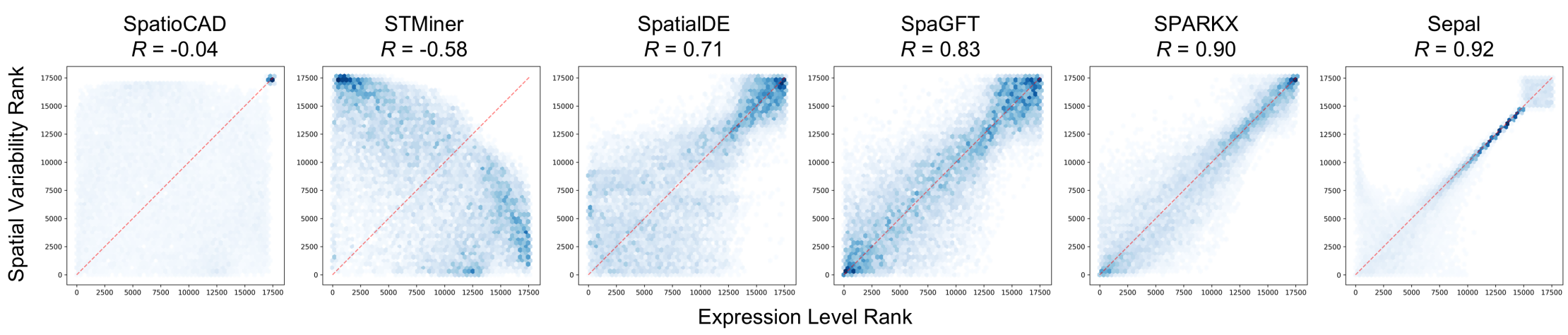

Fig N. Hexbin density plots show the relationship between gene expression ranks (x-axis) and spatial variability ranks (y-axis) in human breast cancer data. Spearman correlation between the two ranks was calculated for each method (SpatioCAD: -0.04, STMiner: -0.58, SpatialDE: 0.71, SpaGFT: 0.83, SPARK-X: 0.90, Sepal: 0.92).

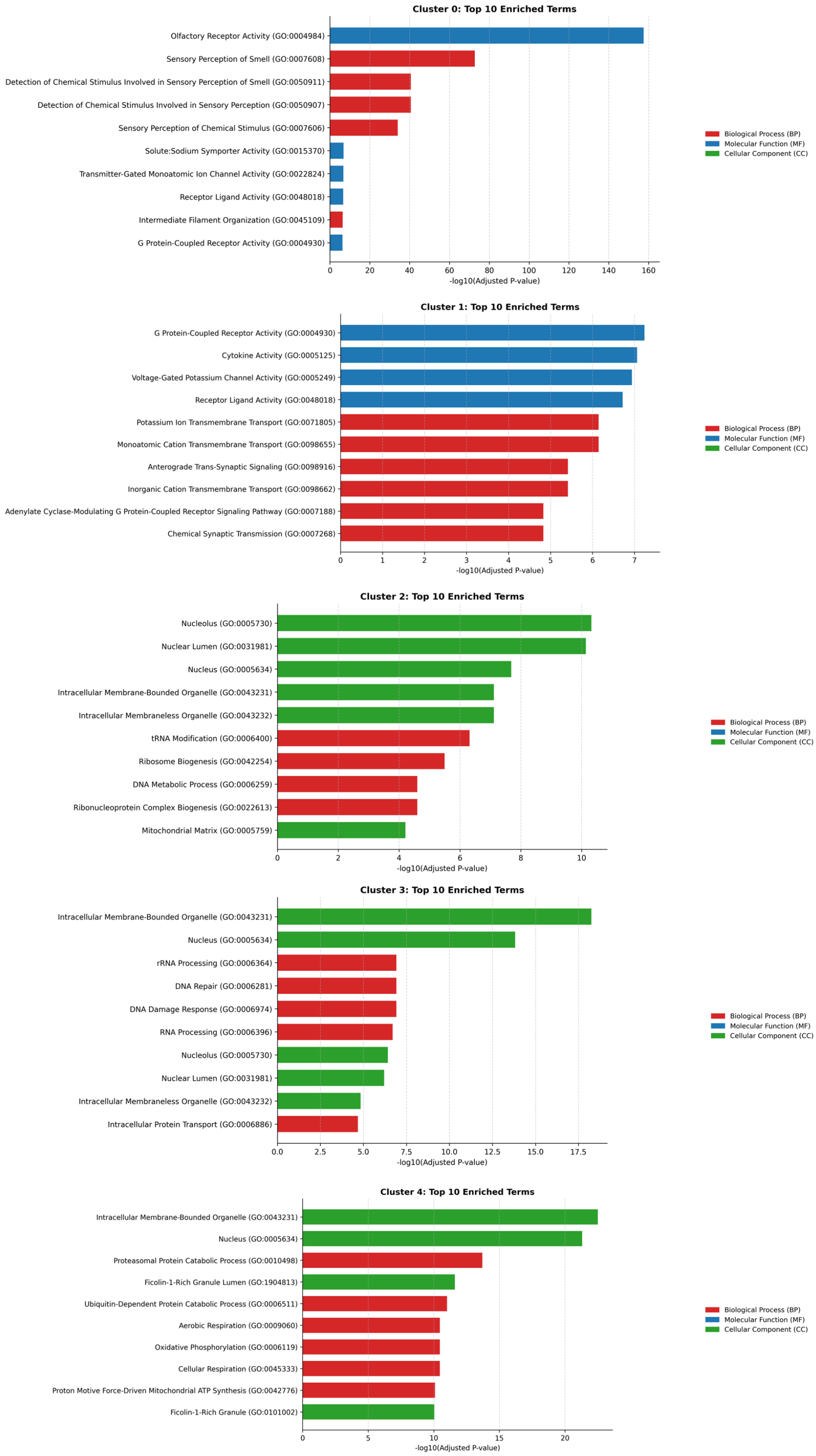

Fig O. Significantly enriched gene sets (FDR adjusted p-value < 0.05) in gene set enrichment results for Cluster 0-4 in human breast cancer data on three reference gene sets: GO biological process, GO molecular function, and GO cellular component.

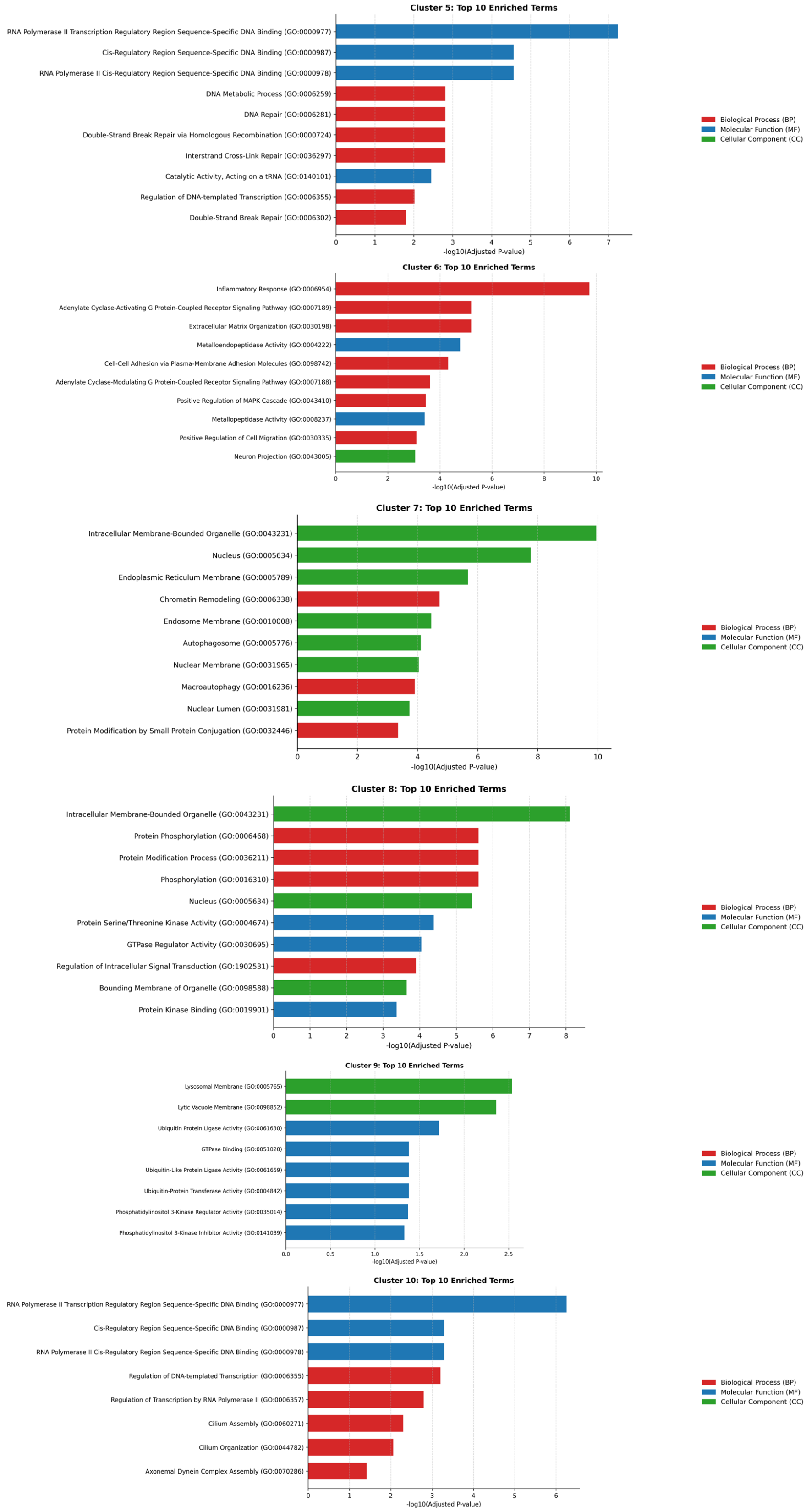

Fig P. Significantly enriched gene sets (FDR adjusted p-value < 0.05) in gene set enrichment results for Cluster 5-10 in human breast cancer data on three reference gene sets: GO biological process, GO molecular function, and GO cellular component.

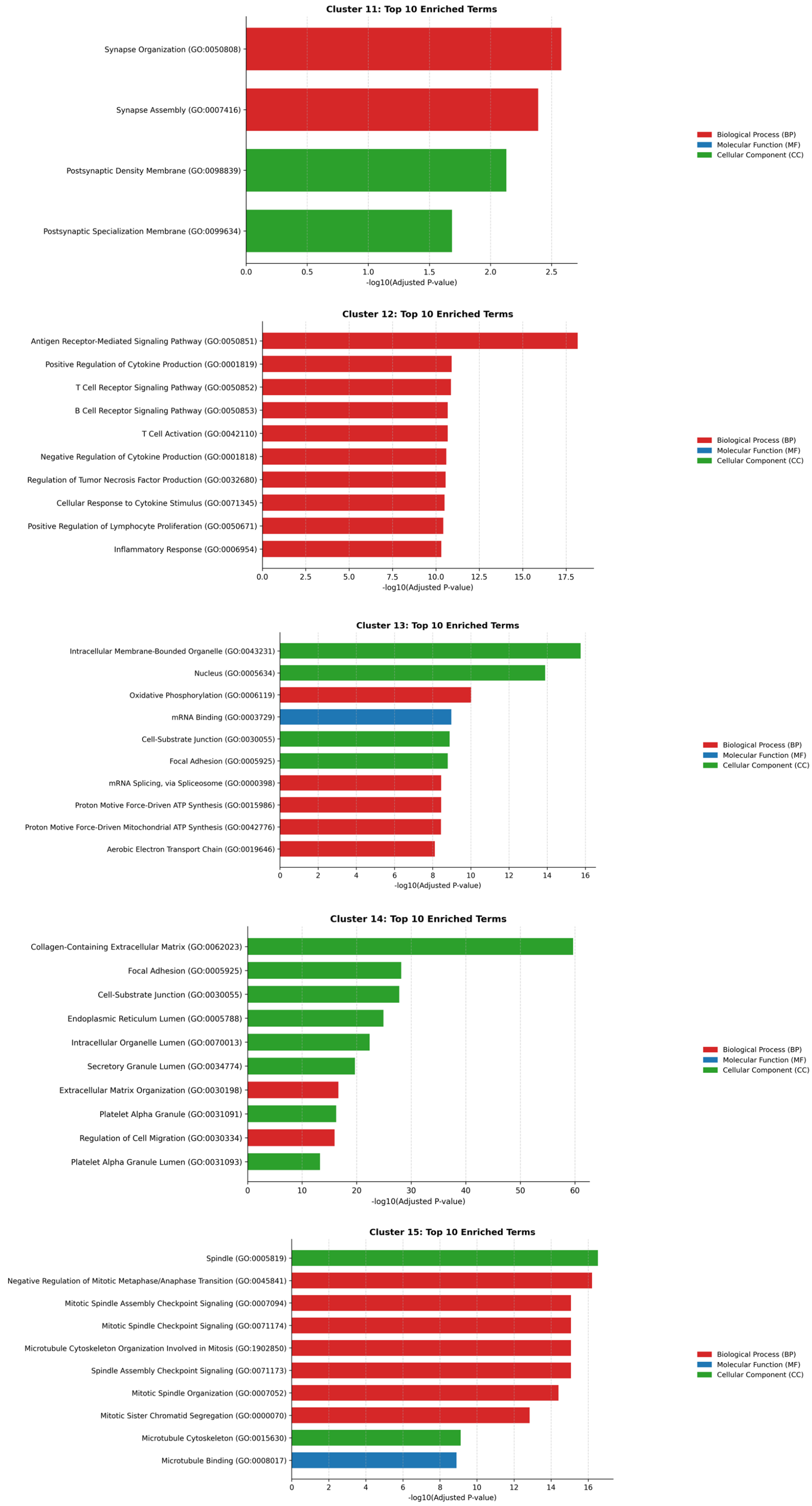

Fig Q. Significantly enriched gene sets (FDR adjusted p-value < 0.05) in gene set enrichment results for Cluster 11-15 in human breast cancer data on three reference gene sets: GO biological process, GO molecular function, and GO cellular component.

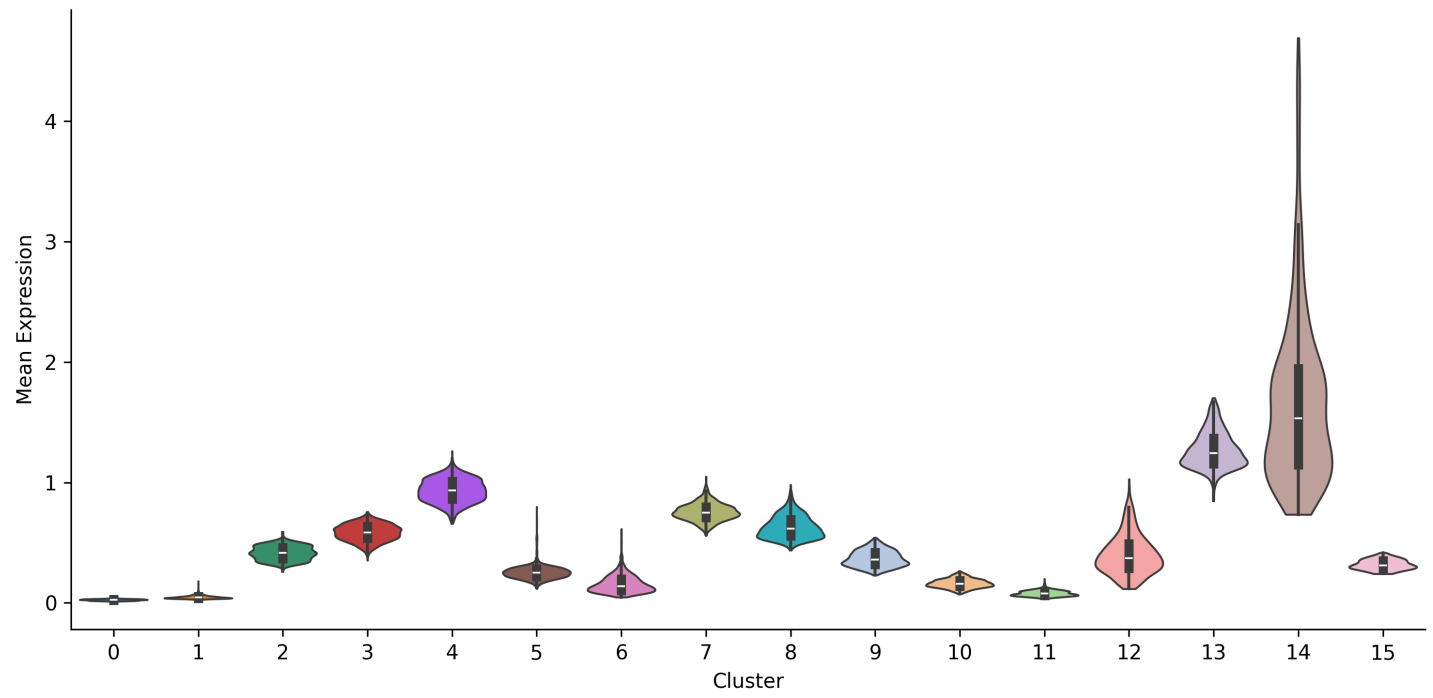

Fig R. Distribution of mean gene expression levels across identified clusters in human breast cancer data. The violin plots visualize the density and range of mean expression values for each cluster.

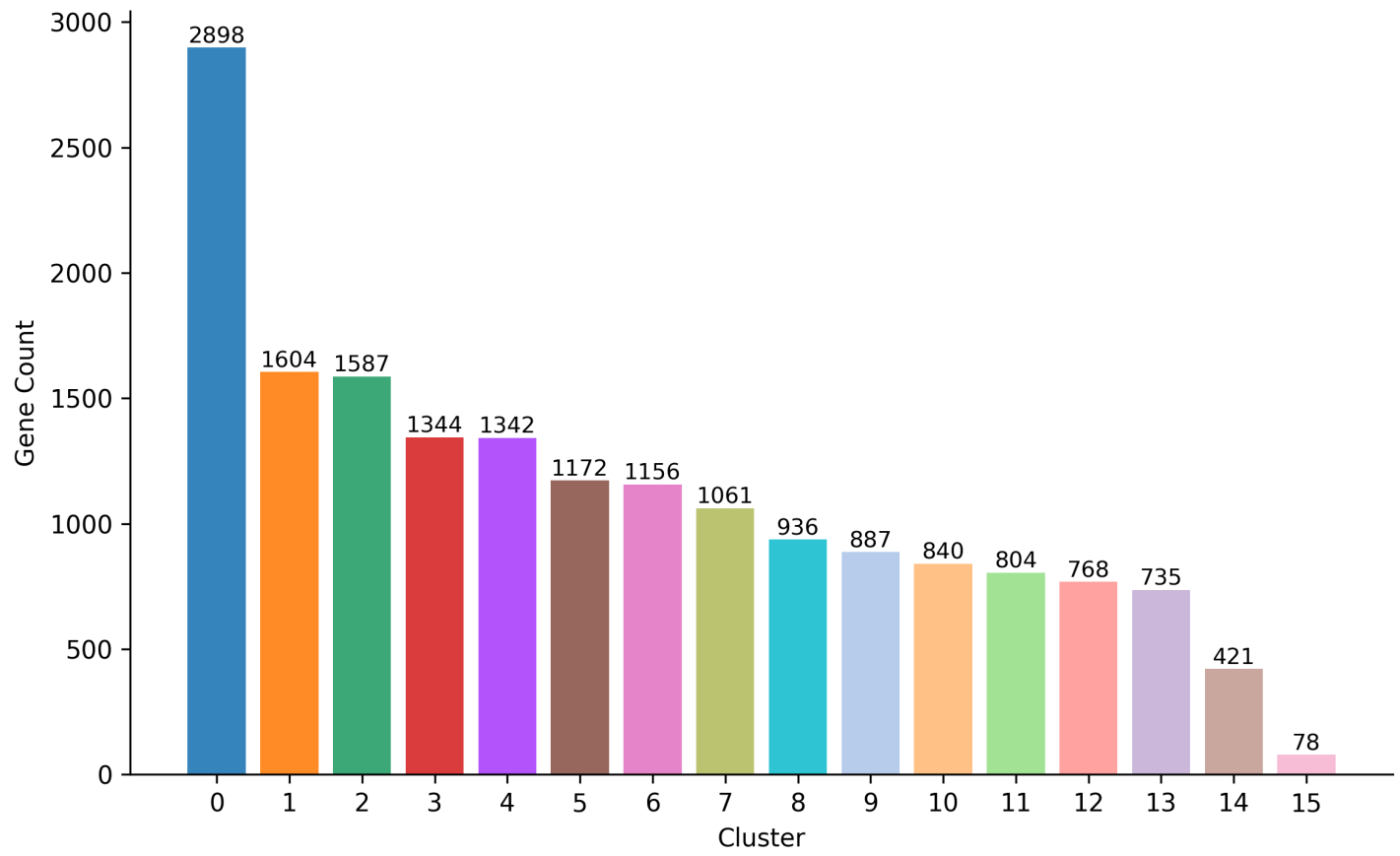

Fig S. Number of genes assigned to each identified cluster in human breast cancer data. The bar plot displays the total count of genes classified into each cluster.

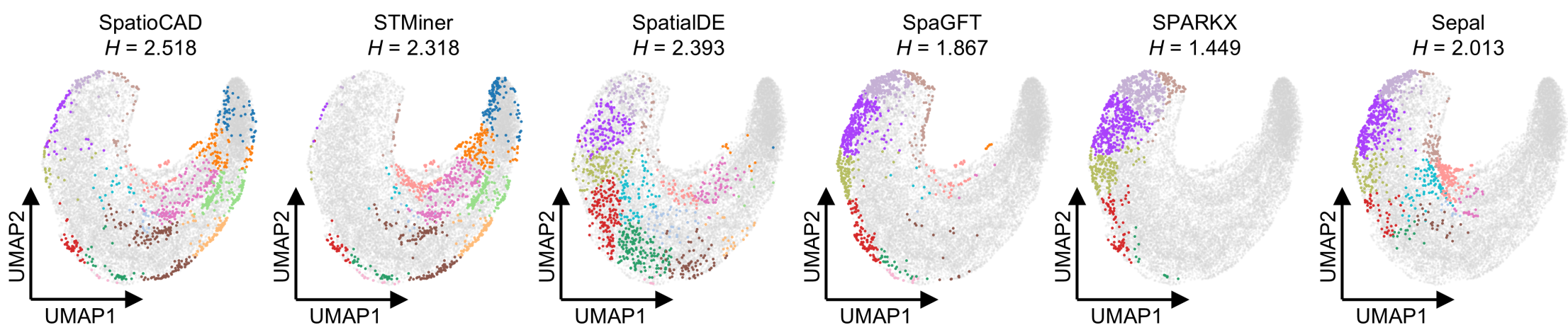

Fig T. Projection of the top 1,000 SVGs identified by each method onto the global gene UMAP in human breast cancer data. Shannon entropy of their distribution across these clusters was calculated for each method (SpatioCAD: 2.518, STMiner: 2.318, SpatialDE: 2.393, SpaGFT: 1.867, SPARK-X: 1.449, Sepal: 2.013).

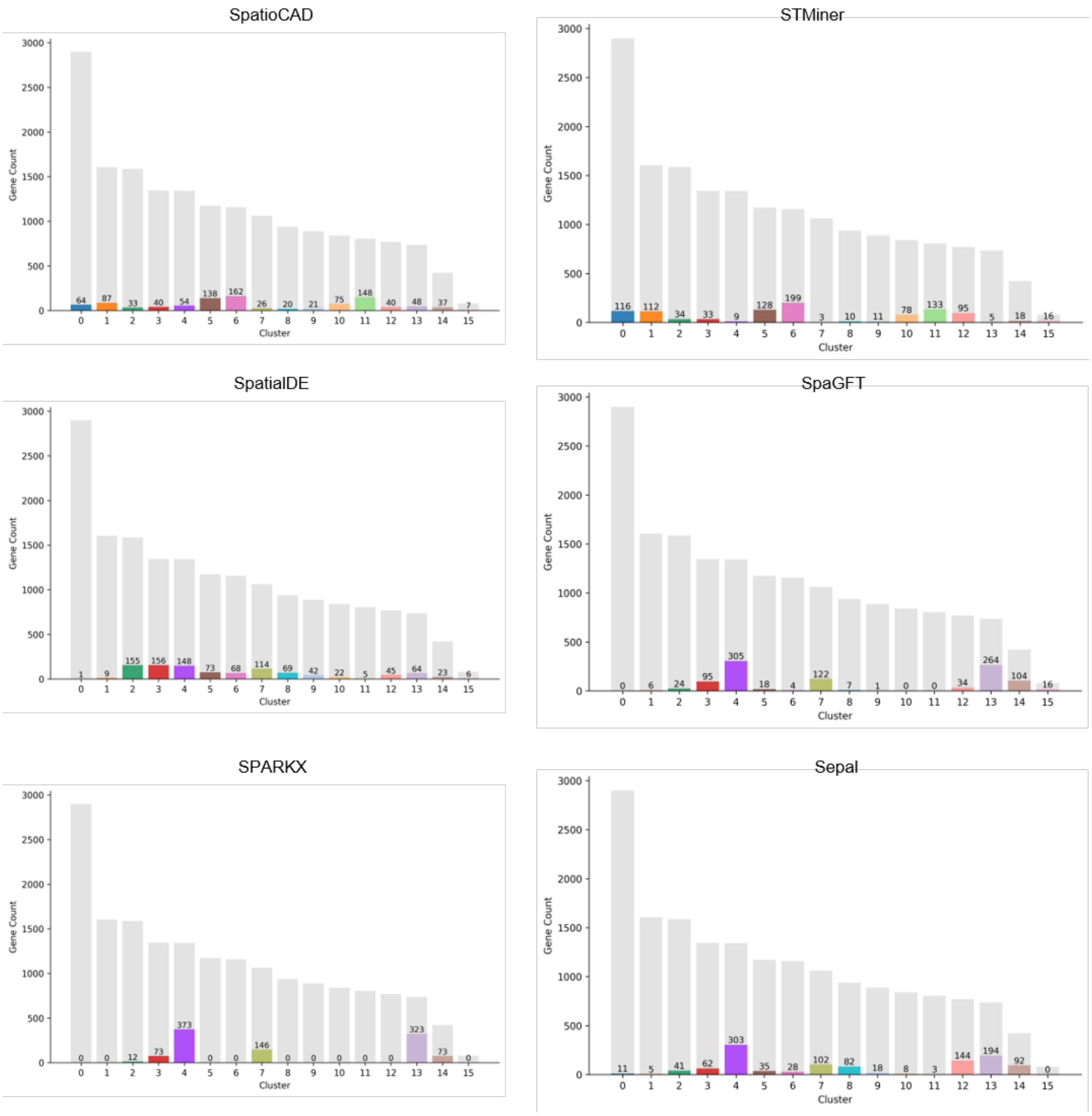

Fig U. Cluster detection preference of six methods in human breast cancer data. Each panel displays the number of genes identified by a specific method across the 16 defined clusters. The light grey bars represent the total size of each gene cluster (background). The colored bars and the numbers above them indicate the count of genes within each cluster that were identified by the respective method.

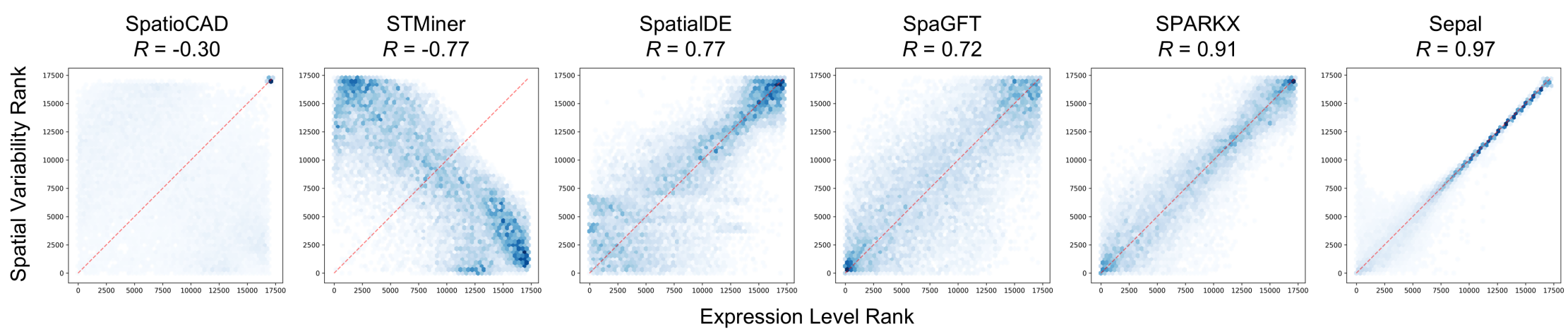

Fig V. Hexbin density plots show the relationship between gene expression ranks (x-axis) and spatial variability ranks (y-axis) in human lung cancer data. Spearman correlation between the two ranks was calculated for each method (SpatioCAD: −0.30, STMiner: −0.77, SpatialDE: 0.77, SpaGFT: 0.72, SPARK-X: 0.91, Sepal: 0.97).

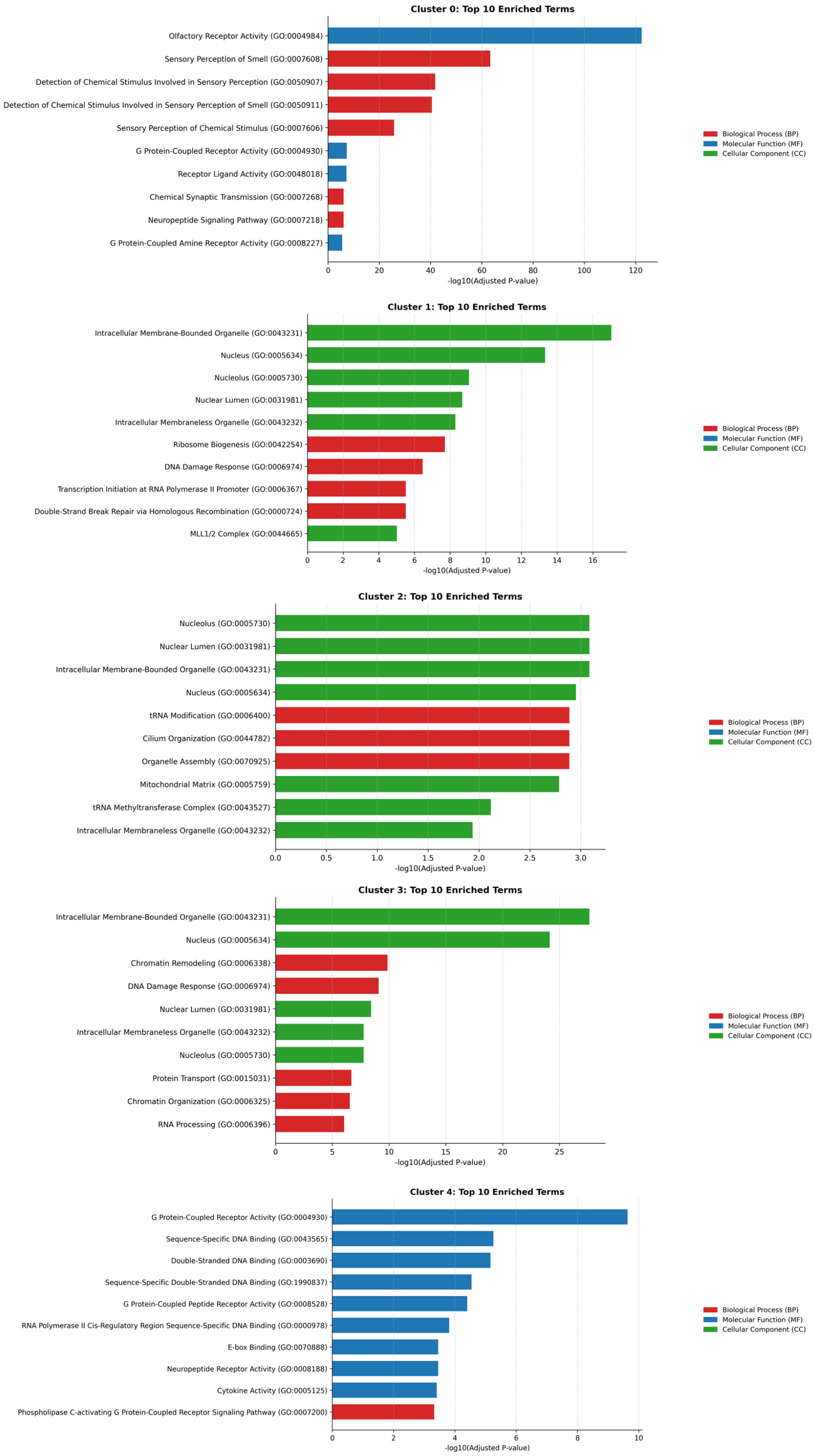

Fig W. Significantly enriched gene sets (FDR adjusted p-value < 0.05) in gene set enrichment results for Cluster 0-4 in human lung cancer data on three reference gene sets: GO biological process, GO molecular function, and GO cellular component.

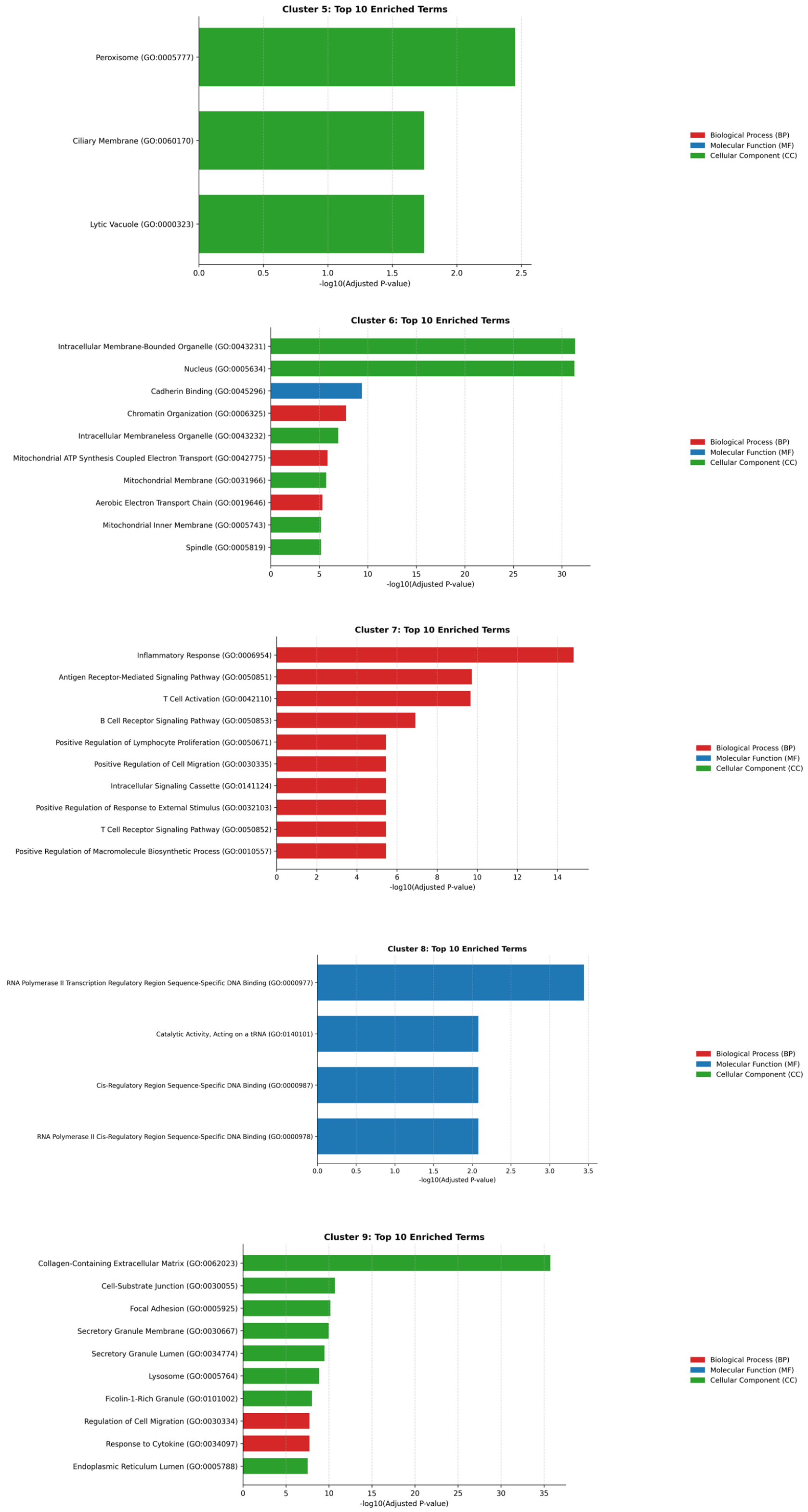

Fig X. Significantly enriched gene sets (FDR adjusted p-value < 0.05) in gene set enrichment results for Cluster 5-9 in human lung cancer data on three reference gene sets: GO biological process, GO molecular function, and GO cellular component.

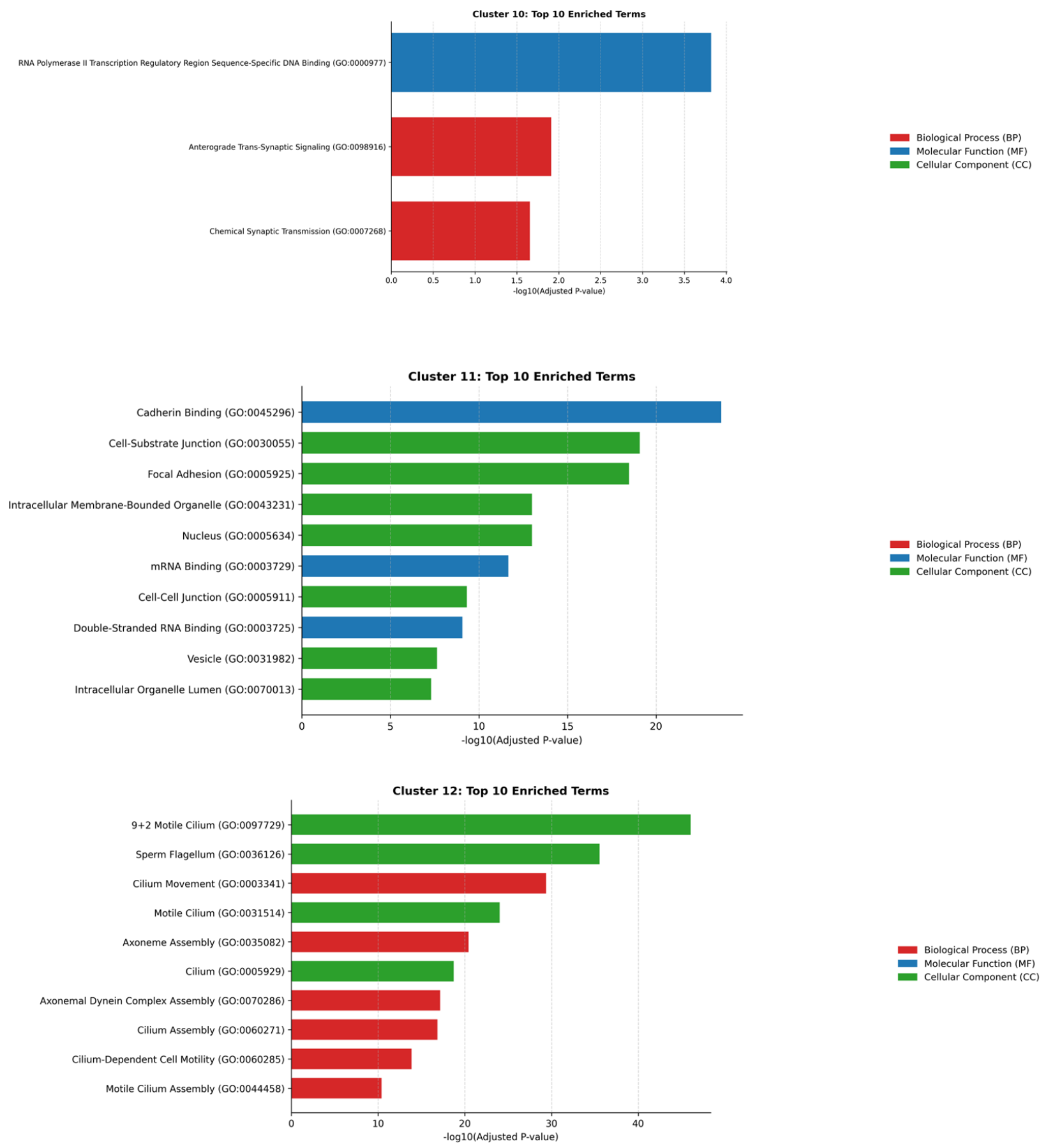

Fig Y. Significantly enriched gene sets (FDR adjusted p-value < 0.05) in gene set enrichment results for Cluster 10-12 in human lung cancer data on three reference gene sets: GO biological process, GO molecular function, and GO cellular component.

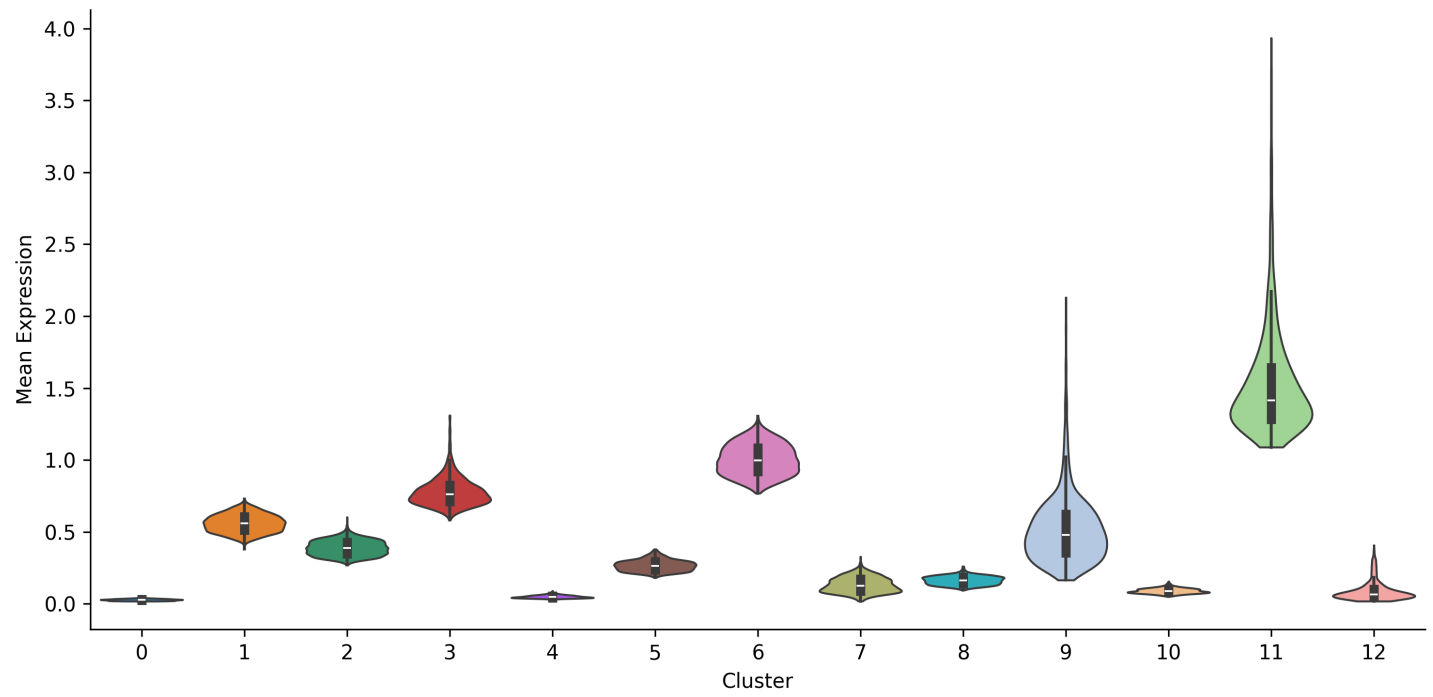

Fig Z. Distribution of mean gene expression levels across identified clusters in human lung cancer data. The violin plots visualize the density and range of mean expression values for each cluster.
