# Supplementary material for: SpatioCAD: Context-aware graph diffusion model for pinpointing spatially variable genes in heterogeneous tissues
Source: PLoS Comput Biol. 2026 Nov 2;22(11):e1014923. doi: 10.1371/journal.pcbi.1014923 (PMC13750662; doi:10.1371/journal.pcbi.1014923)
Supplement: Supplementary file 3 [file pcbi.1014923.s003.pdf]

S3 Appendix

Supplementary figures (Part II)

Supporting information for
"SpatioCAD: Context-aware graph diffusion model for pinpointing spatially variable genes in heterogeneous tissues"

Supplementary Figures

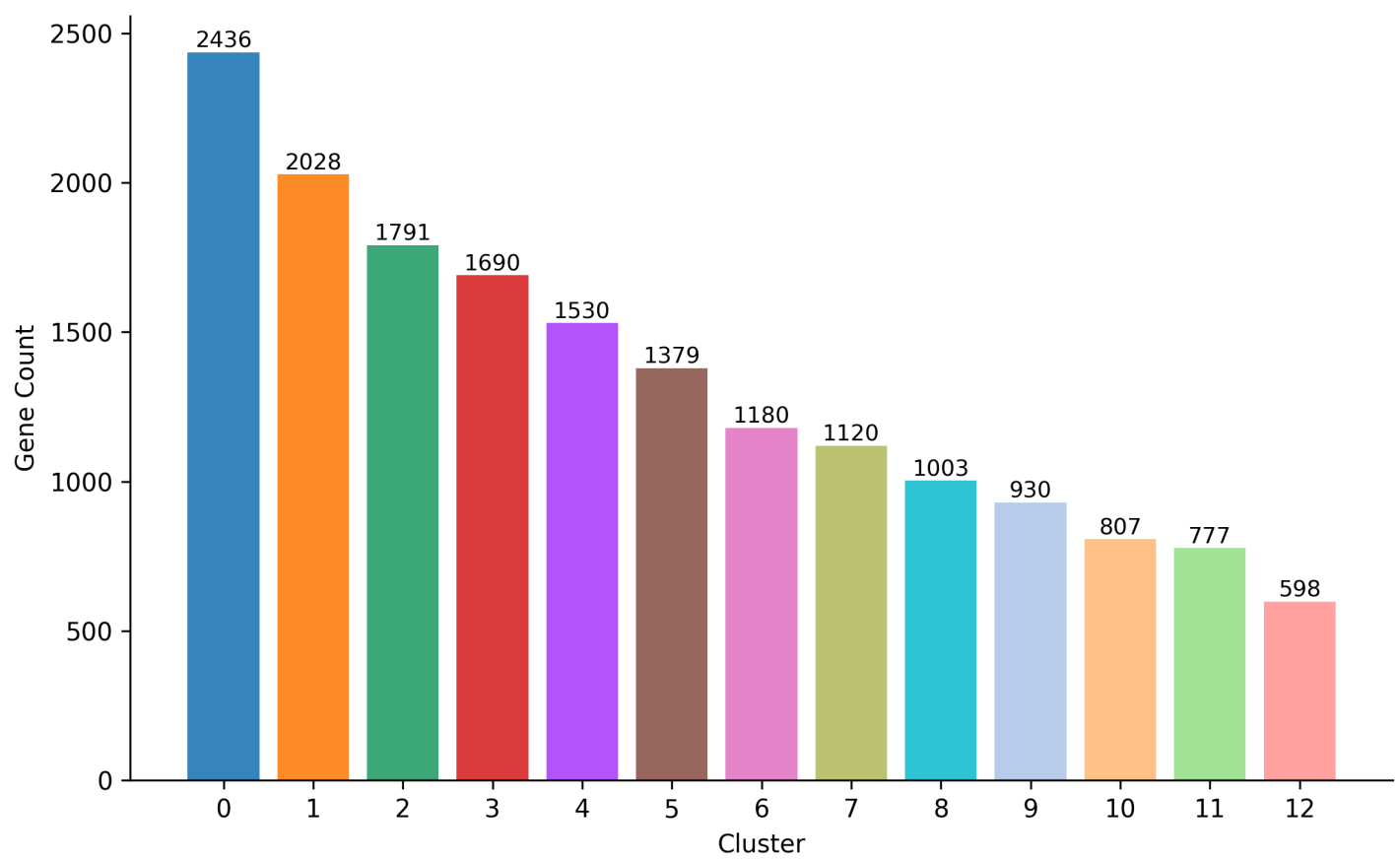

Fig A. Number of genes assigned to each identified cluster in human lung cancer data. The bar plot displays the total count of genes classified into each cluster.

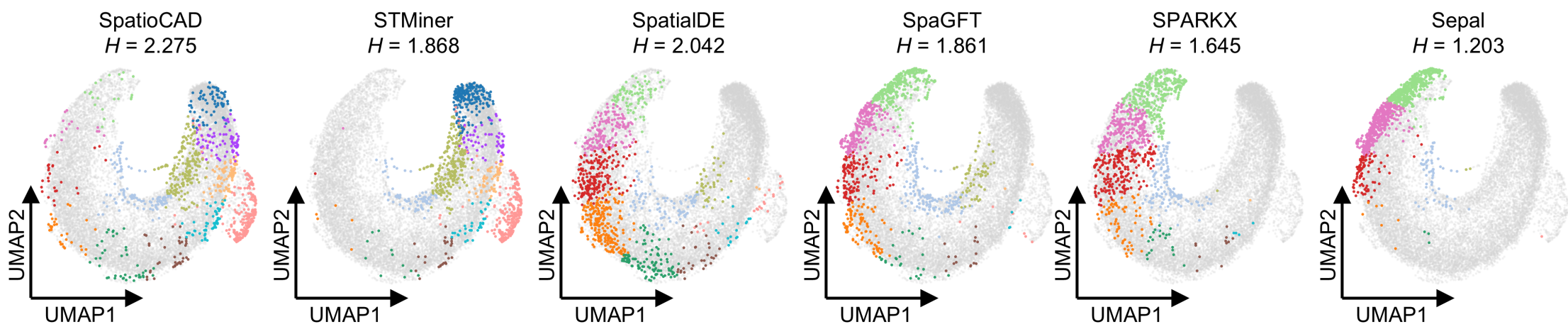

Fig B. Projection of the top 1,000 SVGs identified by each method onto the global gene UMAP in human lung cancer data. Shannon entropy of their distribution across these clusters was calculated for each method (SpatioCAD: 2.275, STMiner: 1.868, SpatialDE: 2.042, SpaGFT: 1.861, SPARK-X: 1.645, Sepal: 1.203).

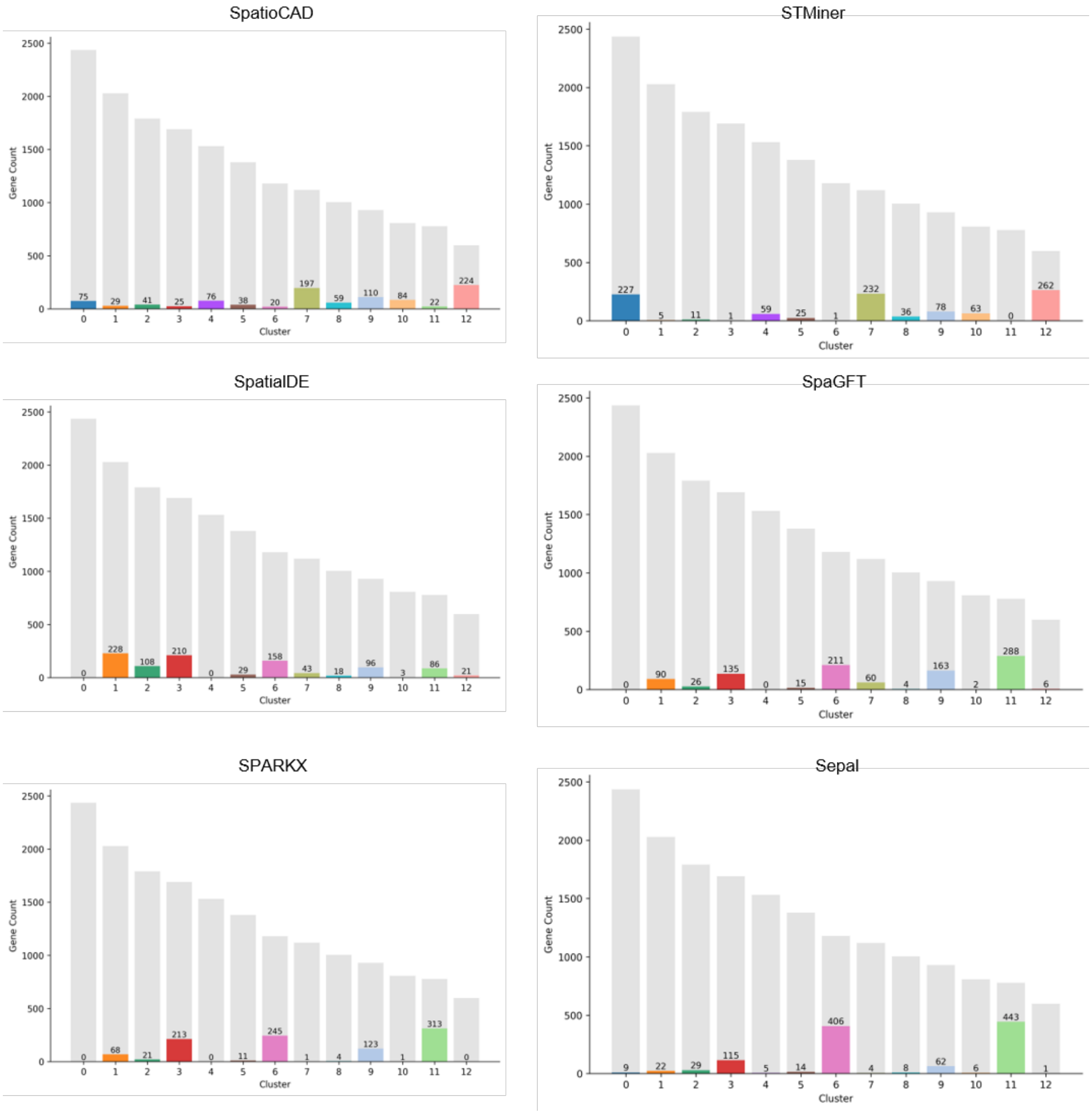

Fig C. Cluster detection preference of six methods in human lung cancer data. Each panel displays the number of genes identified by a specific method across the 13 defined clusters. The light grey bars represent the total size of each gene cluster (background). The colored bars and the numbers above them indicate the count of genes within each cluster that were identified by the respective method.

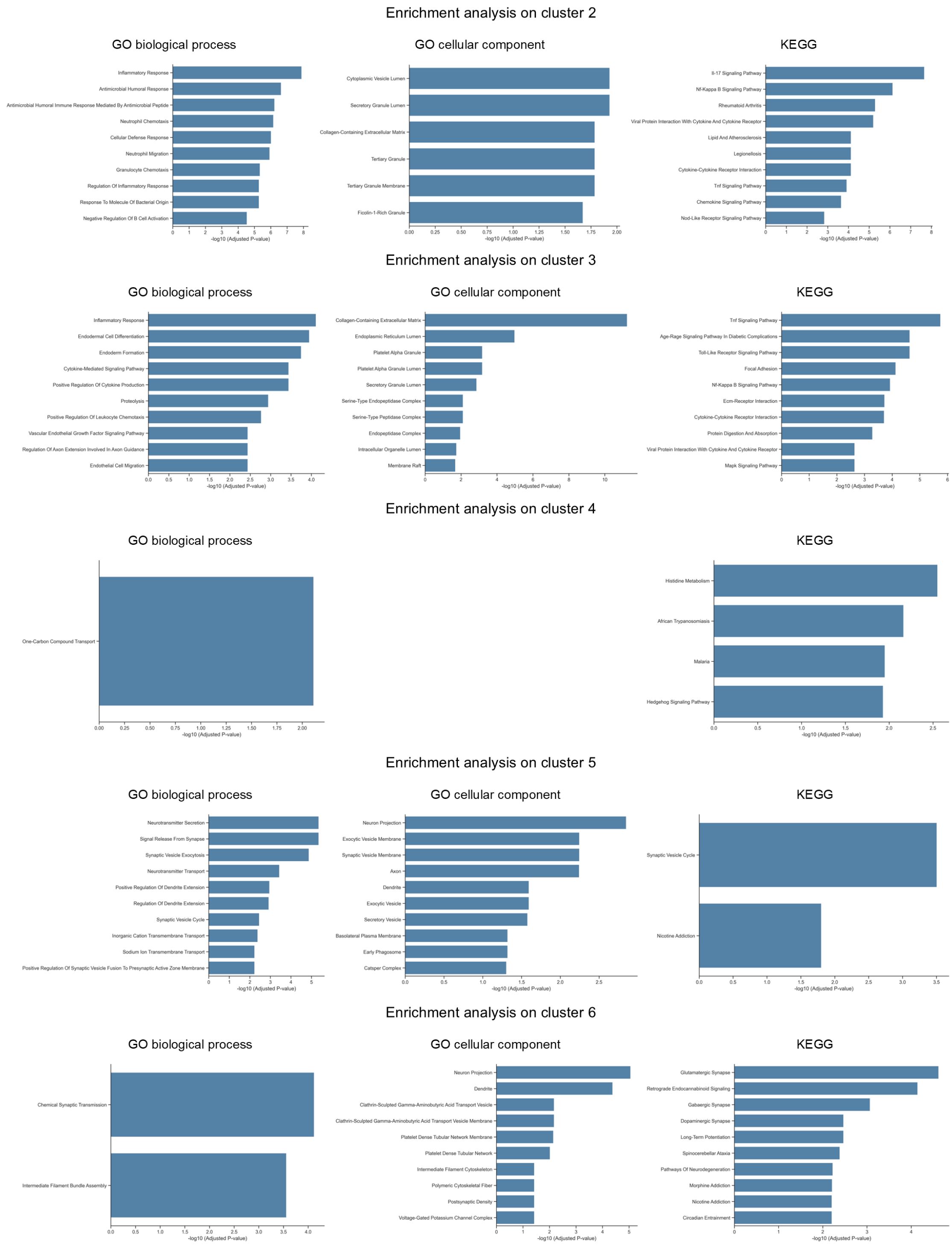

Fig D. Significantly enriched gene sets (FDR adjusted p-value < 0.05) in gene set enrichment results for the remaining clusters (Clusters 2-6) of sample DMG1 on three reference gene sets: GO biological process, GO cellular component, and KEGG.

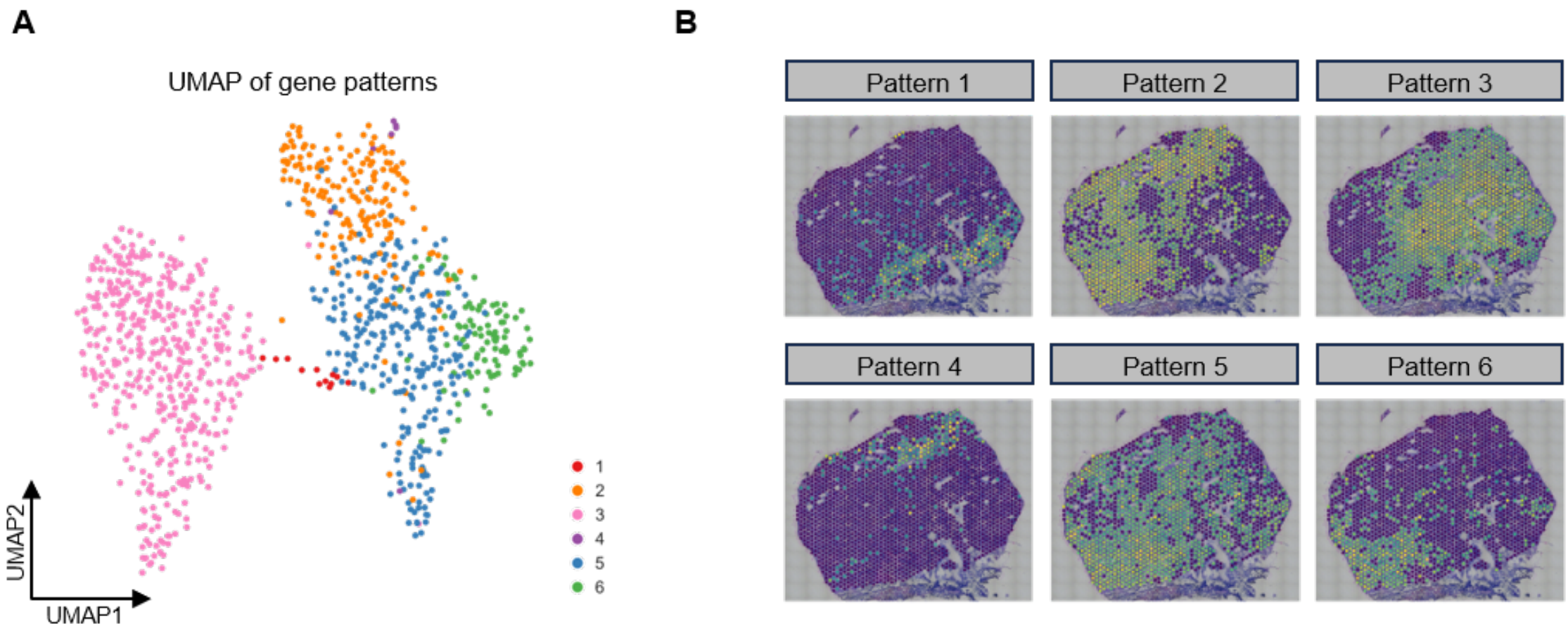

Fig E. A UMAP plot shows the gene clusters of sample GBM3 based on their spatial distribution characteristics, with genes colored by the cluster labels generated by SpatioCAD. **B** Spatial expression patterns of representative genes from each of the six identified clusters.

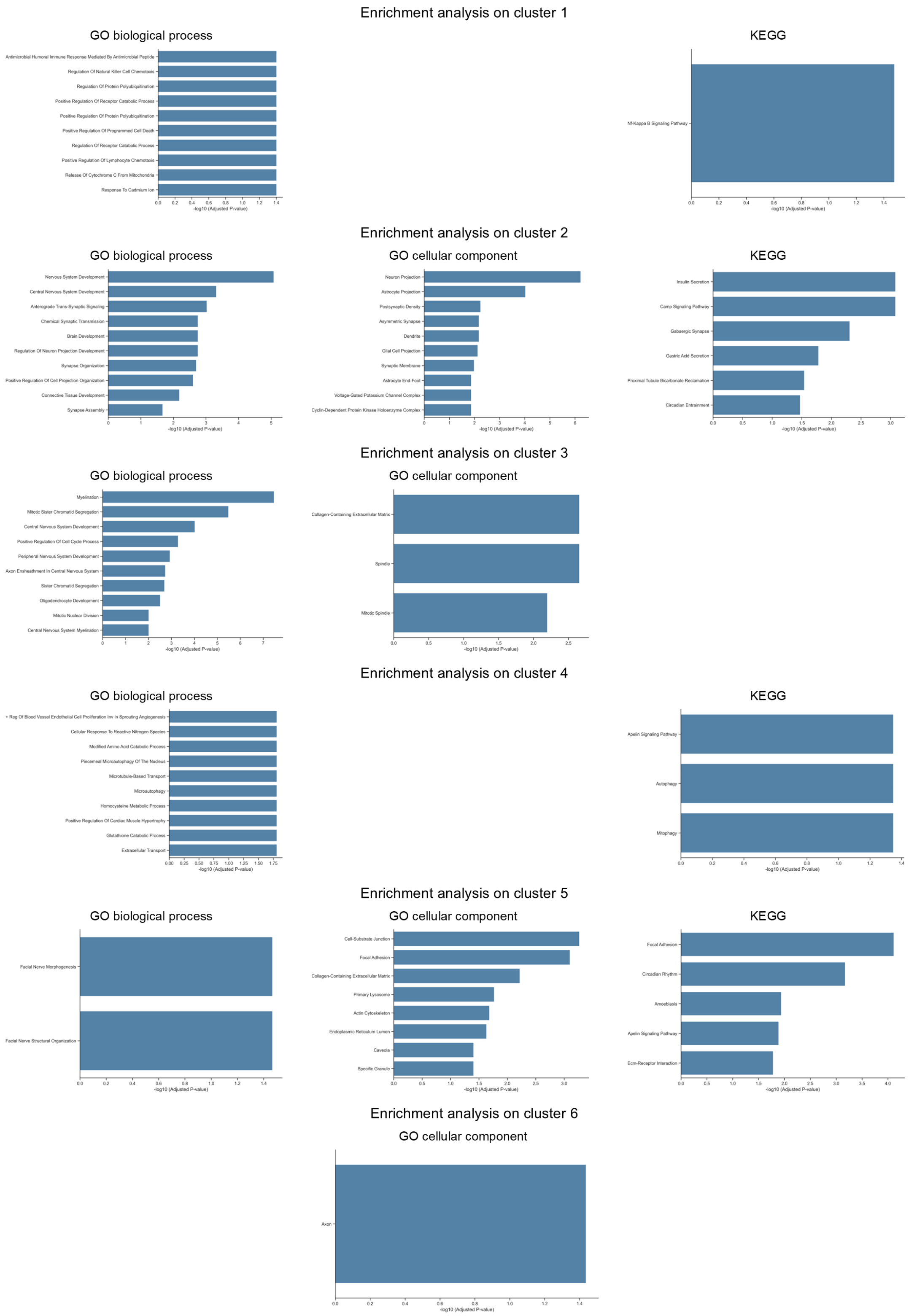

Fig F. Significantly enriched gene sets (FDR adjusted p-value < 0.05) in gene set enrichment results for all clusters (Clusters 1-6) of sample GBM3 on three reference gene sets: GO biological process, GO cellular component, and KEGG.

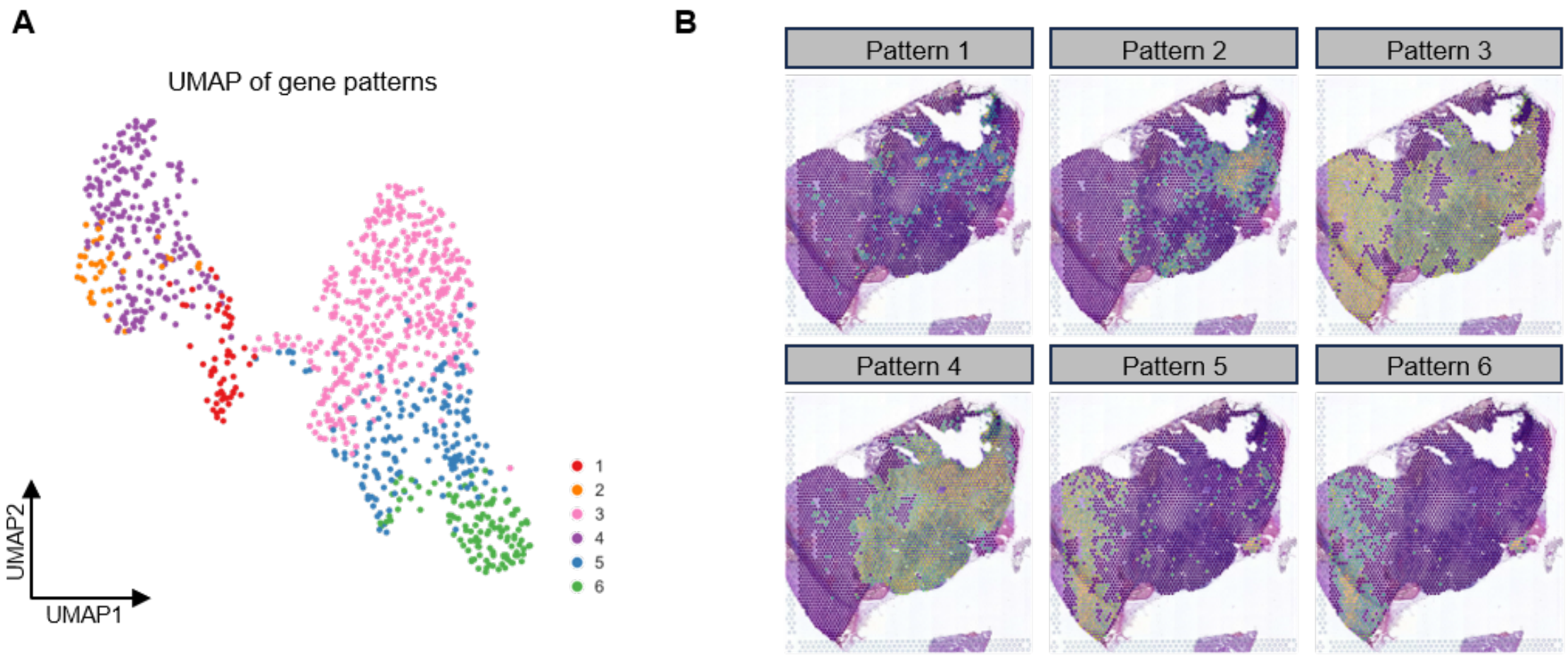

Fig G. A UMAP plot shows the gene clusters of sample GBM4 based on their spatial distribution characteristics, with genes colored by the cluster labels generated by SpatioCAD. **B** Spatial expression patterns of representative genes from each of the six identified clusters.

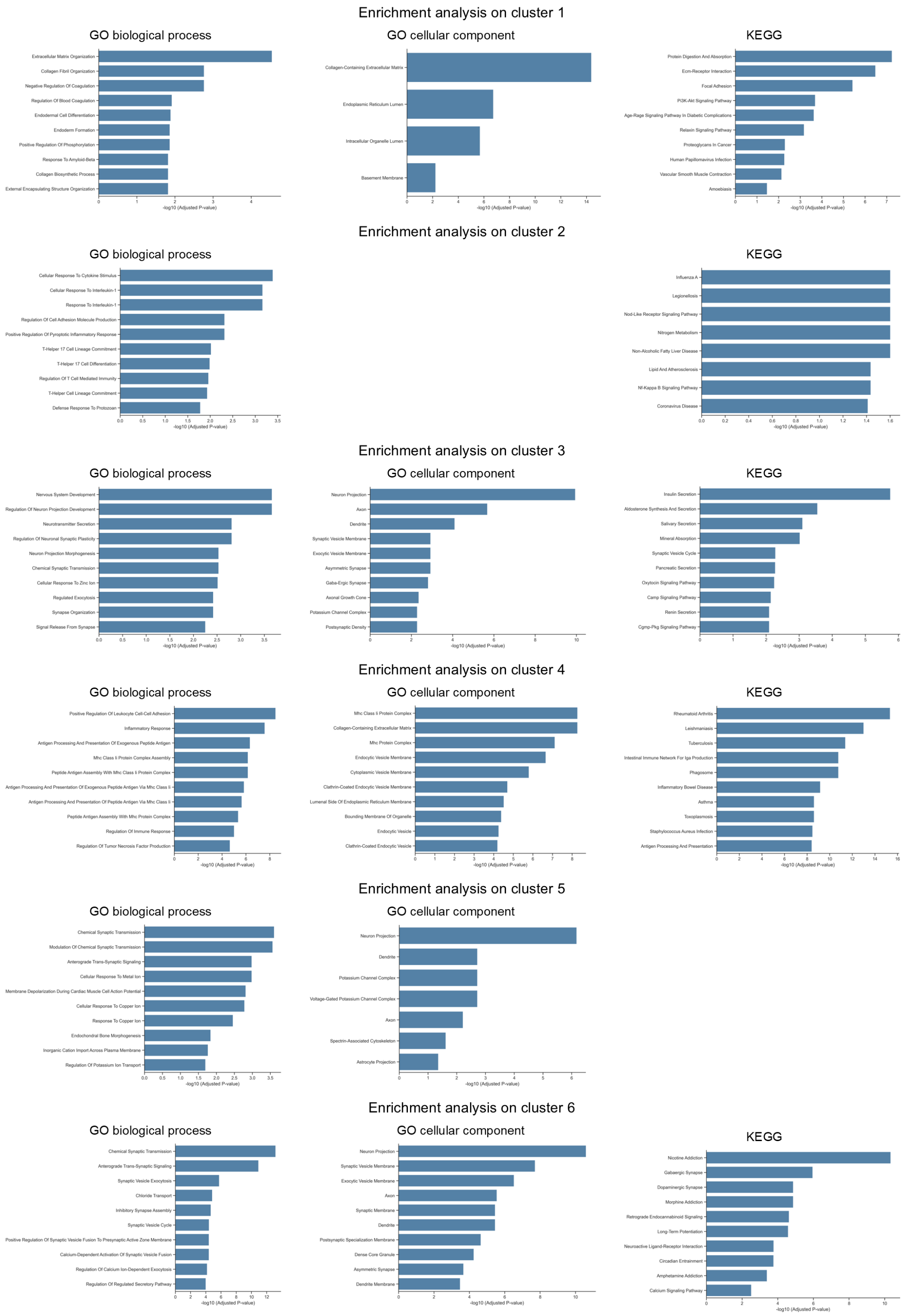

Fig H. Significantly enriched gene sets (FDR adjusted p-value < 0.05) in gene set enrichment results for all clusters (Clusters 1-6) of sample GBM4 on three reference gene sets: GO biological process, GO cellular component, and KEGG.

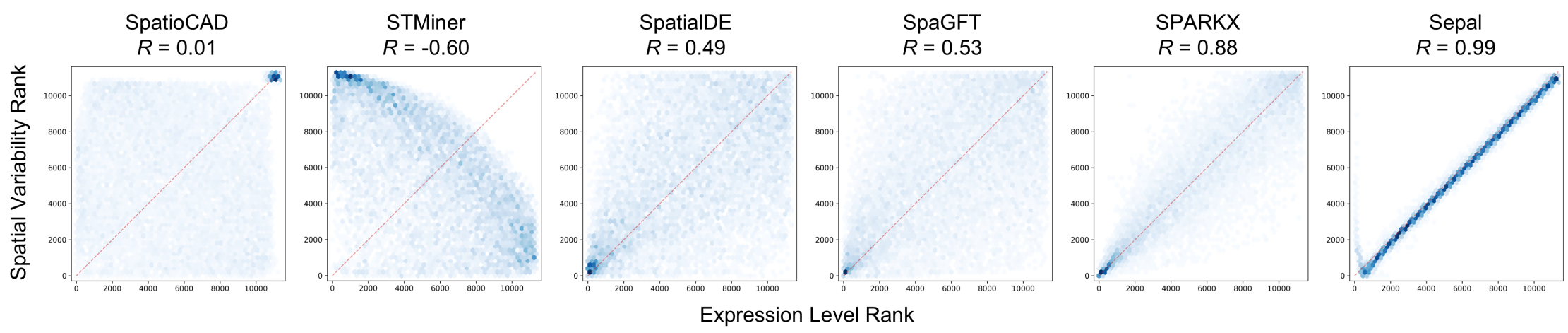

Fig I. Hexbin density plots show the relationship between gene expression ranks (x-axis) and spatial variability ranks (y-axis) in sample 151673 of DLPFC. Spearman correlation between the two ranks was calculated for each method (SpatioCAD: 0.01, STMiner: -0.60, SpatialDE: 0.49, SpaGFT: 0.53, SPARK-X: 0.88, Sepal: 0.99).

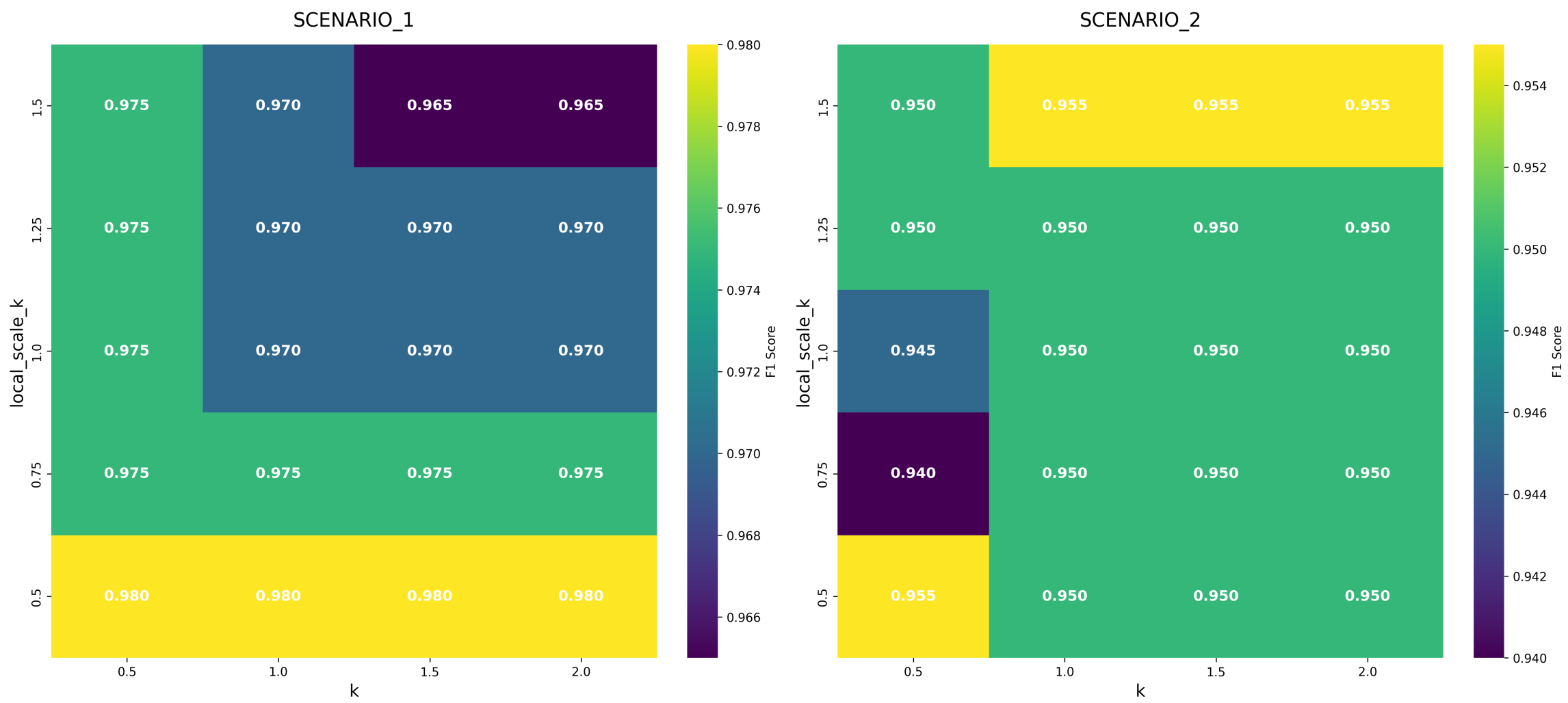

Fig J. Parameter sensitivity analysis for hyperparameters k and k'. The heatmaps display the F1 scores obtained under different combinations of k (x-axis) and k' (y-axis) in Scenario 1 (left) and Scenario 2 (right). These results represent the basis for optimal parameter selection in the respective scenarios.
